# Supplementary material for: Clinical implications of bone marrow adiposity identified by phenome-wide association and Mendelian randomization in the UK Biobank
Source: Nat Commun. 2025 Sep 23;16:8332. doi: 10.1038/s41467-025-63395-1 (PMC12457654; doi:10.1038/s41467-025-63395-1)
Supplement: Supplementary file 1 — Supplementary Information [file 41467_2025_63395_MOESM1_ESM.pdf]

# SUPPLEMENTARY INFORMATION

## Clinical implications of bone marrow adiposity identified by phenome-wide association and Mendelian randomization in the UK Biobank

Wei Xu[1,2], Ines Mesa-Eguiagaray[1], David M Morris[2,3], Chengjia Wang[3,4], Calum Gray[3], Samuel Sjöström[2], Giorgos Papanastasiou[3,5], Sammy Badr[6], Julien Paccou[6], Lijuan Wang[1], Xue Li[7], Paul R. H. J. Timmers[8], Maria Timofeeva[8,9], Scott IK Semple[2,3], Tom MacGillivray[10], Evropi Theodoratou[1,11]*, William P Cawthorn[2]*

---

**This Supplementary Information file contains the following:**

- **Supplementary Notes 1-2**
- **Supplementary Figures 1-55**
- **Supplementary References (cited in Supplementary Notes 1-2)**

---

# Supplementary Note 1

**PheCODE schema:**

Disease outcomes were defined based on the PheCODE schema (with 10,750 unique ICD-10 codes and 3,113 ICD-9 codes) using national medical records (individual level records: inpatient hospital episode records (HES), cancer registry, and death registry) until March 31, 2023. The PheCODE schema combines correlated ICD codes into a distinct code based on the Phemap v1.2 (https://phewascatalog.org/phecodes_icd10) and excludes patients with related diseases from the corresponding control groups. To account for the correlations between ICD codes, we defined the phenome framework by using the PheCODE schema that combines ≥1 related ICD codes into distinct outcome groups[1]. For a given phenotype, the case group included patients recorded as having the specific phecode that most closely related to the etiology of the disease, and the control group was defined on the basis of the absence of the phecode. Participants with a disease code that was related to 1 of the examined case group were also excluded from the control group[2]. Male- and female-specific PheCODEs were generated based on Phemap v1.2.

**Limitation of ≥1 ICD code mapping to each PheCODE:**

In this study, cases were defined based on the presence of at least one ICD code mapping to each PheCODE, consistent with our previous publications[3-7]. While this approach increases sensitivity and preserves statistical power, it has several limitations. First, it may introduce misclassification and elevate the risk of false positives, as a single ICD code can reflect provisional, erroneous, or rule-out diagnoses. Second, phenotype specificity may be reduced, since individuals with only one relevant code may not truly meet diagnostic criteria. Third, this approach lacks confirmation of chronicity or recurrence, which could be better captured by requiring multiple ICD code occurrences.

# Supplementary Note 2

**STROBE-MR checklist of recommended items to address in reports of Mendelian randomization studies**[8,9]

| Item No. | Section | Checklist item | Page No. | Relevant text from manuscript |
|---|---|---|---|---|
| 1 | **TITLE and ABSTRACT** | Indicate Mendelian randomization (MR) as the study's design in the title and/or the abstract if that is a main purpose of the study | 1, 2 | Title: Clinical implications of bone marrow adiposity identified through deep learning, phenome-wide association, and Mendelian randomization in the UK Biobank<br>Abstract: We then establish polygenic risk scores (PRSs) based on genome-wide significant susceptibility SNPs associated with BMFF and use PRS-PheWAS and Mendelian Randomization to explore potential causal association between BMFF and disease outcomes. |
| | **INTRODUCTION** | | | |
| 2 | **Background** | Explain the scientific background and rationale for the reported study. What is the exposure? Is a potential causal relationship between exposure and outcome plausible? Justify why MR is a helpful method to address the study question | 3, 4 | The physiological and pathological roles of BMAT remain largely unknown.<br>We have since applied our deep learning models to measure BMFF of the spine, femoral head, total hip, and femoral diaphysis in over 44,000 individuals and used these data for genome-wide association analyses (GWAS) to identify the genetic variants associated with altered BMFF at each site.<br>Herein, we use our site-specific BMFF measurements and newly established polygenic risk scores (PRSs) derived from our GWAS to perform phenome-wide association studies (PheWAS), thereby identifying diseases associated with altered BMFF. Finally, we use Mendelian randomization analysis (MR) to establish evidence of causality between altered BMFF and human disease. Together, these findings represent an unprecedented advance in our understanding of the clinical relevance of BMAT. |
| 3 | **Objectives** | State specific objectives clearly, including pre-specified causal hypotheses (if any). State that MR is a method that, under specific assumptions, intends to estimate causal effects | 4 | Finally, we use Mendelian randomization analysis (MR) to establish evidence of causality between altered BMFF and human disease. |

| | | | | |
|---|---|---|---|---|
| | **METHODS** | | | |
| 4 | **Study design and data sources** | Present key elements of the study design early in the article. Consider including a table listing sources of data for all phases of the study. For each data source contributing to the analysis, describe the following: | | |
| | | a) Setting: Describe the study design and the underlying population, if possible. Describe the setting, locations, and relevant dates, including periods of recruitment, exposure, follow-up, and data collection, when available. | 25 | To meet the assumptions, we obtained IVs by filtering for BMFF meta-GWAS genome-wide significant SNPs ($P<5 \times 10^{-8}$), followed by linkage disequilibrium (LD)-based clumping using the 'TwoSampleMR' R package with the following parameters: clump_kb = 10, 000, clump_$r^2$ for two thresholds ($r^2$= 0.001 or $r^2$=0.6), pop = "EUR", and excluded those SNPs associated with BMD or T2D, to ensure that any observed association between IVs and outcomes is mediated through the exposure (BMFF), maintaining the validity of causal inference.The genetic instruments for each outcome were derived from summary statistics of the most recent publicly available GWASes of European ancestry with the largest sample size, obtained from the MRC IEU GWAS database (https://gwas.mrcieu.ac.uk/). The identifier for the osteoporosis GWAS is ebi-a-GCST90038656, for the fractures GWAS is ebi-a-GCST90038703, and for the type 2 diabetes GWAS is ebi-a-GCST90018926. |
| | | b) Participants: Give the eligibility criteria, and the sources and methods of selection of participants. Report the sample size, and whether any power or sample size calculations were carried out prior to the main analysis | 23 | In sample quality control, we sub-grouped participants based on their ancestry as 'White' using the UKBB data-field 22006 ("Genetic ethnic grouping") and excluded UKBB withdrawals, individuals who were outliers for heterozygosity or missing rates, individuals with a missing rate > 0.02 on autosomes, individuals with sex discordance (between the phenotypic and genetically inferred sex), individuals who were not in a maximal set of unrelated individuals up to 3rd degree, and participants included in our meta-GWAS. The study retained 305,313 participants for femoral head, 305,453 for total hip, 306,222 for diaphysis, and 303,066 for spine (Supplementary Data 13). |
| | | c) Describe measurement, quality control and selection of genetic variants | 23, 25 | In SNP quality control, we retained significant SNPs (linkage disequilibrium LD r2 < 0.6, P-value < $5 \times 10^{-8}$) found in meta-GWAS, with missingness < 0.05, Hardy-Weinberg equilibrium (HWE) test P-value > 10–12, non-multiallelic, imputation quality (INFO) > 0.4, and minor allele frequency (MAF) > 0.005. We further excluded ambiguous AT/CG variants, and insertion/deletion polymorphisms (indels).<br><br>To meet the assumptions, we obtained IVs by filtering for BMFF meta-GWAS genome-wide significant SNPs ($P<5 \times 10^{-8}$), followed by linkage disequilibrium (LD)-based clumping using the 'TwoSampleMR' R package with the following parameters: clump_kb = 10, 000, clump_$r^2$ for two thresholds ($r^2$= 0.001 or $r^2$=0.6), pop = "EUR", and excluded those SNPs associated with BMD or T2D, to ensure that any observed association between IVs and |

| | | | | |
|---|---|---|---|---|
| | | | | outcomes is mediated through the exposure (BMFF), maintaining the validity of causal inference.<br>The genetic instruments for each outcome were derived from summary statistics of the most recent publicly available GWASes of European ancestry with the largest sample size, obtained from the MRC IEU GWAS database (https://gwas.mrcieu.ac.uk/). The identifier for the osteoporosis GWAS is ebi-a-GCST90038656, for the fractures GWAS is ebi-a-GCST90038703, and for the type 2 diabetes GWAS is ebi-a-GCST90018926. |
| | | d) For each exposure, outcome, and other relevant variables, describe methods of assessment and diagnostic criteria for diseases | 22 | We developed and validated a light-weight attention-based U-Net model for simultaneous detection and segmentation of tiny structures in large 3D MRI imaging data, to generate volumes of interest (VOIs) corresponding to bone marrow regions including femoral head, total hip, femoral diaphysis, and spine. The segmented VOIs were then applied to the fat fraction (FF) maps. The deep-learning BMFF measurements of the four bone regions were conducted in two batches based on the availability of MRI data released by UKBB (N=50,226). Method details for deep learning training, development and validation are described in our previous publication. |
| | | e) Provide details of ethics committee approval and participant informed consent, if relevant | 22 | UKBB has ethics approval from the National Health Service North-West Centre Research Ethics Committee (Ref: 11/NW/0382). Data for this work was obtained under an approved UKBB project application (ID 48697). |
| 5 | **Assumptions** | Explicitly state the three core IV assumptions for the main analysis (relevance, independence and exclusion restriction) as well assumptions for any additional or sensitivity analysis | 25 | We adhered to the three core genetic instrumental variants (IV) assumptions: (1) Relevance: the IV is associated with the exposure of interest; (2) Independence: the IV is independent of any confounders of the exposure-outcome association; and (3) Exclusion Restriction: the IV selected for MR analysis are not associated with the outcome of interest independently of the exposure. |
| 6 | **Statistical methods: main analysis** | Describe statistical methods and statistics used | | |
| | | a) Describe how quantitative variables were handled in the analyses (i.e., scale, units, model) | 25 | Here, β represents the beta coefficient of SNP on the exposure, and SE is the standard error of the beta68. The F-statistic was estimated to assess whether there was a possibility of weak instrument bias. The genetic instruments for each outcome were derived from summary statistics of the most recent publicly available GWASes of European ancestry with the largest sample size, obtained from the MRC IEU GWAS database (https://gwas.mrcieu.ac.uk/). The identifier for the osteoporosis GWAS is ebi-a-GCST90038656, for the fractures GWAS is ebi-a-GCST90038703, and for the type 2 diabetes GWAS is ebi-a-GCST90018926. |

| | | | | | |
|---|---|---|---|---|---|
| | | b) | Describe how genetic variants were handled in the analyses and, if applicable, how their weights were selected | 25 | To meet the assumptions, we obtained IVs by filtering for BMFF meta-GWAS genome-wide significant SNPs ($P<5\times10^{-8}$), followed by linkage disequilibrium (LD)-based clumping using the 'TwoSampleMR' R package with the following parameters: clump_kb = 10, 000, clump_$r^2$ for two thresholds ($r^2$= 0.001 or $r^2$=0.6), pop = "EUR", and excluded those SNPs associated with BMD or T2D, to ensure that any observed association between IVs and outcomes is mediated through the exposure (BMFF), maintaining the validity of causal inference.<br>The genetic instruments for each outcome were derived from summary statistics of the most recent publicly available GWASes of European ancestry with the largest sample size, obtained from the MRC IEU GWAS database (https://gwas.mrcieu.ac.uk/). The identifier for the osteoporosis GWAS is ebi-a-GCST90038656, for the fractures GWAS is ebi-a-GCST90038703, and for the type 2 diabetes GWAS is ebi-a-GCST90018926. |
| | | c) | Describe the MR estimator (e.g. two-stage least squares, Wald ratio) and related statistics. Detail the included covariates and, in case of two-sample MR, whether the same covariate set was used for adjustment in the two samples | 25 | In MR analyses, six methods were employed to examine the causal association between exposure and outcome, including IVW, MR-Horse, MR-Egger, weighted mode, weighted median, and simple model, among which IVW and MR-Horse were the primary methods. |
| | | d) | Explain how missing data were addressed | 25 | Summary statistics of the SNP-exposure association and the SNP-outcome association were combined to estimate the causal effect of the exposure on the outcome. SNP effect data for the exposure and outcome were harmonized to ensure consistency in effect direction and allele matching.<br>We included IVs which were present in both datasets (exposure and the outcome). |
| | | e) | If applicable, indicate how multiple testing was addressed | | |
| 7 | **Assessment of assumptions** | | Describe any methods or prior knowledge used to assess the assumptions or justify their validity | 9, 10 | We conducted two-sample MR to test if there is evidence of causality between altered BMFF and three of the diseases identified from our PheWAS analyses (Tables 5-7). Instrumental variables (IVs) were selected under two linkage disequilibrium (LD) clumping thresholds ($r^2$ <0.001 or $r^2$ <0.6) to evaluate robustness to variant correlation. Among all of the PheCODEs identified from Obs-PheWAS, PRS1-PheWAS and PRS2-PheWAS, osteoporosis was the only disease consistently associated with all four bone regions (Supplementary Data 20). Fracture-related PheCODEs were also prominent among the PRS1- and PRS2-PheWAS results, while Obs-PheWAS revealed strong associations, in varying directions, between BMFF and T2D in total hip, diaphysis and spine (Table 2). Each of these diseases (osteoporosis, fractures, and T2D) has been a focus of previous BMAT |

| | | | | |
|---|---|---|---|---|
| | | | | research and each imposes a substantial burden on public health. Therefore, we focused our MR analyses on these three diseases. |
| 8 | **Sensitivity analyses and additional analyses** | Describe any sensitivity analyses or additional analyses performed (e.g. comparison of effect estimates from different approaches, independent replication, bias analytic techniques, validation of instruments, simulations) | 25, 26 | Horizontal pleiotropy tests were performed based on (i) MR-Egger regression (intercept close to 0, P-value > 0.05: no evidence for horizontal pleiotropy), and (ii) MR-PRESSO (detected and corrected for horizontal pleiotropy by identifying outlier variants). Heterogeneity test was estimated based on Cochran's Q statistic. To further assess the robustness of the MR findings, we used the leave-one-out analysis to investigate the influence of outlying and/or pleiotropic genetic variants. In addition, we applied MR-Horse as sensitivity analysis. This Bayesian model with horseshoe shrinkage can account for valid instruments, uncorrelated and correlated pleiotropy. MR results were visualized using scatter plots (to display the consistency of causal estimates across different MR methods, and the overall direction of causality), forest plots (to present the causal effect estimates of individual SNPs), funnel plots (to detect asymmetry, which could indicate horizontal pleiotropy or publication bias), and leave-one-out plots (to test IV outliers on the causal estimates). |
| 9 | **Software and pre-registration** | | | |
| | a) | Name statistical software and package(s), including version and settings used | 26 | All tests were two-sided. MR applied the FDR-Q correction (q-value <0.05 as statistically significant). The analysis was performed using the 'TwoSampleMR', 'MR-PRESSO', and 'R2jags' packages [https://github.com/MRCIEU/TwoSampleMR] (R version 4.4.1). |
| | b) | State whether the study protocol and details were pre-registered (as well as when and where) | | Not Applicable |
| | **RESULTS** | | | |
| 10 | **Descriptive data** | | | |
| | a) | Report the numbers of individuals at each stage of included studies and reasons for exclusion. Consider use of a flow diagram | 23 | In sample quality control, we sub-grouped participants based on their ancestry as 'White' using the UKBB data-field 22006 ("Genetic ethnic grouping") and excluded UKBB withdrawals, individuals who were outliers for heterozygosity or missing rates, individuals with a missing rate > 0.02 on autosomes, individuals with sex discordance (between the phenotypic and genetically inferred sex), individuals who were not in a maximal set of unrelated individuals up to 3rd degree, and participants included in our meta-GWAS. The study retained 305,313 participants for femoral head, 305,453 for total hip, 306,222 for diaphysis, and 303,066 for spine (Supplementary Data 13). |

| | | | | |
|---|---|---|---|---|
| | | b) Report summary statistics for phenotypic exposure(s), outcome(s), and other relevant variables (e.g. means, SDs, proportions) | 10 | In the MR analysis, the proportion of variance ($R^2$) explained by the genetic variants ranged from 0.06% to 0.41% and the F-statistic ranged from 29.82 to 179.79 across the four bone regions, indicating that genetically predicted BMFF is a robust instrumental variable (IV) for the MR analysis (Supplementary Data 15, 26, 29, and 31). |
| | | c) If the data sources include meta-analyses of previous studies, provide the assessments of heterogeneity across these studies | | Not Applicable |
| | | d) For two-sample MR:<br>i. Provide justification of the similarity of the genetic variant-exposure associations between the exposure and outcome samples<br>ii. Provide information on the number of individuals who overlap between the exposure and outcome studies | 25, 26 | Horizontal pleiotropy tests were performed based on (i) MR-Egger regression (intercept close to 0, P-value > 0.05: no evidence for horizontal pleiotropy), and (ii) MR-PRESSO (detected and corrected for horizontal pleiotropy by identifying outlier variants). Heterogeneity test was estimated based on Cochran's Q statistic. To further assess the robustness of the MR findings, we used the leave-one-out analysis to investigate the influence of outlying and/or pleiotropic genetic variants. In addition, we applied MR-Horse as sensitivity analysis. This Bayesian model with horseshoe shrinkage can account for valid instruments, uncorrelated and correlated pleiotropy. |
| 11 | **Main results** | | | |
| | | a) Report the associations between genetic variant and exposure, and between genetic variant and outcome, preferably on an interpretable scale | 10, 11 | The causal association of BMFF genetic predisposition with osteoporosis is summarized in Table 5. When using the more stringent LD clumping threshold ($r^2$ <0.001), positive associations with osteoporosis were found for total hip BMFF and diaphysis BMFF based on the inverse-variance weighted (IVW) method and for diaphysis BMFF based on MR-Horse. Moreover, consistent positive associations were detected for all three femoral sites when the less-stringent LD clumping threshold of $r^2$ <0.6 was applied. However, no causal association was found in the spine, regardless of the $r^2$ threshold used. As outlined in the *Discussion*, the lack of causality for spine BMFF is surprising and could have several explanations, including osteoporosis diagnoses in UKBB being driven more by bone loss in the hip than in the spine. The Cochran's Q statistic of MR-Egger and IVW methods indicated heterogeneity. However, MR-Egger intercept in the analysis showed no horizontal pleiotropy for each bone region. The scatter plots, forest plots and funnel plots for these analyses are presented in Supplementary Figures 8-23. The results were also consistent after leave-one-out analysis (Supplementary Figures 10, 14, 18, 22; Supplementary Data 23, 26). The association of osteoporosis remained the same in the MR-PRESSO analyses (Supplementary Data 22) after removal of outliers, for the femoral sites. There was no difference in MR-PRESSO outlier correction-adjusted causal estimates. The MR-PRESSO global test indicated evidence of heterogeneity across all regions. Distortion coefficients for femoral head, total hip, and diaphysis were non-significant, suggesting more robust estimates after outlier correction. |

| | | | |
|---|---|---|---|
| | | | The causal association of BMFF genetic liability with fractures is summarized in Table 6. Each of the MR methods found no significant causal associations for any of the four bone regions when using the more stringent LD clumping threshold ($r^2$ <0.001). However, when the more lenient LD clumping threshold of $r^2$<0.6 was applied, IVW and MR-Horse showed positive associations with fractures for all three femoral sites. Horizontal pleiotropy was found only in MR for the femoral head (P = 0.015) when using the more lenient LD clumping threshold. The scatter plots, forest plots and funnel plots are presented in Supplementary Figures 24-39. The results were consistent after leave-one-out analysis (Supplementary Figures 26, 30, 34, 38; Supplementary Data 24, 29). MR-PRESSO analyses (Supplementary Data 22) also supported the positive causal associations with fractures in femoral head and total hip. MR-PRESSO global test found evidence of heterogeneity only for the diaphysis when using the more lenient LD clumping threshold.<br><br>The causal association of BMFF genetic liability with T2D is summarized in Table 7. When using the more stringent LD clumping threshold ($r^2$<0.001), none of the MR methods found significant causal associations in any of the four bone regions. At the less stringent LD clumping threshold ($r^2$<0.6), the findings suggested negative associations with T2D in the total hip and diaphysis, whereas MR-Horse observed a positive association between BMFF genetic liability and T2D in the spine. Evidence of heterogeneity was detected across all regions, but pleiotropy (Egger intercept) was not significant in most cases (only P-diaphysis =0.022 for MR using $r^2$<0.6), supporting the validity of the causal estimates. The scatter plots, forest plots and funnel plots are presented in Supplementary Figures 40-55. The results remained consistent in leave-one-out analysis (Supplementary Figures 42, 46, 50, 54; Supplementary Data 25, 31). The MR-PRESSO global test (Supplementary Data 22) indicated evidence of heterogeneity across all regions. Only the diaphysis region found a significant distortion coefficient under all $r^2$ thresholds, indicating potential influence from outliers. Overall, the association of T2D remained consistent in the MR-PRESSO analyses after removal of outliers. |
| b) | Report MR estimates of the relationship between exposure and outcome, and the measures of uncertainty from the MR analysis, on an interpretable scale, such as odds ratio or relative risk per SD difference | 10, 11 | The causal association of BMFF genetic predisposition with osteoporosis is summarized in Table 5. When using the more stringent LD clumping threshold ($r^2$ <0.001), positive associations with osteoporosis were found for total hip BMFF and diaphysis BMFF based on the inverse-variance weighted (IVW) method and for diaphysis BMFF based on MR-Horse. Moreover, consistent positive associations were detected for all three femoral sites when the less-stringent LD clumping threshold of $r^2$<0.6 was applied. However, no causal association was found in the spine, regardless of the $r^2$ threshold used. As outlined in the *Discussion*, the lack of causality for spine BMFF is surprising and could have several explanations, including osteoporosis diagnoses in UKBB being driven more by bone loss in |

the hip than in the spine. The Cochran's Q statistic of MR-Egger and IVW methods indicated heterogeneity. However, MR-Egger intercept in the analysis showed no horizontal pleiotropy for each bone region. The scatter plots, forest plots and funnel plots for these analyses are presented in Supplementary Figures 8-23. The results were also consistent after leave-one-out analysis (Supplementary Figures 10, 14, 18, 22; Supplementary Data 23, 26). The association of osteoporosis remained the same in the MR-PRESSO analyses (Supplementary Data 22) after removal of outliers, for the femoral sites. There was no difference in MR-PRESSO outlier correction-adjusted causal estimates. The MR-PRESSO global test indicated evidence of heterogeneity across all regions. Distortion coefficients for femoral head, total hip, and diaphysis were non-significant, suggesting more robust estimates after outlier correction.

The causal association of BMFF genetic liability with fractures is summarized in Table 6. Each of the MR methods found no significant causal associations for any of the four bone regions when using the more stringent LD clumping threshold ($r^2$ <0.001). However, when the more lenient LD clumping threshold of $r^2$<0.6 was applied, IVW and MR-Horse showed positive associations with fractures for all three femoral sites. Horizontal pleiotropy was found only in MR for the femoral head (P = 0.015) when using the more lenient LD clumping threshold. The scatter plots, forest plots and funnel plots are presented in Supplementary Figures 24-39. The results were consistent after leave-one-out analysis (Supplementary Figures 26, 30, 34, 38; Supplementary Data 24, 29). MR-PRESSO analyses (Supplementary Data 22) also supported the positive causal associations with fractures in femoral head and total hip. MR-PRESSO global test found evidence of heterogeneity only for the diaphysis when using the more lenient LD clumping threshold.

The causal association of BMFF genetic liability with T2D is summarized in Table 7. When using the more stringent LD clumping threshold ($r^2$<0.001), none of the MR methods found significant causal associations in any of the four bone regions. At the less stringent LD clumping threshold ($r^2$ <0.6), the findings suggested negative associations with T2D in the total hip and diaphysis, whereas MR-Horse observed a positive association between BMFF genetic liability and T2D in the spine. Evidence of heterogeneity was detected across all regions, but pleiotropy (Egger intercept) was not significant in most cases (only P-diaphysis =0.022 for MR using $r^2$ <0.6), supporting the validity of the causal estimates. The scatter plots, forest plots and funnel plots are presented in Supplementary Figures 40-55. The results remained consistent in leave-one-out analysis (Supplementary Figures 42, 46, 50, 54; Supplementary Data 25, 31). The MR-PRESSO global test (Supplementary Data 22) indicated evidence of heterogeneity across all regions. Only the diaphysis region found a significant distortion coefficient under all $r^2$ thresholds, indicating potential influence from

| | | | | | |
|---|---|---|---|---|---|
| | | | | | outliers. Overall, the association of T2D remained consistent in the MR-PRESSO analyses after removal of outliers. |
| | | c) | If relevant, consider translating estimates of relative risk into absolute risk for a meaningful time period | | Not Applicable |
| | | d) | Consider plots to visualize results (e.g. forest plot, scatterplot of associations between genetic variants and outcome versus between genetic variants and exposure) | 10, 11 | The causal association of BMFF genetic predisposition with osteoporosis is summarized in Table 5.<br>The causal association of BMFF genetic liability with fractures is summarized in Table 6.<br>The causal association of BMFF genetic liability with T2D is summarized in Table 7. |
| 12 | **Assessment of assumptions** | | | | |
| | | a) | Report the assessment of the validity of the assumptions | 10, 11 | The causal association of BMFF genetic predisposition with osteoporosis is summarized in Table 5. When using the more stringent LD clumping threshold ($r^2$ <0.001), positive associations with osteoporosis were found for total hip BMFF and diaphysis BMFF based on the inverse-variance weighted (IVW) method and for diaphysis BMFF based on MR-Horse. Moreover, consistent positive associations were detected for all three femoral sites when the less-stringent LD clumping threshold of $r^2$ <0.6 was applied. However, no causal association was found in the spine, regardless of the $r^2$ threshold used. As outlined in the *Discussion*, the lack of causality for spine BMFF is surprising and could have several explanations, including osteoporosis diagnoses in UKBB being driven more by bone loss in the hip than in the spine. The Cochran's Q statistic of MR-Egger and IVW methods indicated heterogeneity. However, MR-Egger intercept in the analysis showed no horizontal pleiotropy for each bone region. The scatter plots, forest plots and funnel plots for these analyses are presented in Supplementary Figures 8-23.<br><br>The causal association of BMFF genetic liability with fractures is summarized in Table 6. Each of the MR methods found no significant causal associations for any of the four bone regions when using the more stringent LD clumping threshold ($r^2$ <0.001). However, when the more lenient LD clumping threshold of $r^2$<0.6 was applied, IVW and MR-Horse showed positive associations with fractures for all three femoral sites. Horizontal pleiotropy was found only in MR for the femoral head (P = 0.015) when using the more lenient LD clumping threshold. The scatter plots, forest plots and funnel plots are presented in Supplementary Figures 24-39. |

| | | | |
|---|---|---|---|
| | | | The causal association of BMFF genetic liability with T2D is summarized in Table 7. When using the more stringent LD clumping threshold ($r^2$<0.001), none of the MR methods found significant causal associations in any of the four bone regions. At the less stringent LD clumping threshold ($r^2$ <0.6), the findings suggested negative associations with T2D in the total hip and diaphysis, whereas MR-Horse observed a positive association between BMFF genetic liability and T2D in the spine. Evidence of heterogeneity was detected across all regions, but pleiotropy (Egger intercept) was not significant in most cases (only P-diaphysis =0.022 for MR using $r^2$ <0.6), supporting the validity of the causal estimates. The scatter plots, forest plots and funnel plots are presented in Supplementary Figures 40-55. |
| b) | Report any additional statistics (e.g., assessments of heterogeneity across genetic variants, such as $I^2$, Q statistic or E-value) | 10, 11 | The causal association of BMFF genetic predisposition with osteoporosis is summarized in Table 5. When using the more stringent LD clumping threshold ($r^2$ <0.001), positive associations with osteoporosis were found for total hip BMFF and diaphysis BMFF based on the inverse-variance weighted (IVW) method and for diaphysis BMFF based on MR-Horse. Moreover, consistent positive associations were detected for all three femoral sites when the less-stringent LD clumping threshold of $r^2$ <0.6 was applied. However, no causal association was found in the spine, regardless of the $r^2$ threshold used. As outlined in the *Discussion*, the lack of causality for spine BMFF is surprising and could have several explanations, including osteoporosis diagnoses in UKBB being driven more by bone loss in the hip than in the spine. The Cochran's Q statistic of MR-Egger and IVW methods indicated heterogeneity. However, MR-Egger intercept in the analysis showed no horizontal pleiotropy for each bone region. The scatter plots, forest plots and funnel plots for these analyses are presented in Supplementary Figures 8-23.<br><br>The causal association of BMFF genetic liability with fractures is summarized in Table 6. Each of the MR methods found no significant causal associations for any of the four bone regions when using the more stringent LD clumping threshold ($r^2$ <0.001). However, when the more lenient LD clumping threshold of $r^2$ <0.6 was applied, IVW and MR-Horse showed positive associations with fractures for all three femoral sites. Horizontal pleiotropy was found only in MR for the femoral head (P = 0.015) when using the more lenient LD clumping threshold. The scatter plots, forest plots and funnel plots are presented in Supplementary Figures 24-39.<br><br>The causal association of BMFF genetic liability with T2D is summarized in Table 7. When using the more stringent LD clumping threshold ($r^2$<0.001), none of the MR methods found significant causal associations in any of the four bone regions. At the less stringent LD clumping threshold ($r^2$ <0.6), the findings suggested negative associations with T2D in the total hip and diaphysis, whereas MR-Horse observed a positive association between BMFF genetic liability and T2D in the spine. Evidence of heterogeneity was detected across all |

| | | | | | |
|---|---|---|---|---|---|
| | | | | | regions, but pleiotropy (Egger intercept) was not significant in most cases (only P-diaphysis =0.022 for MR using $r^2$ <0.6), supporting the validity of the causal estimates. The scatter plots, forest plots and funnel plots are presented in Supplementary Figures 40-55. |
| 13 | **Sensitivity analyses and additional analyses** | | | | |
| | | a) | Report any sensitivity analyses to assess the robustness of the main results to violations of the assumptions | 10, 11 | The results were also consistent after leave-one-out analysis (Supplementary Figures 10, 14, 18, 22; Supplementary Data 23, 26). The association of osteoporosis remained the same in the MR-PRESSO analyses (Supplementary Data 22) after removal of outliers, for the femoral sites. There was no difference in MR-PRESSO outlier correction-adjusted causal estimates. The MR-PRESSO global test indicated evidence of heterogeneity across all regions. Distortion coefficients for femoral head, total hip, and diaphysis were non-significant, suggesting more robust estimates after outlier correction.<br><br>The results were consistent after leave-one-out analysis (Supplementary Figures 26, 30, 34, 38; Supplementary Data 24, 29). MR-PRESSO analyses (Supplementary Data 22) also supported the positive causal associations with fractures in femoral head and total hip. MR-PRESSO global test found evidence of heterogeneity only for the diaphysis when using the more lenient LD clumping threshold.<br><br>The results remained consistent in leave-one-out analysis (Supplementary Figures 42, 46, 50, 54; Supplementary Data 25, 31). The MR-PRESSO global test (Supplementary Data 22) indicated evidence of heterogeneity across all regions. Only the diaphysis region found a significant distortion coefficient under all $r^2$ thresholds, indicating potential influence from outliers. Overall, the association of T2D remained consistent in the MR-PRESSO analyses after removal of outliers. |
| | | b) | Report results from other sensitivity analyses or additional analyses | 10, 11 | The results were also consistent after leave-one-out analysis (Supplementary Figures 10, 14, 18, 22; Supplementary Data 23, 36). The association of osteoporosis remained the same in the MR-PRESSO analyses (Supplementary Data 22) after removal of outliers, for the femoral sites. There was no difference in MR-PRESSO outlier correction-adjusted causal estimates. The MR-PRESSO global test indicated evidence of heterogeneity across all regions. Distortion coefficients for femoral head, total hip, and diaphysis were non-significant, suggesting more robust estimates after outlier correction. |

| | | | | |
|---|---|---|---|---|
| | | | | The results were consistent after leave-one-out analysis (Supplementary Figures 26, 30, 34, 38; Supplementary Data 24, 29). MR-PRESSO analyses (Supplementary Data 22) also supported the positive causal associations with fractures in femoral head and total hip. MR-PRESSO global test found evidence of heterogeneity only for the diaphysis when using the more lenient LD clumping threshold.<br><br>The results remained consistent in leave-one-out analysis (Supplementary Figures 42, 46, 50, 54; Supplementary Data 25, 31). The MR-PRESSO global test (Supplementary Data 22) indicated evidence of heterogeneity across all regions. Only the diaphysis region found a significant distortion coefficient under all $r^2$ thresholds, indicating potential influence from outliers. Overall, the association of T2D remained consistent in the MR-PRESSO analyses after removal of outliers. |
| | | c) Report any assessment of direction of causal relationship (e.g., bidirectional MR) | | We did not perform bidirectional MR |
| | | d) When relevant, report and compare with estimates from non-MR analyses | | Not Applicable |
| | | e) Consider additional plots to visualize results (e.g., leave-one-out analyses) | 10, 11 | Osteoporosis: The results were also consistent after leave-one-out analysis (Supplementary Figures 10, 14, 18, 22; Supplementary Data 23, 36).<br>Fractures: The results were consistent after leave-one-out analysis (Supplementary Figures 26, 30, 34, 38; Supplementary Data 24, 29).<br>Type 2 diabetes: The results remained consistent in leave-one-out analysis (Supplementary Figures 42, 46, 50, 54; Supplementary Data 25, 31). |
| | **DISCUSSION** | | | |
| 14 | **Key results** | Summarize key results with reference to study objectives | 12 | Finally, our MR studies reveal that genetic predisposition to increased BMFF at the total hip and femoral diaphysis is causally associated with osteoporosis and suggest that spine and femoral BMFF may also be causally associated with fractures and T2D. |
| 15 | **Limitations** | Discuss limitations of the study, taking into account the validity of the IV assumptions, other sources of potential bias, and imprecision. Discuss both direction and magnitude of any potential bias and any efforts to address them | 20 | MR relies on genetic instruments (SNPs) associated with BMFF, but the proportion of variance (R2) explained by the genetic instruments ranged from 0.06% to 0.41% and the F-statistic ranged from 29.82 to 179.79 across the four bone regions. This limited variance suggests potential weak instrument bias, which could lead to underpowered or biased causal estimates. |
| 16 | **Interpretation** | | | |

| | | | |
|---|---|---|---|
| a) | Meaning: Give a cautious overall interpretation of results in the context of their limitations and in comparison with other studies | 13, 14, 15 | Notably, relatively few studies measure bone marrow adiposity in the femoral diaphysis, instead focusing on the spine and proximal femur. These site-specific differences may also help to inform the potential of using bone marrow adiposity to improve clinical management of osteoporosis.<br><br>This underscores the concept that factors influencing bone quality, beyond BMD, contribute to fracture pathogenesis. It will be important for future studies to establish how increased BMFF at each site impacts bone quality and thereby contributes to fracture risk, and if bone marrow adiposity, and/or its genetic surrogates, can improve fracture prediction.<br><br>Our MR results further suggest that genetic predisposition to increased BMFF at the spine, total hip, and diaphysis has direct causal associations with T2D that mirror the site-specific PheWAS relationships. Importantly, these causal associations are significant only when using the more lenient LD threshold, again underscoring the need for future, larger-scale BMFF GWAS to allow stronger IVs to be established. Nevertheless, it is intriguing to speculate how femoral and spinal BMFF might exert divergent effects on the pathogenesis of T2D. One possibility is that femoral BMAT is a site of safer, more metabolically inert lipid storage, analogous to gluteo-femoral adipose tissue, whereas spinal BMAT is akin to more metabolically active visceral adipose tissue, releasing metabolites and endocrine factors that promote insulin resistance. Clearly, it will be important to establish the differences between spinal and femoral BMAT, both on molecular and functional levels, to better understand the relationships between BMAT and T2D. |
| b) | Mechanism: Discuss underlying biological mechanisms that could drive a potential causal relationship between the investigated exposure and the outcome, and whether the gene-environment equivalence assumption is reasonable. Use causal language carefully, clarifying that IV estimates may provide causal effects only under certain assumptions | 20 | The MR analysis assumes that the genetic instruments affect the outcome (osteoporosis, fractures, T2D) only through the exposure (BMFF). However, horizontal pleiotropy, where genetic variants influence the outcome through pathways other than BMFF, cannot be entirely ruled out. Our study used MR-Egger and MR-PRESSO to test for pleiotropy. MR-Egger and MR-PRESSO tests suggested minimal pleiotropy, the presence of heterogeneity across different bone regions suggested some genetic variants may have pleiotropic effects, potentially biasing the causal estimates. However, a major strength of our MR analysis is that we also used MR-HORSE, which is protected from bias due to pleiotropy |
| c) | Clinical relevance: Discuss whether the results have clinical or public policy relevance, and to what extent they inform effect sizes of possible interventions | 21 | The unexpected breadth of BMFF-associated diseases highlights the potential of BMFF as a biomarker for the improved prediction, prevention and treatment of diverse diseases. This includes leveraging our BMFF PRSs as genetic biomarkers, which could benefit clinical practice by eliminating the need for expensive and time-consuming MRI analyses. Our results provide evidence of causality for altered bone marrow adiposity on osteoporosis, fracture, and T2D, which suggests that modulating bone marrow adiposity could be a |

| | | | | |
|---|---|---|---|---|
| | | | | viable therapeutic strategy for the prevention and treatment of these diseases. Together, our study reveals extensive new insights into BMAT function that have the potential to benefit human health. |
| 17 | **Generalizability** | Discuss the generalizability of the study results (a) to other populations, (b) across other exposure periods/timings, and (c) across other levels of exposure | 20 | the MR analysis was conducted in a predominantly white European population from the UK Biobank, which may limit the generalizability of the findings to other ethnic groups. |
| | **OTHER INFORMATION** | | | |
| 18 | **Funding** | Describe sources of funding and the role of funders in the present study and, if applicable, sources of funding for the databases and original study or studies on which the present study is based | 27 | This work was supported by grants from the Medical Research Council (MR/S010505/1 to W.P.C. and E.T.) and the British Heart Foundation (RE/18/5/34216 for salary support to W.X.; RG/16/10/32375 to support C.W.; 4-year PhD studentship FS/4yPhD/F/22/34175C for S.S.). W.P.C. was further supported by a Chancellor's Fellowship from the University of Edinburgh. C.G. and T.M. were supported by the Edinburgh Clinical Research Facility and NHS Lothian R&D. E.T. was supported by a Cancer Research UK Career Development Fellowship (C31250/A22804). |
| 19 | **Data and data sharing** | Provide the data used to perform all analyses or report where and how the data can be accessed, and reference these sources in the article. Provide the statistical code needed to reproduce the results in the article, or report whether the code is publicly accessible and if so, where | 26 | See the Data availability and statement. |
| 20 | **Conflicts of Interest** | All authors should declare all potential conflicts of interest | 27 | G.P. is currently an employee of Pfizer; however, Pfizer had no role in the design or interpretation of this research. All other authors declare no competing interests. |

This checklist is copyrighted by the Equator Network under the Creative Commons Attribution 3.0 Unported (CC BY 3.0) license.

# Supplementary Figures

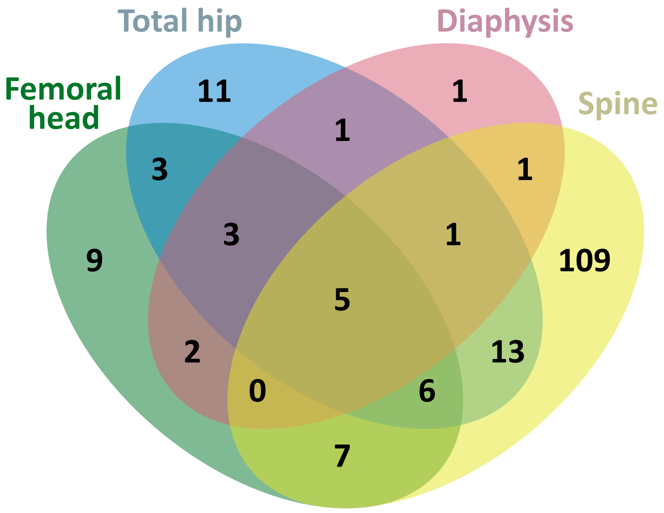


**4 regions**

- Type 2 diabetes
- Gout
- Essential hypertension
- Osteoporosis NOS
- Fracture of radius and ulna

**3 regions**

- Uterine leiomyoma
- Obesity
- Sleep apnoea
- Diverticulitis
- Cholelithiasis
- Acute renal failure
- Polyp of corpus uteri
- Postmenopausal bleeding
- Arthropathy NOS
- Other disorders of bone and cartilage

**Supplementary Figure 1 –** Overlap of disease outcomes (PheCODEs) associated with BMFF for each bone region in the Obs-PheWAS (sensitivity analysis: incident and prevalent cases)**.**

# Supplementary Figure 2

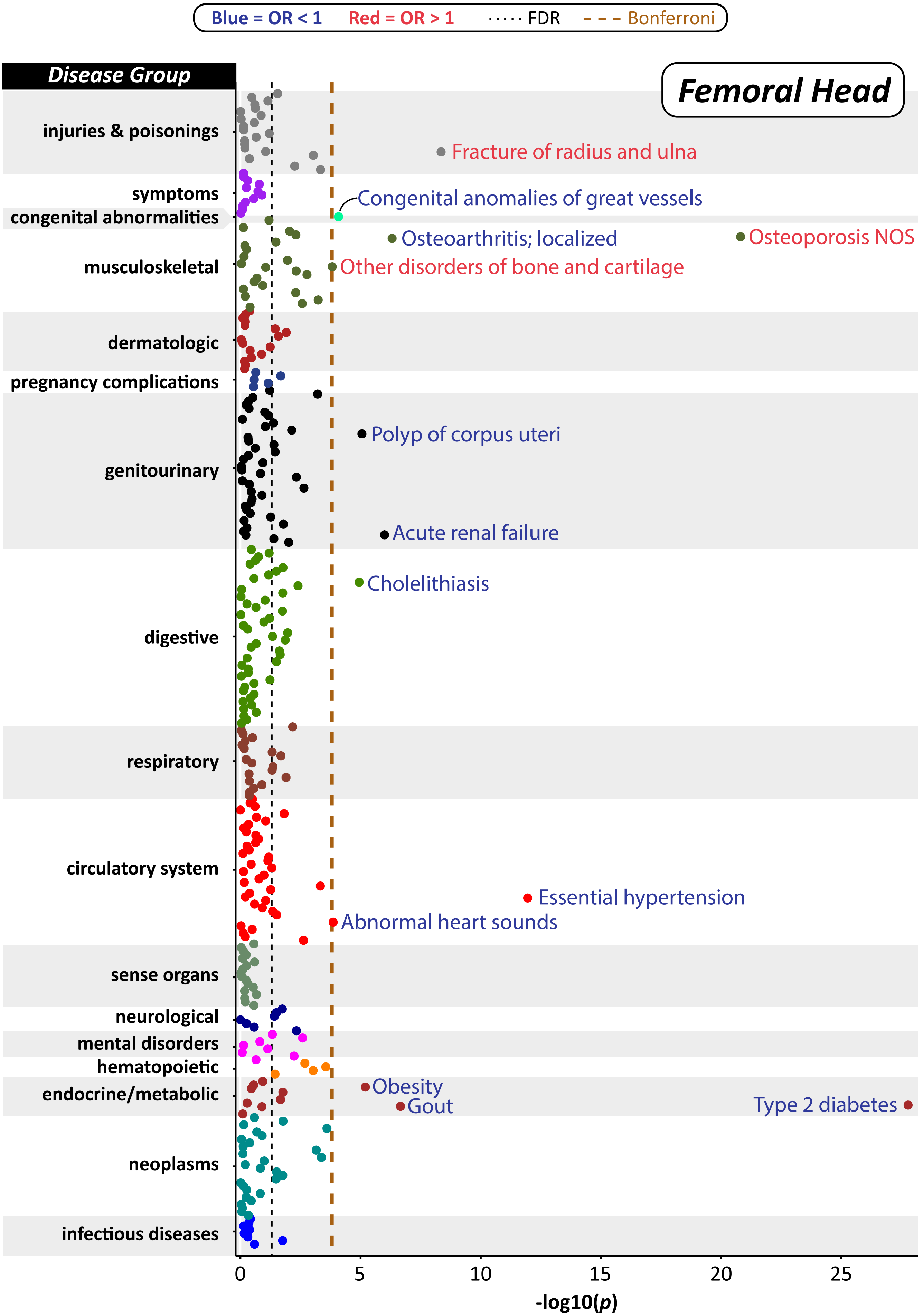

**Supplementary Figure 2 –** Manhattan plot of the Obs-PheWAS (sensitivity analysis: incident+prevalent) results for femoral head BMFF (n=45,288).

The y axes represent phenotypes (aggregated on International Classification of Disease codes), and the x axes represent the −log10 p values of two-sided test for logistic regression between BMFF and each of the phenotypes. Each dot represents one phenotype, and the colours indicate their according categories. Diseases in red have an OR >1 and those in blue have OR <1. The dotted orange line indicates a Bonferroni-correction and the dotted black line indicates an FDR-q correction. A total of 12 and 35 significant phenotypes passed Bonferroni-correction and FDR-q correction respectively. [please see Supplementary Data 5 for details]

# Supplementary Figure 3

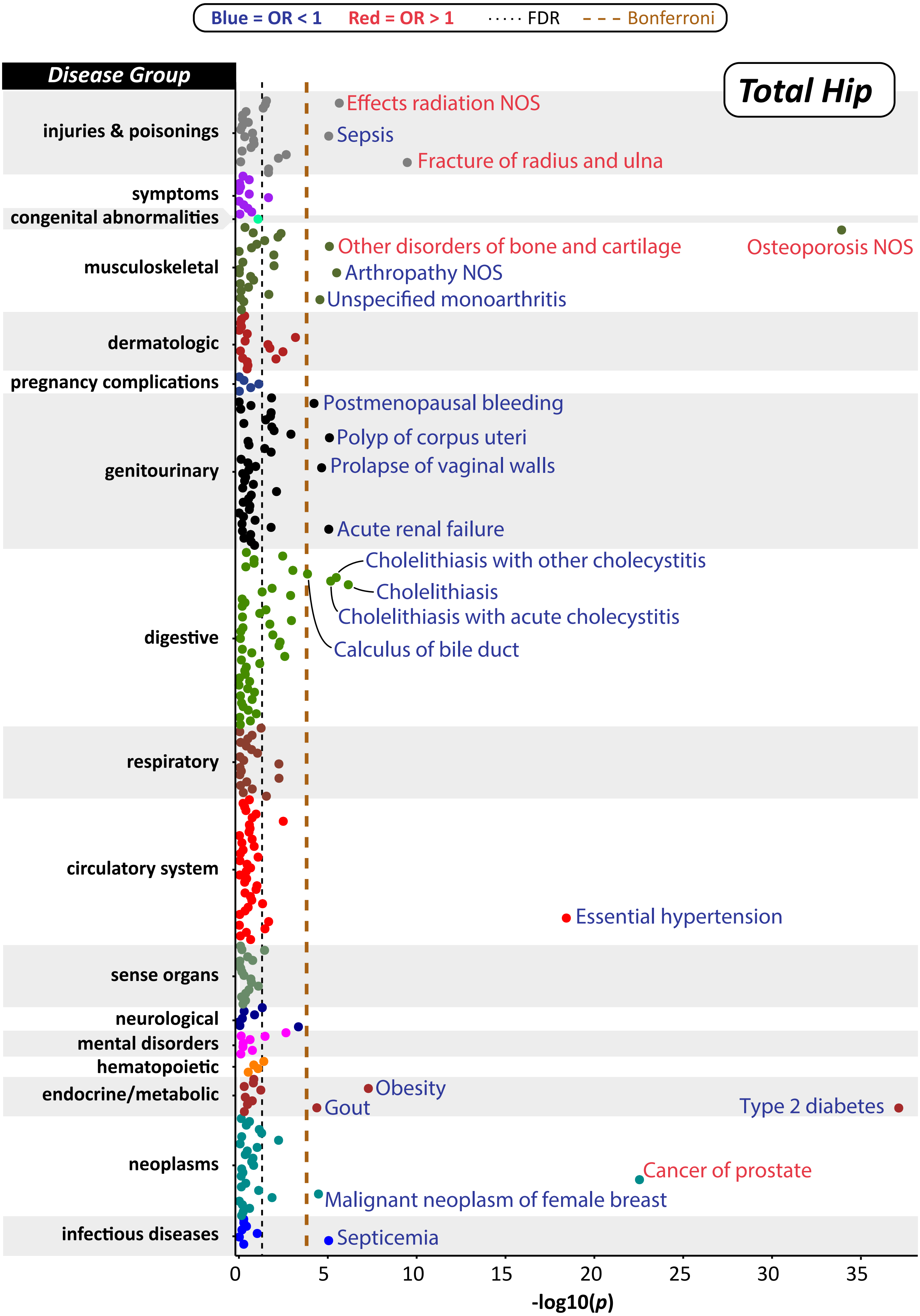

**Supplementary Figure 3 –** Manhattan plot of the Obs-PheWAS (sensitivity analysis: incident+prevalent) results for total hip BMFF (n=45,187).

The y axes represent phenotypes (aggregated on International Classification of Disease codes), and the x axes represent the −log10 p values of two-sided test for logistic regression between BMFF and each of the phenotypes. Each dot represents one phenotype, and the colours indicate their according categories. Diseases in red have an OR >1 and those in blue have OR <1. The dotted orange line indicates a Bonferroni-correction and the dotted black line indicates an FDR-q correction. A total of 22 and 43 significant phenotypes passed Bonferroni-correction and FDR-q correction respectively. [please see Supplementary Data 5 for details]

# Supplementary Figure 4

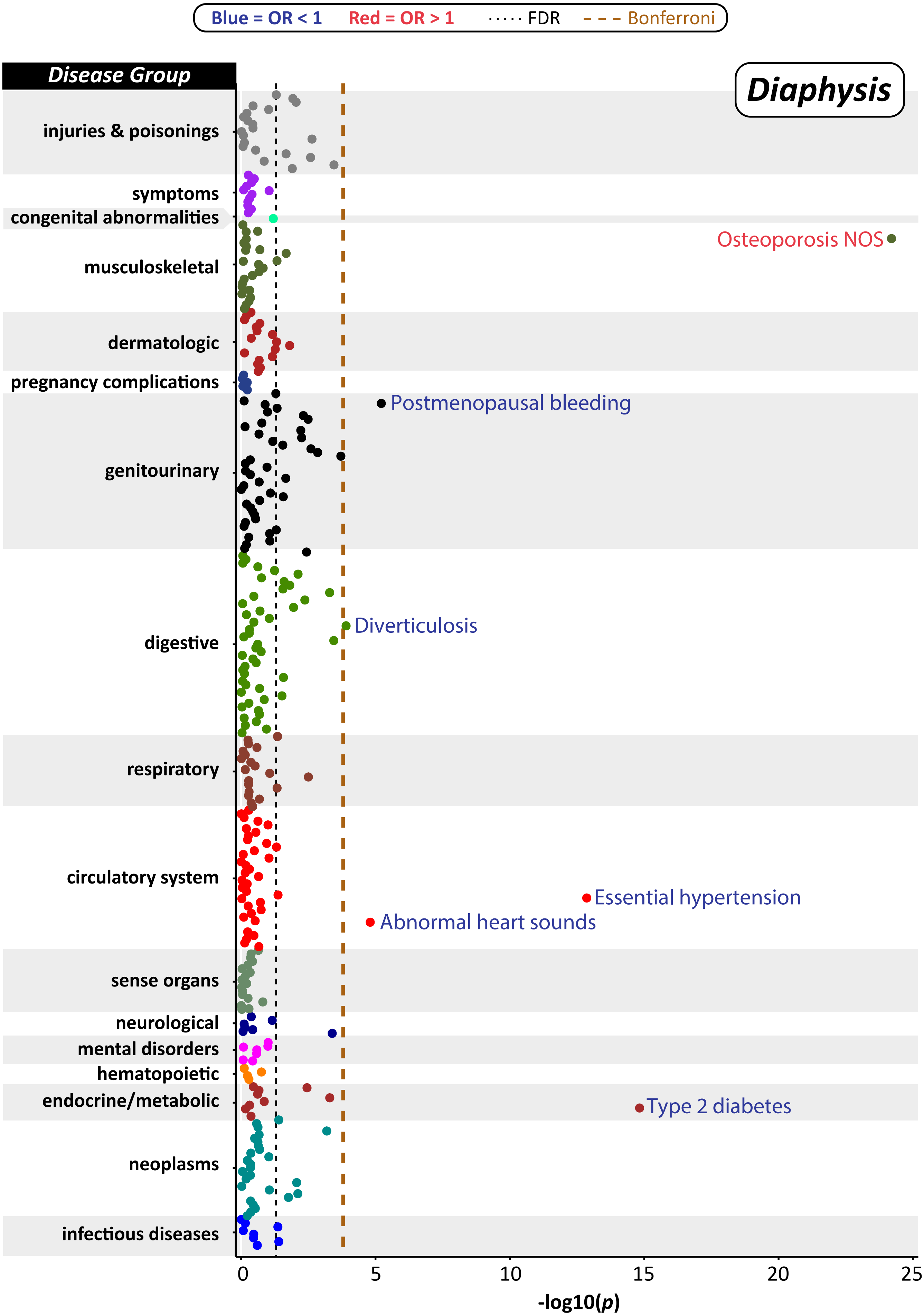

**Supplementary Figure 4 –** Manhattan plot of the Obs-PheWAS (sensitivity analysis: incident+prevalent) results for diaphysis BMFF (n=44,099).

The y axes represent phenotypes (aggregated on International Classification of Disease codes), and the x axes represent the −log10 p values of two-sided test for logistic regression between BMFF and each of the phenotypes. Each dot represents one phenotype, and the colours indicate their according categories. Diseases in red have an OR >1 and those in blue have OR <1. The dotted orange line indicates a Bonferroni-correction and the dotted black line indicates an FDR-q correction. A total of 6 and 14 significant phenotypes passed Bonferroni-correction and FDR-q correction respectively. [please see Supplementary Data 5 for details]

## Supplementary Figure 5

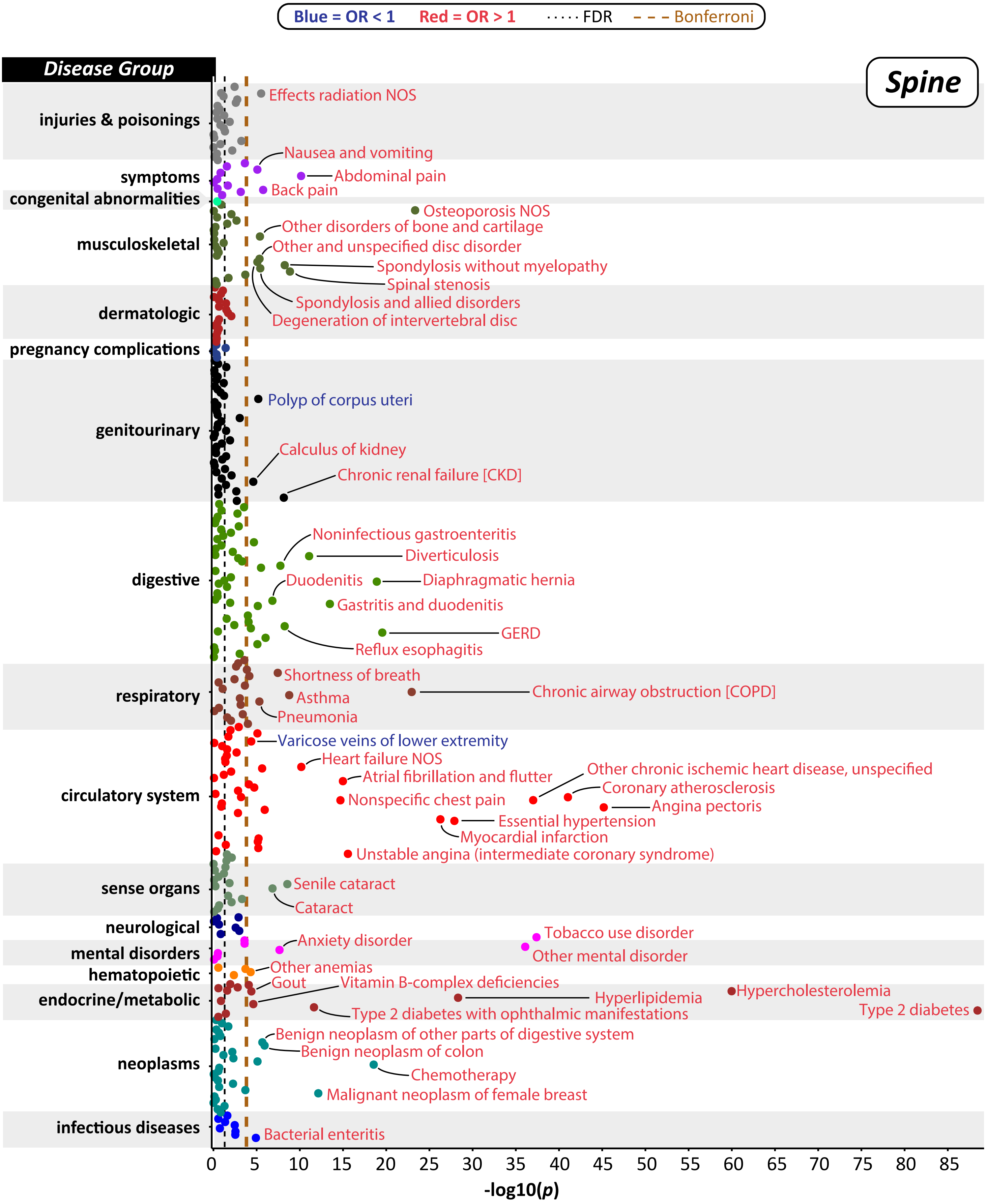

**Supplementary Figure 5 –** Manhattan plot of the Obs-PheWAS (sensitivity analysis: incident+prevalent) results for spine BMFF (n=48,427).

The y axes represent phenotypes (aggregated on International Classification of Disease codes), and the x axes represent the −log10 p values of two-sided test for logistic regression between BMFF and each of the phenotypes. Each dot represents one phenotype, and the colours indicate their according categories. Diseases in red have an OR >1 and those in blue have OR <1. The dotted orange line indicates a Bonferroni-correction and the dotted black line indicates an FDR-q correction. A total of 73 and 142 significant phenotypes passed Bonferroni-correction and FDR-q correction respectively; because of space limitations, not all significant disease names could be shown on the figure [see Supplementary Data 5 for further details].

# Supplementary Figure 6

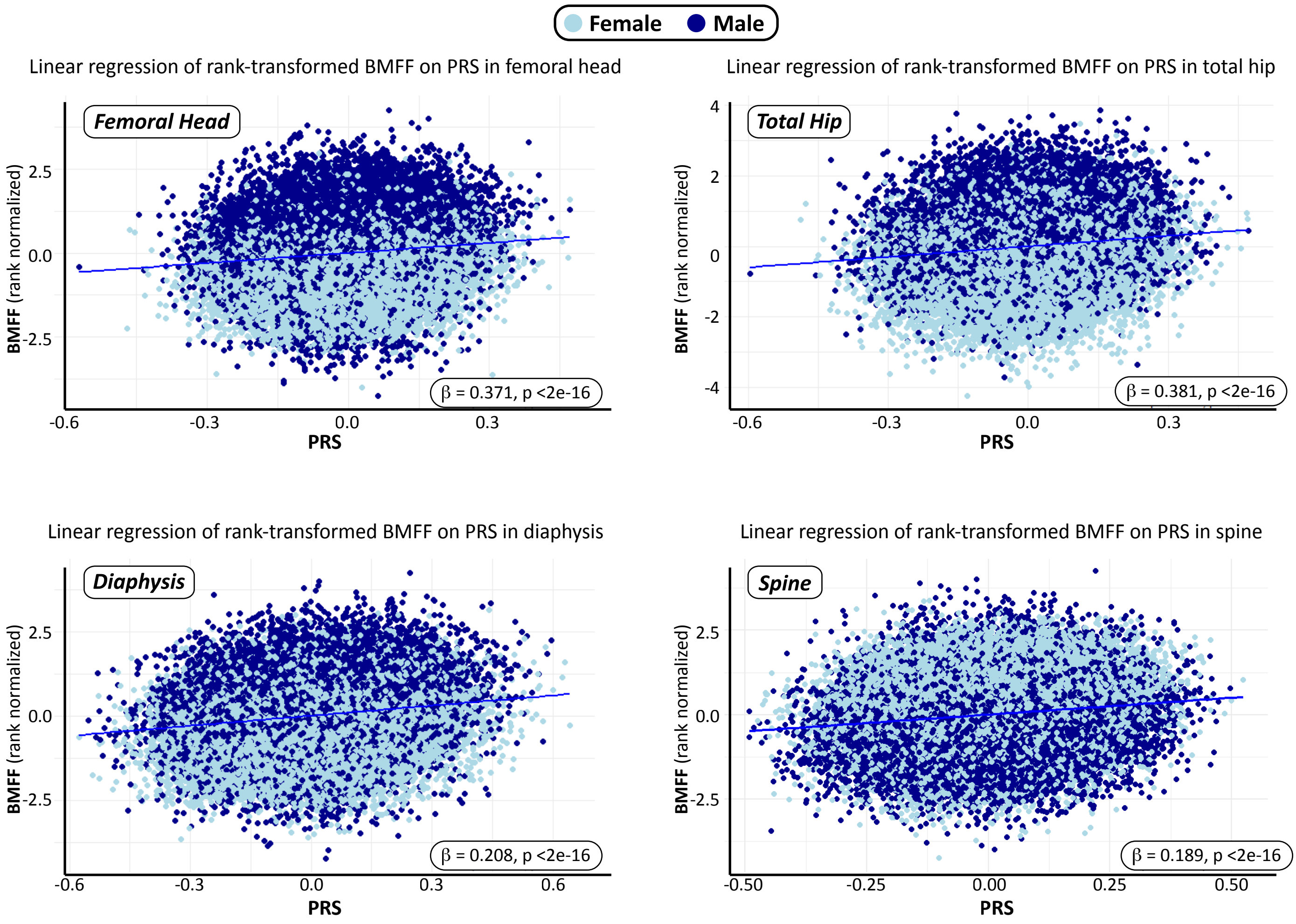


**Supplementary Figure 6 –** Linear regression of BMFF and PRS.

Scatter plots display the linear regression analysis between rank-transformed bone marrow fat fraction (BMFF) and polygenic risk scores (PRS) across four bone regions. Each dot represents a participant, with sex-stratified coloring (light blue for females, dark blue for males). The fitted regression lines indicate the association between PRS and BMFF, with β coefficients and p-values displayed on each plot. A positive β coefficient suggests a direct association between higher PRS and increased BMFF.

# Supplementary Figure 7

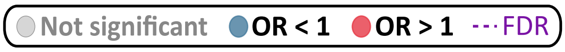


**A**

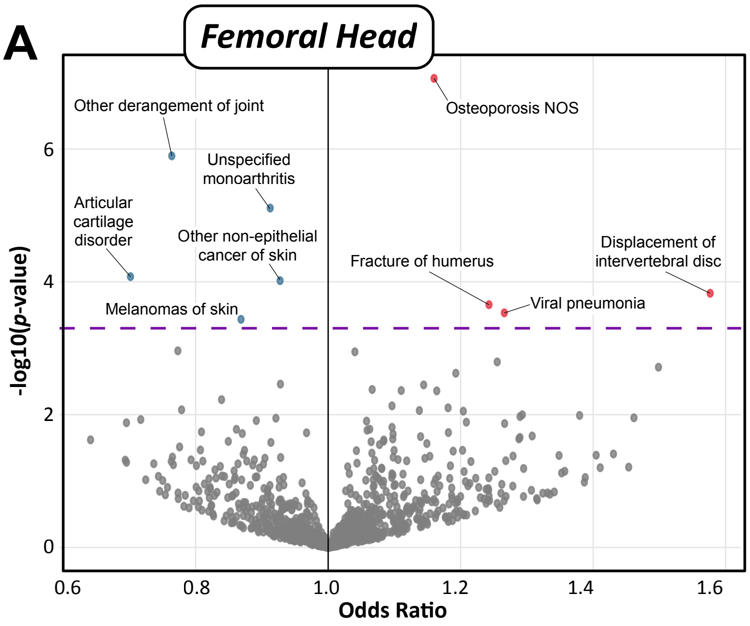


| Disease | n (cases) | OR (95% CI) |
|---|---|---|
| Osteoporosis NOS | 12457 | 1.16 (1.10-1.23) |
| Other derangement of joint | 2806 | 0.76 (0.68-0.85) |
| Unspecified monoarthritis | 23348 | 0.91 (0.88-0.95) |
| Articular cartilage disorder | 1057 | 0.70 (0.59-0.84) |
| Other non-epithelial cancer of skin | 26248 | 0.93 (0.89-0.96) |
| Displacement of intervertebral disc | 618 | 1.58 (1.25-2.00) |
| Fracture of humerus | 2566 | 1.24 (1.11-1.40) |
| Viral pneumonia | 2100 | 1.27 (1.11-1.44) |
| Melanomas of skin | 5637 | 0.87 (0.80-0.94) |

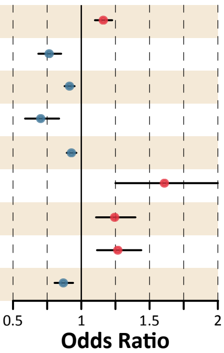


**B**

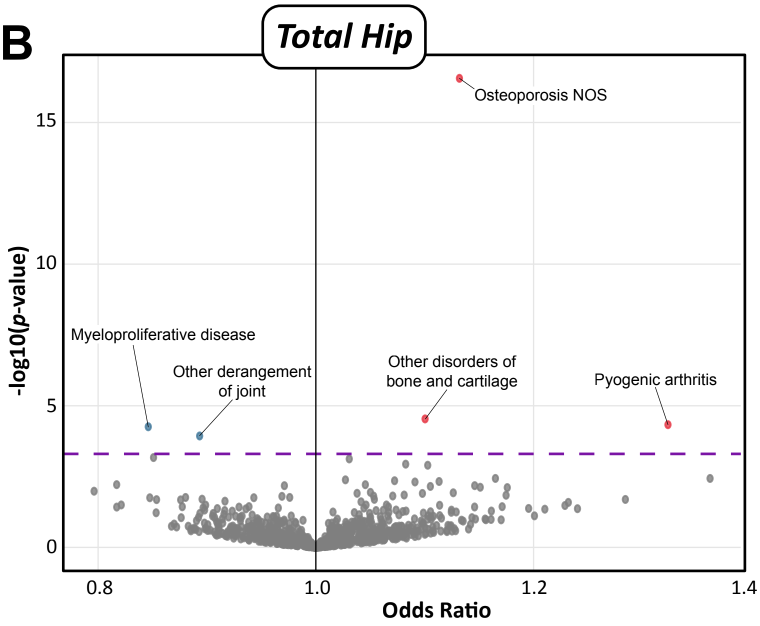


| Disease | n (cases) | OR (95% CI) |
|---|---|---|
| Osteoporosis NOS | 12423 | 1.13 (1.10-1.16) |
| Other disorders of bone and cartilage | 4785 | 1.10 (1.05-1.15) |
| Pyogenic arthritis | 524 | 1.33 (1.16-1.51) |
| Myeloproliferative disease | 1395 | 0.84 (0.78-0.92) |
| Other derangement of joint | 2821 | 0.89 (0.84-0.95) |

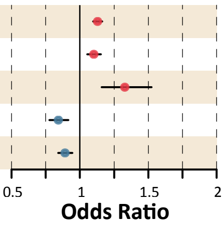


**C**

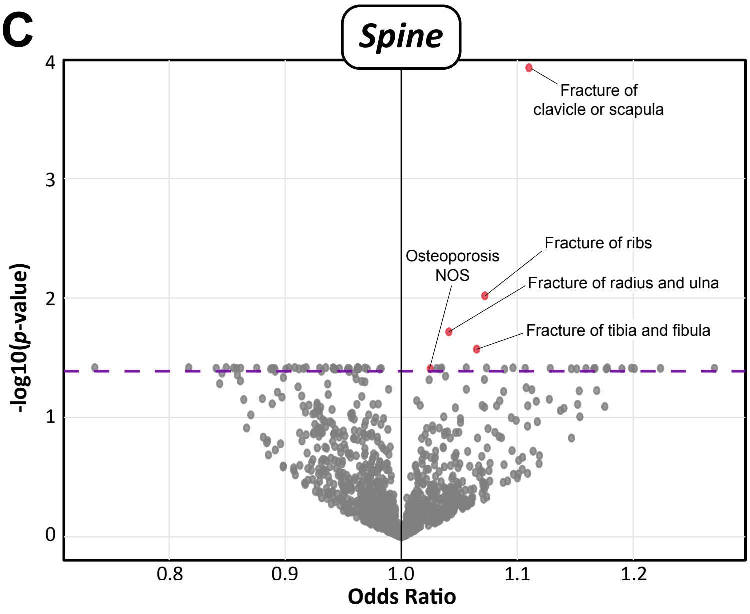


| Disease | n (cases) | OR (95% CI) |
|---|---|---|
| Fracture of clavicle or scapula | 2685 | 1.11 (1.05-1.17) |
| Fracture of ribs | 2726 | 1.07 (1.02-1.13) |
| Fracture of radius and ulna | 6688 | 1.04 (1.01-1.08) |
| Fracture of tibia and fibula | 2417 | 1.06 (1.01-1.13) |
| Osteoporosis NOS | 12416 | 1.02 (1.00-1.05) |

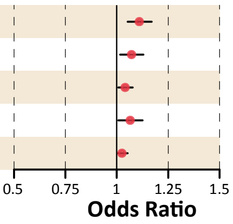

**Supplementary Figure** 7 **–** PRS1-PheWAS significant PheCodes.

Volcano plots show odds ratios (OR) and -log10(p) values (two-sided) for PheCODE diseases associated with femoral head PRS1 (A), total hip PRS1 (B) and spine PRS1 (C); data for diaphysis PRS1 are shown in Figure 3 of the main text. Each dot represents a disease, with the x-axis indicating the OR and the y-axis showing the -log10(p). Labels of disease names are shown for all significant diseases (FDR < 0.05)*;* significant associations with OR<1 are shown as blue dots; those with OR>1 are red dots; and non-significant associations are shown as grey dots. For each region, the dashed horizontal line indicates the FDR threshold. Forest plots beneath each volcano plot are included to show each significant PheCODE disease's OR with 95% CIs.

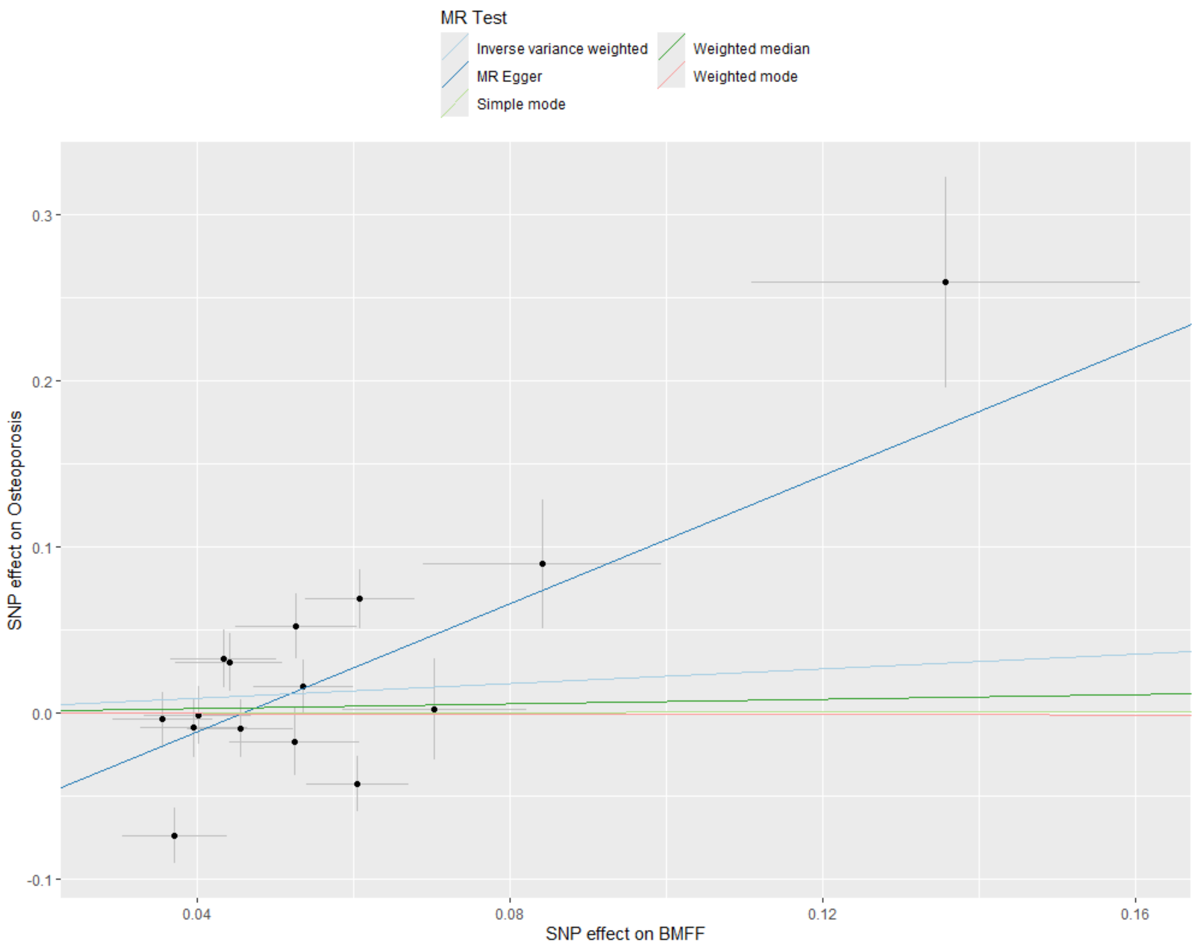


**Supplementary Figure 8 –** MR-scatter plot for femoral head BMFF-Osteoporosis.

Each dot represents a SNP (clump r2<0.001), with the x-axis showing the SNP effect on BMFF and the y-axis showing the SNP effect on osteoporosis. The fitted lines represent different MR estimation methods, including: inverse variance weighted (IVW; (light blue), MR Egger (dark blue), weighted mode (red), simple mode (light green), weighted median (dark green).

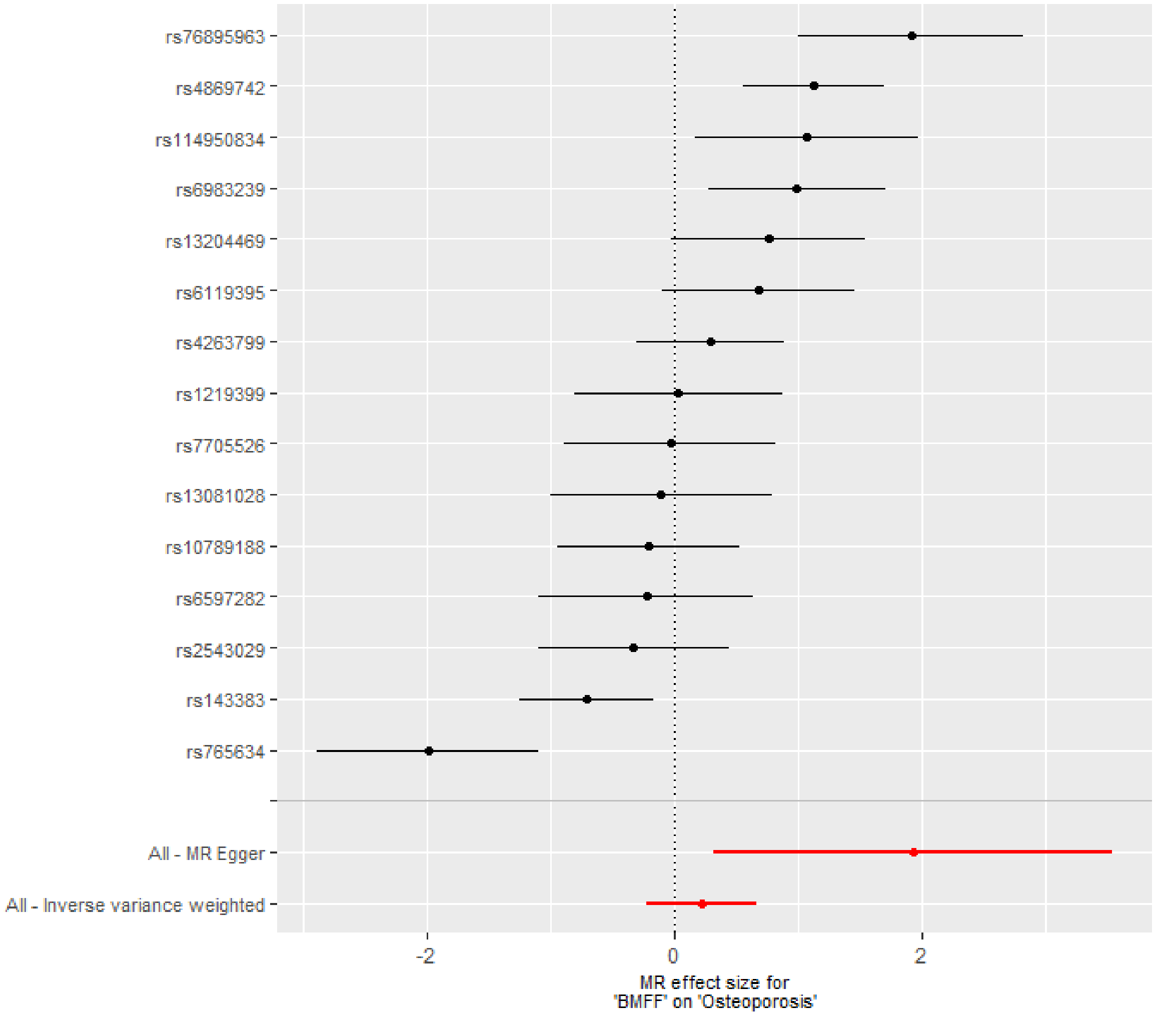


**Supplementary Figure 9 –** MR-forest plot for femoral head BMFF-Osteoporosis.

The x-axis represents the effect size with the corresponding 95% CIs. Each black point represents the log OR for osteoporosis per SD increase in BMFF, produced using each of the 'BMFF SNPs' (clump $r2<0.001$) as separate instruments, and red points showing the combined causal estimate using all SNPs together in a single instrument, using IVW random effects and MR-Egger. Horizontal lines denote 95% CI.

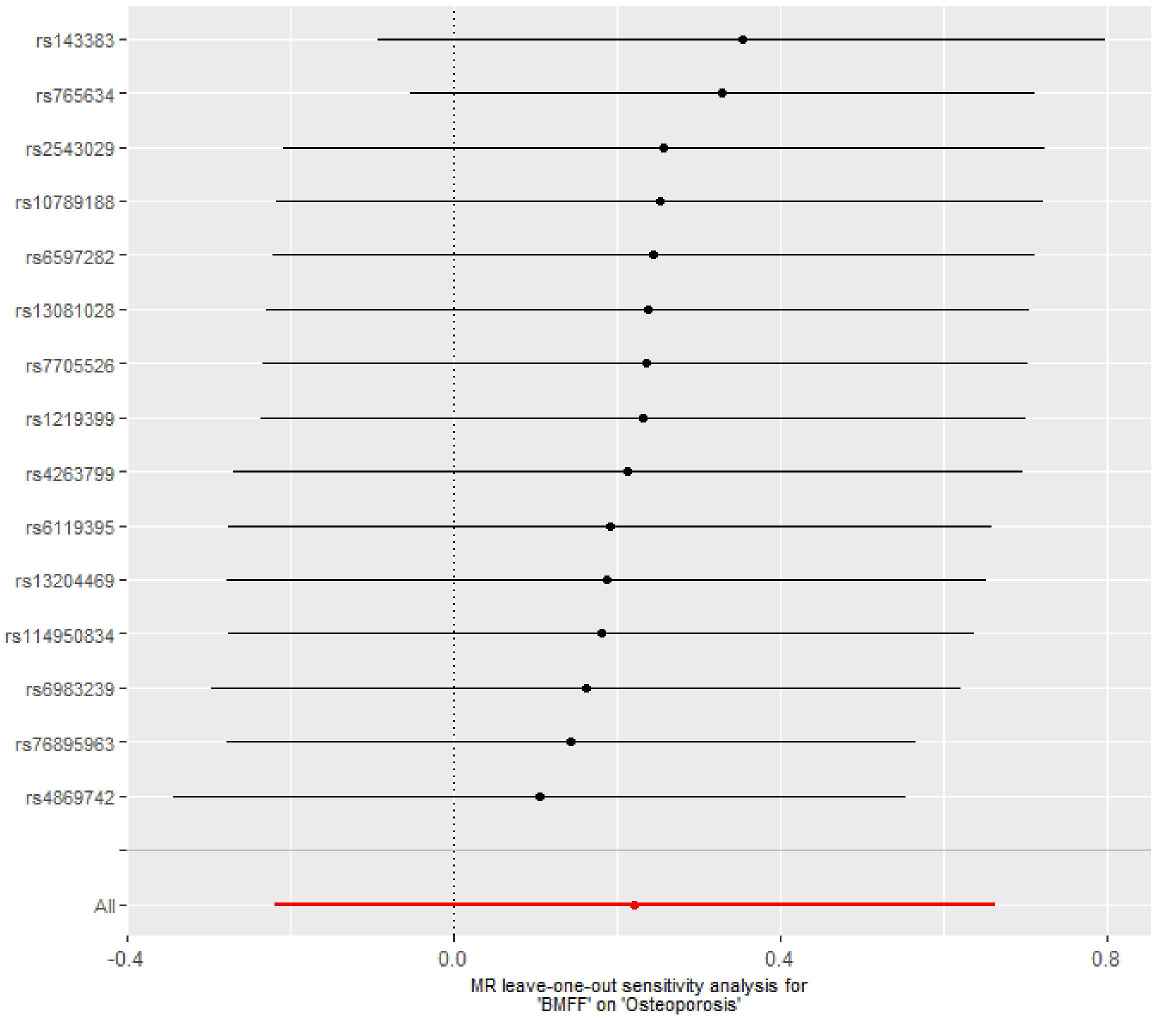


**Supplementary Figure 10 –** Forest plot of leave-one-out sensitivity results for femoral head BMFF-Osteoporosis.

Each black point represents the IVW MR method applied to estimate the causal effect of BMFF on osteoporosis excluding that particular variant from the analysis. The red point depicts the IVW estimate using all SNPs. There are no instances where the exclusion of one particular SNP leads to dramatic changes in the overall result.

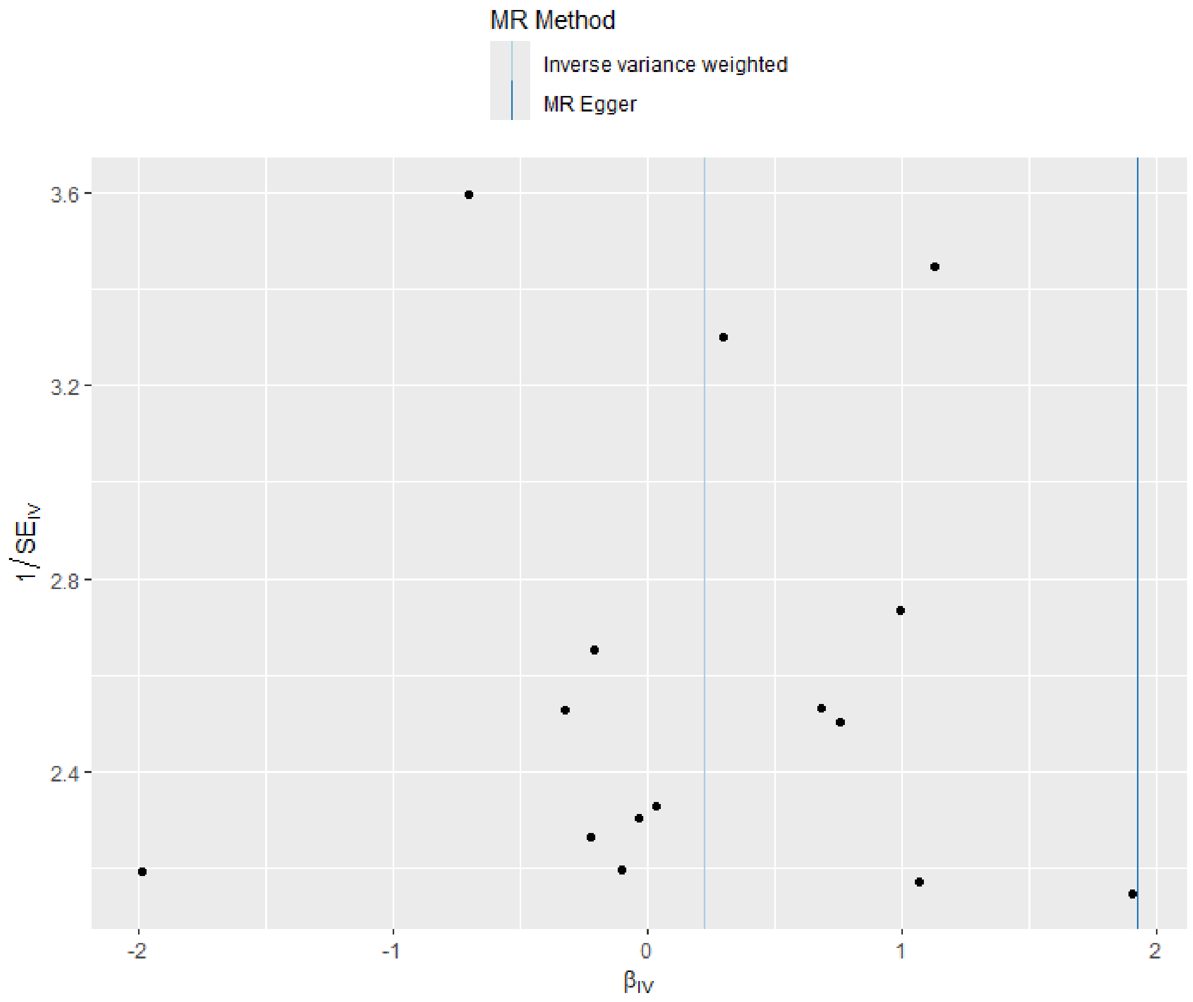


**Supplementary Figure 11 –** MR-funnel plot of femoral head BMFF-Osteoporosis.

Vertical lines show the causal estimates using all SNPs combined into a single instrument for IVW random effects and MR-Egger methods.

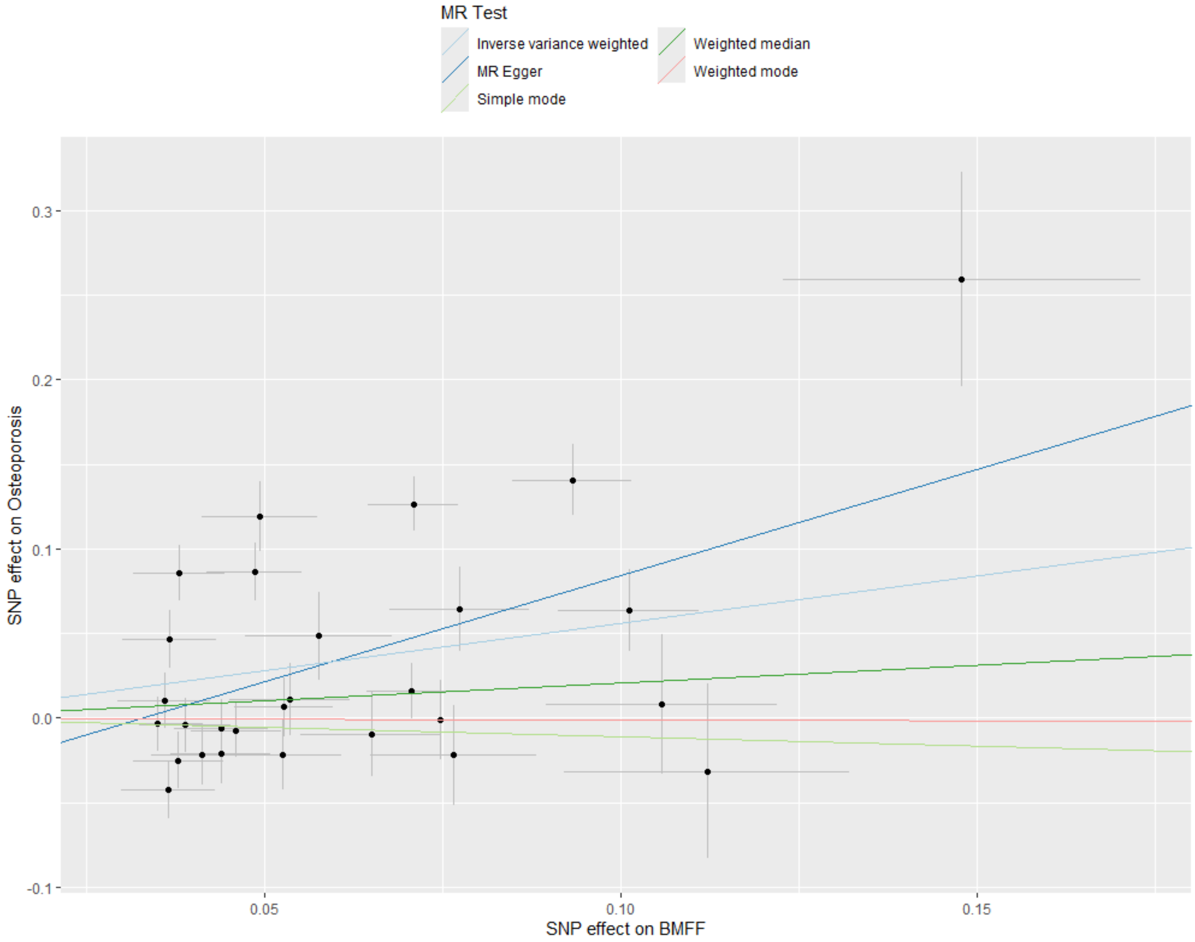


**Supplementary Figure 12 –** MR-scatter plot for total hip BMFF-Osteoporosis.

Each dot represents a SNP (clump r2<0.001), with the x-axis showing the SNP effect on BMFF and the y-axis showing the SNP effect on osteoporosis. The fitted lines represent different MR estimation methods, including: inverse variance weighted (IVW; (light blue), MR Egger (dark blue), weighted mode (red), simple mode (light green), weighted median (dark green).

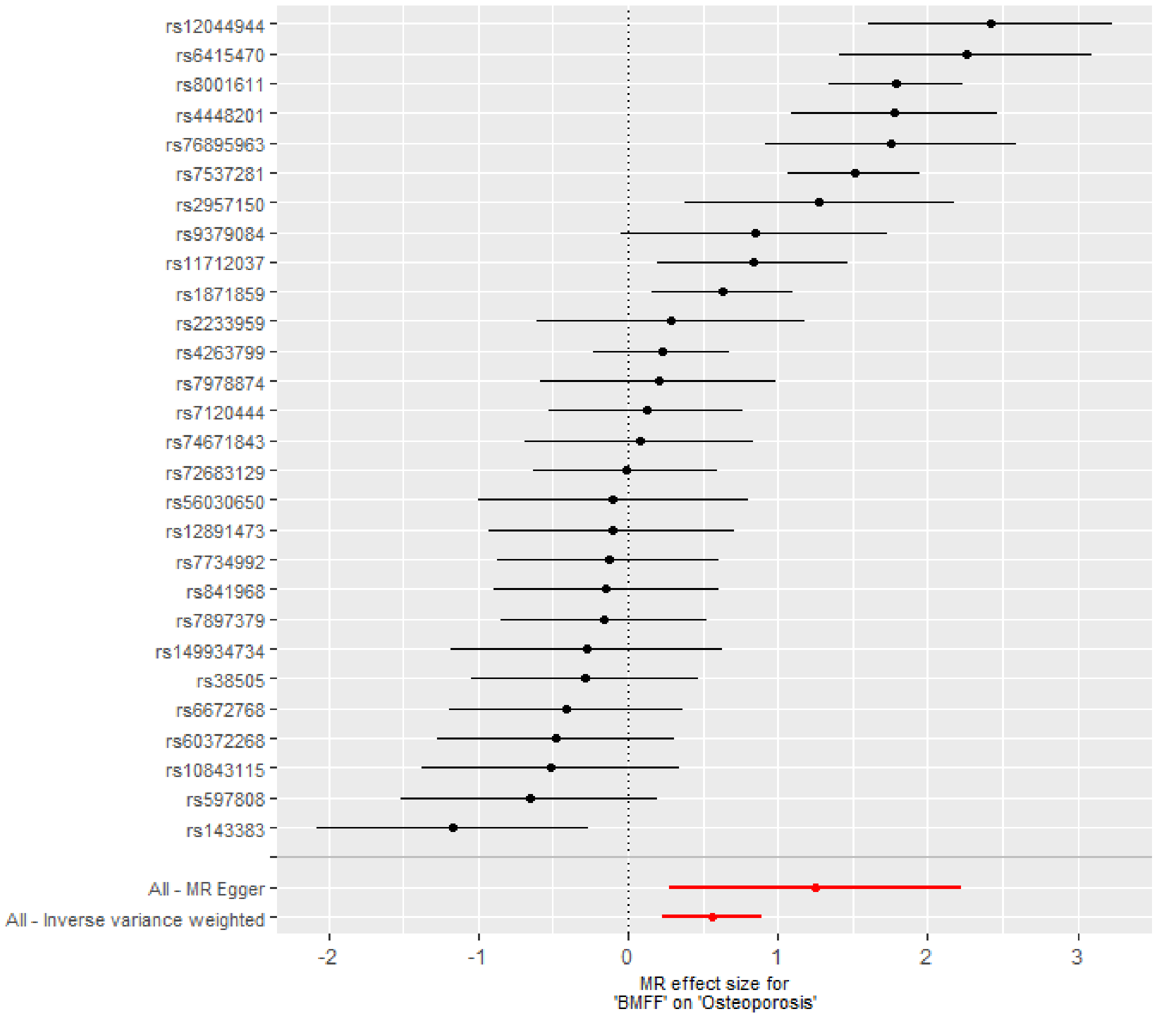


**Supplementary Figure 13 –** MR-forest plot for total hip BMFF-Osteoporosis.

The x-axis represents the effect size with the corresponding 95% CIs. Each black point represents the log OR for osteoporosis per SD increase in BMFF, produced using each of the 'BMFF SNPs' (clump r2<0.001) as separate instruments, and red points showing the combined causal estimate using all SNPs together in a single instrument, using IVW random effects and MR-Egger. Horizontal lines denote 95% CI.

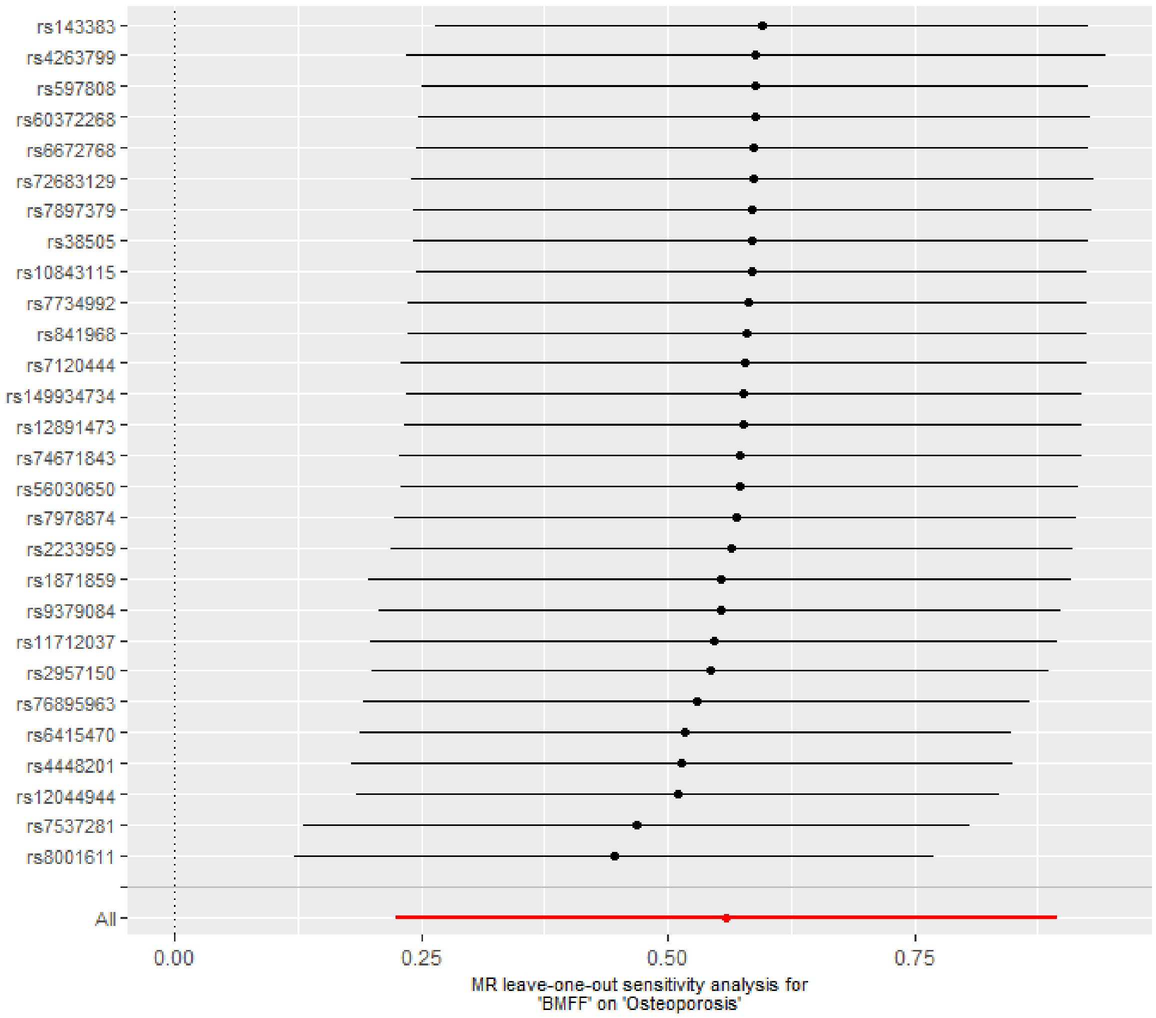


**Supplementary Figure 14 –** Forest plot of leave-one-out sensitivity results for total hip BMFF-Osteoporosis.

Each black point represents the IVW MR method applied to estimate the causal effect of BMFF on osteoporosis excluding that particular variant from the analysis. The red point depicts the IVW estimate using all SNPs. There are no instances where the exclusion of one particular SNP leads to dramatic changes in the overall result.

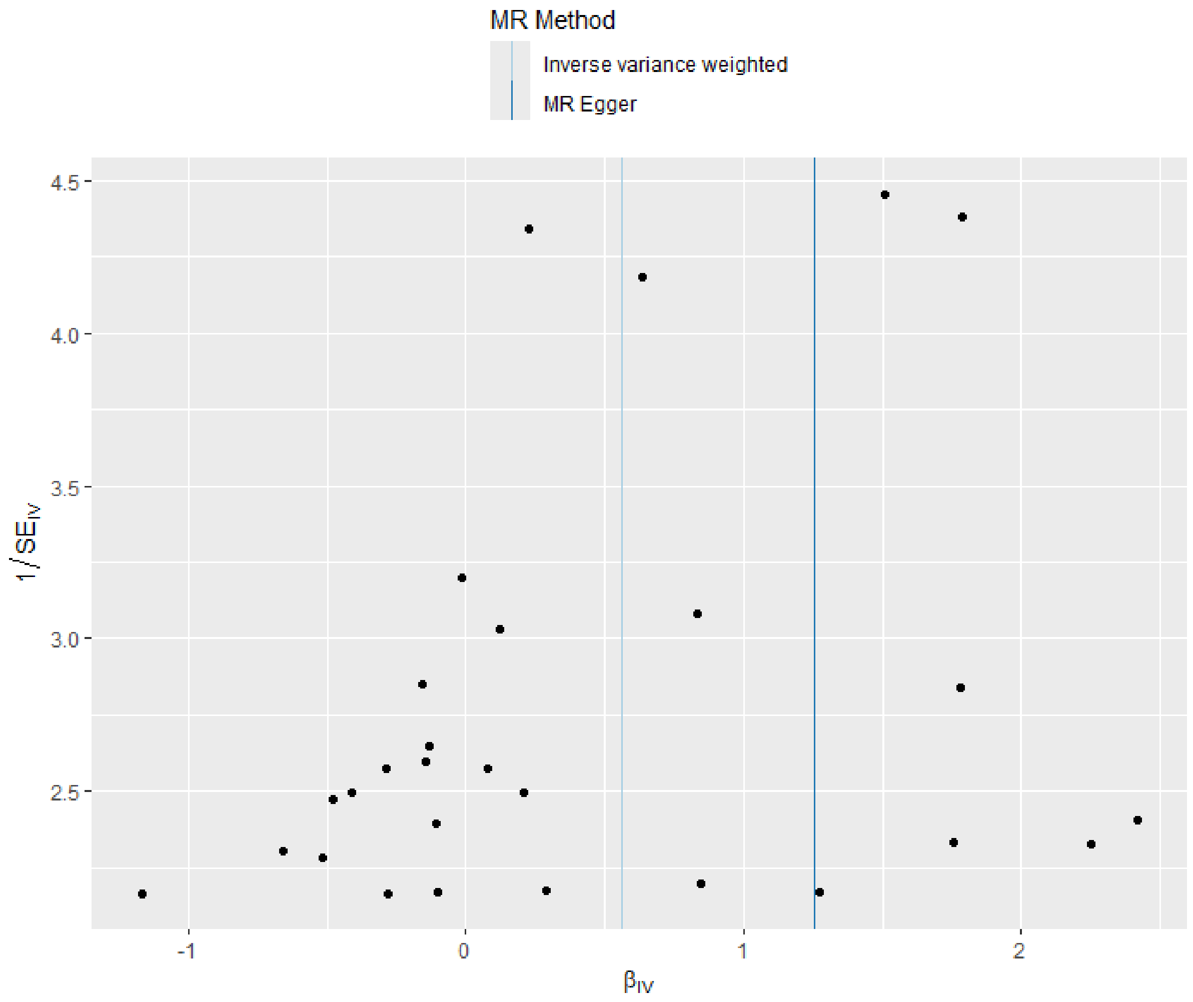


**Supplementary Figure 15 –** MR-funnel plot of total hip BMFF-Osteoporosis.

Vertical lines show the causal estimates using all SNPs combined into a single instrument for IVW random effects and MR-Egger methods.

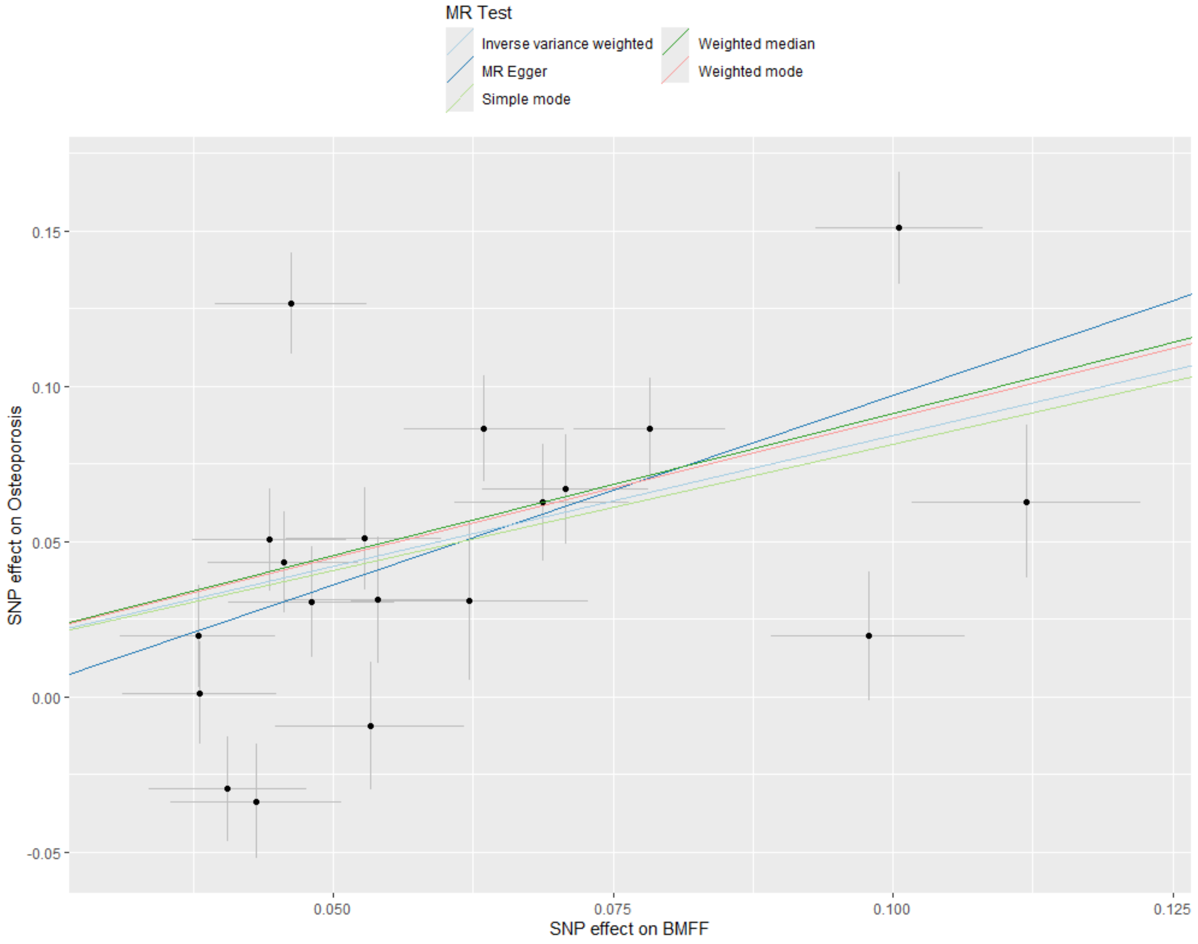


**Supplementary Figure 16 –** MR-scatter plot for diaphysis BMFF-Osteoporosis.

Each dot represents a SNP (clump r2<0.001), with the x-axis showing the SNP effect on BMFF and the y-axis showing the SNP effect on osteoporosis. The fitted lines represent different MR estimation methods, including: inverse variance weighted (IVW; (light blue), MR Egger (dark blue), weighted mode (red), simple mode (light green), weighted median (dark green).

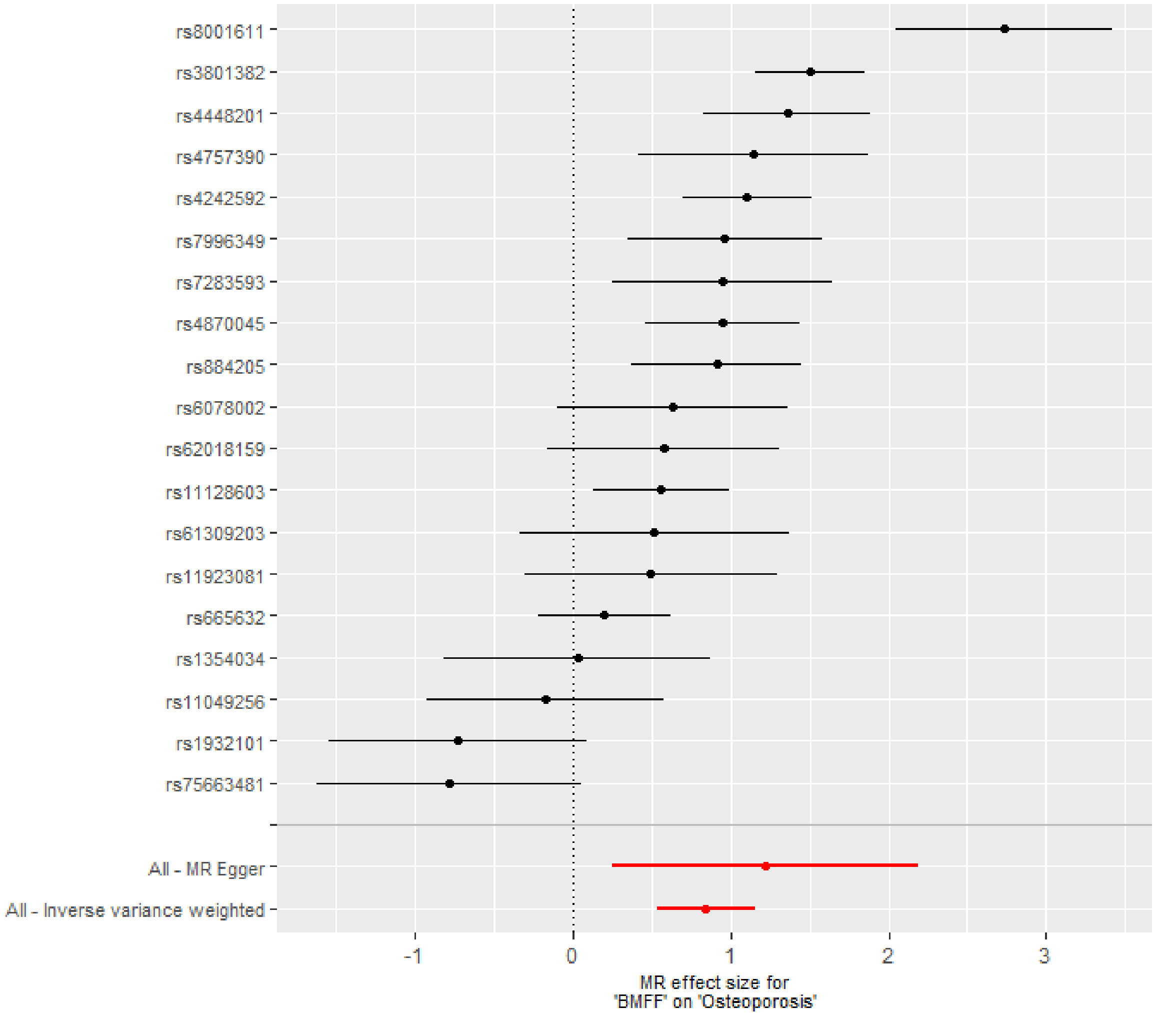


**Supplementary Figure 17 –** MR-forest plot for diaphysis BMFF-Osteoporosis.

The x-axis represents the effect size with the corresponding 95% CIs. Each black point represents the log OR for osteoporosis per SD increase in BMFF, produced using each of the 'BMFF SNPs' (clump r2<0.001) as separate instruments, and red points showing the combined causal estimate using all SNPs together in a single instrument, using IVW random effects and MR-Egger. Horizontal lines denote 95% CI.

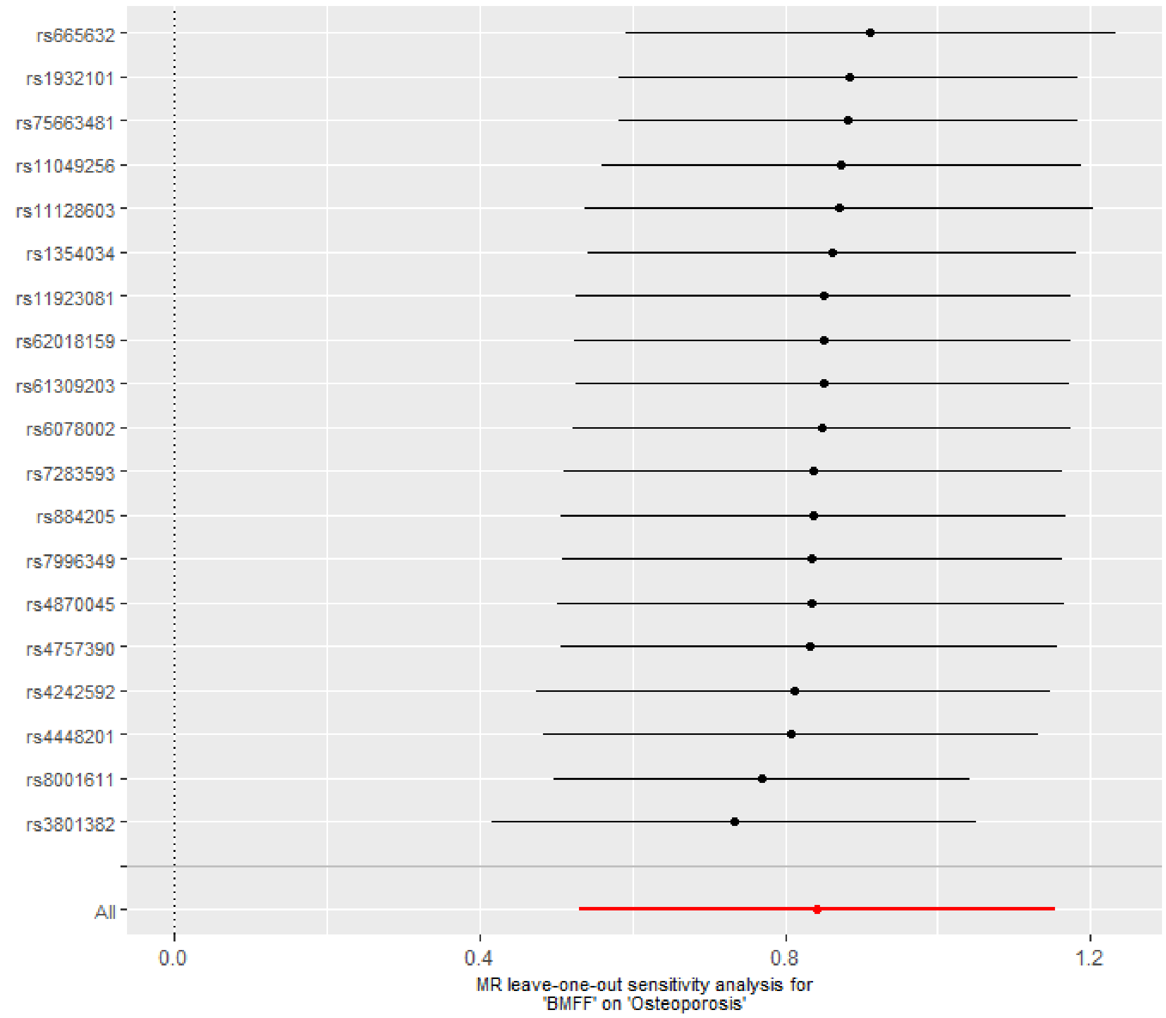


**Supplementary Figure 18 –** Forest plot of leave-one-out sensitivity results for diaphysis BMFF-Osteoporosis.

Each black point represents the IVW MR method applied to estimate the causal effect of BMFF on osteoporosis excluding that particular variant from the analysis. The red point depicts the IVW estimate using all SNPs. There are no instances where the exclusion of one particular SNP leads to dramatic changes in the overall result.

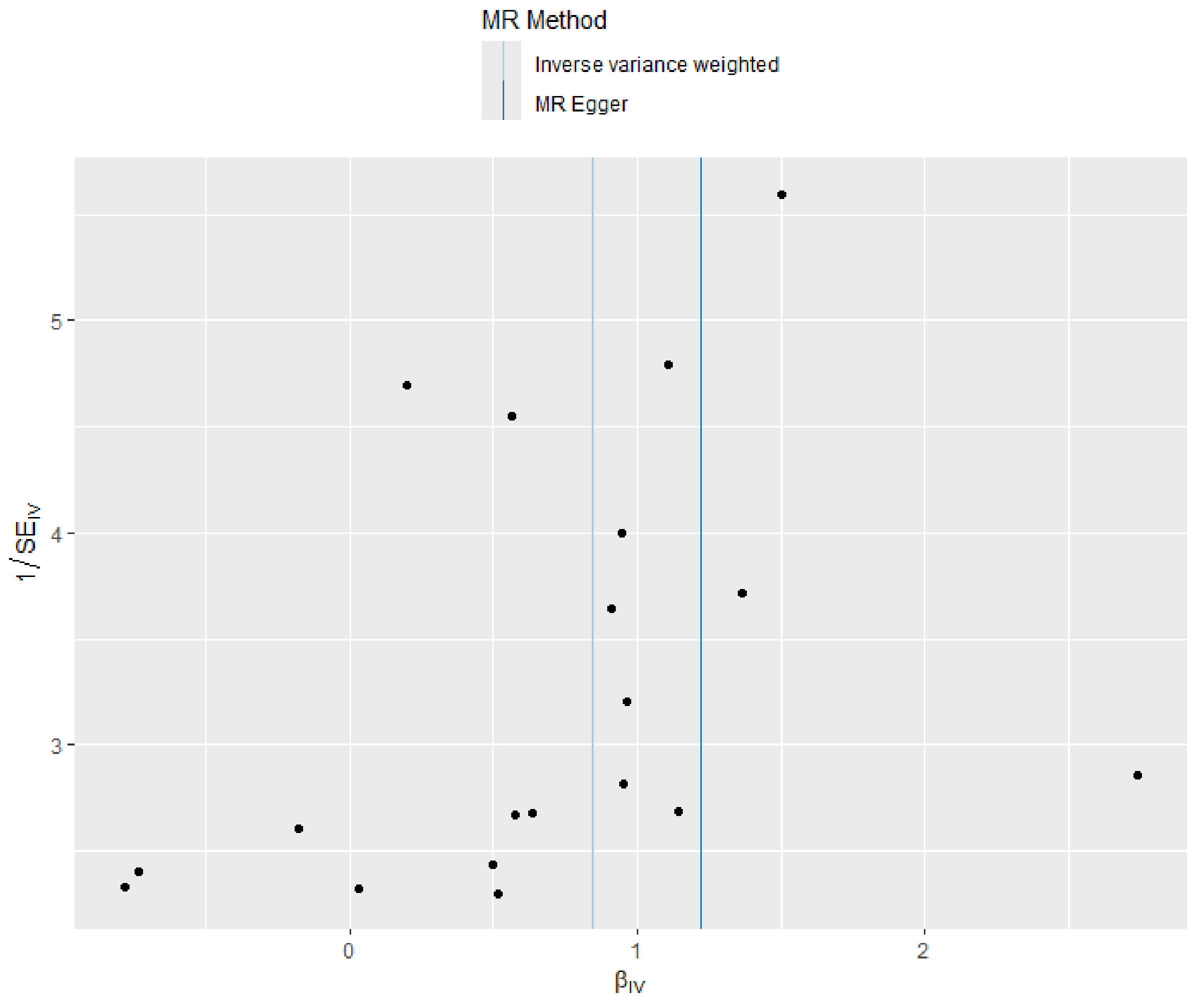


**Supplementary Figure 19 –** MR-funnel plot of diaphysis BMFF-Osteoporosis.

Vertical lines show the causal estimates using all SNPs combined into a single instrument for IVW random effects and MR-Egger methods.

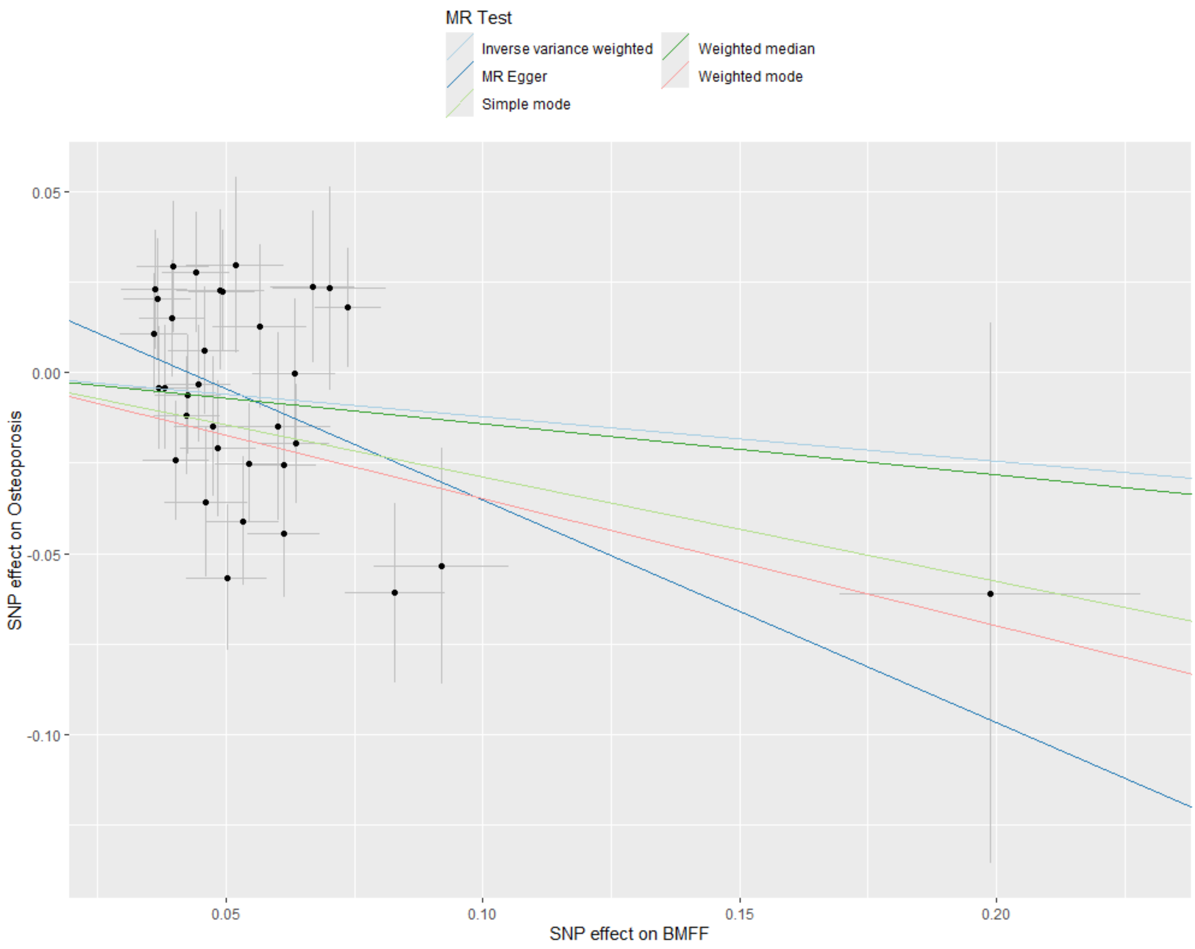


**Supplementary Figure 20 –** MR-scatter plot for spine BMFF-Osteoporosis.

Each dot represents a SNP (clump r2<0.001), with the x-axis showing the SNP effect on BMFF and the y-axis showing the SNP effect on osteoporosis. The fitted lines represent different MR estimation methods, including: inverse variance weighted (IVW; (light blue), MR Egger (dark blue), weighted mode (red), simple mode (light green), weighted median (dark green).

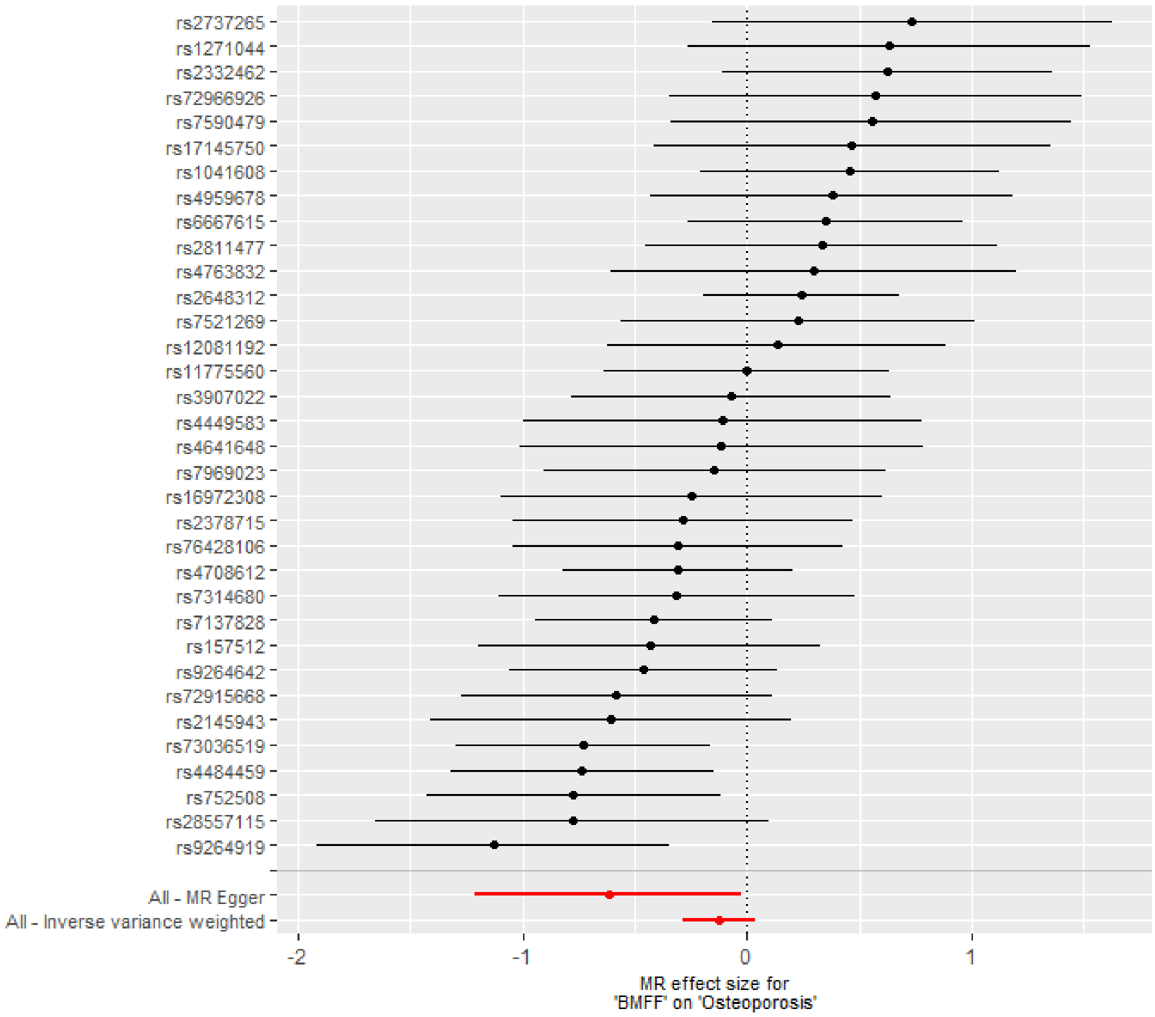


**Supplementary Figure 21 –** MR-forest plot for spine BMFF-Osteoporosis.

The x-axis represents the effect size with the corresponding 95% CIs. Each black point represents the log OR for osteoporosis per SD increase in BMFF, produced using each of the 'BMFF SNPs' (clump r2<0.001) as separate instruments, and red points showing the combined causal estimate using all SNPs together in a single instrument, using IVW random effects and MR-Egger. Horizontal lines denote 95% CI.

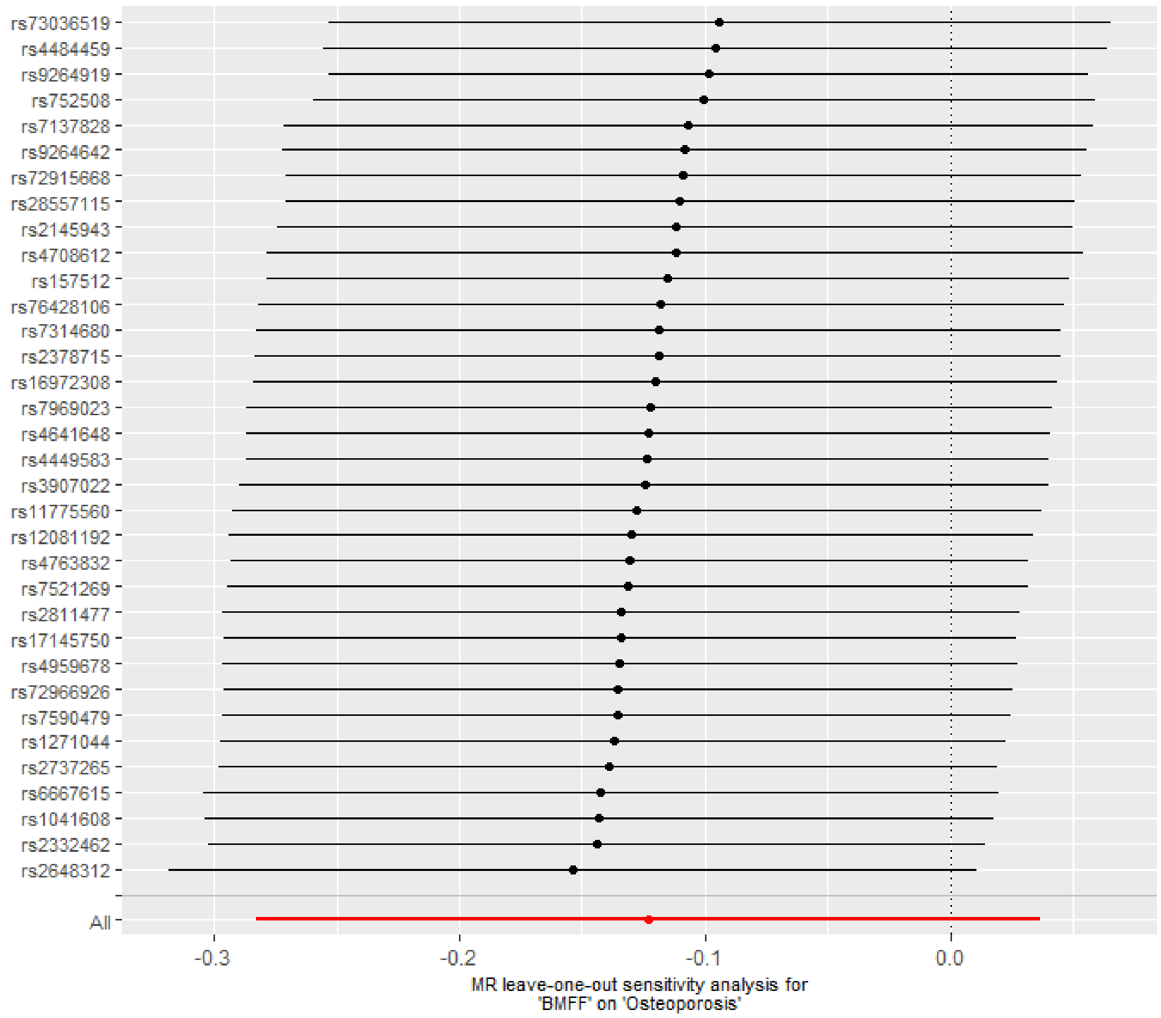


**Supplementary Figure 22 –** Forest plot of leave-one-out sensitivity results for spine BMFF-Osteoporosis.

Each black point represents the IVW MR method applied to estimate the causal effect of BMFF on osteoporosis excluding that particular variant from the analysis. The red point depicts the IVW estimate using all SNPs. There are no instances where the exclusion of one particular SNP leads to dramatic changes in the overall result.

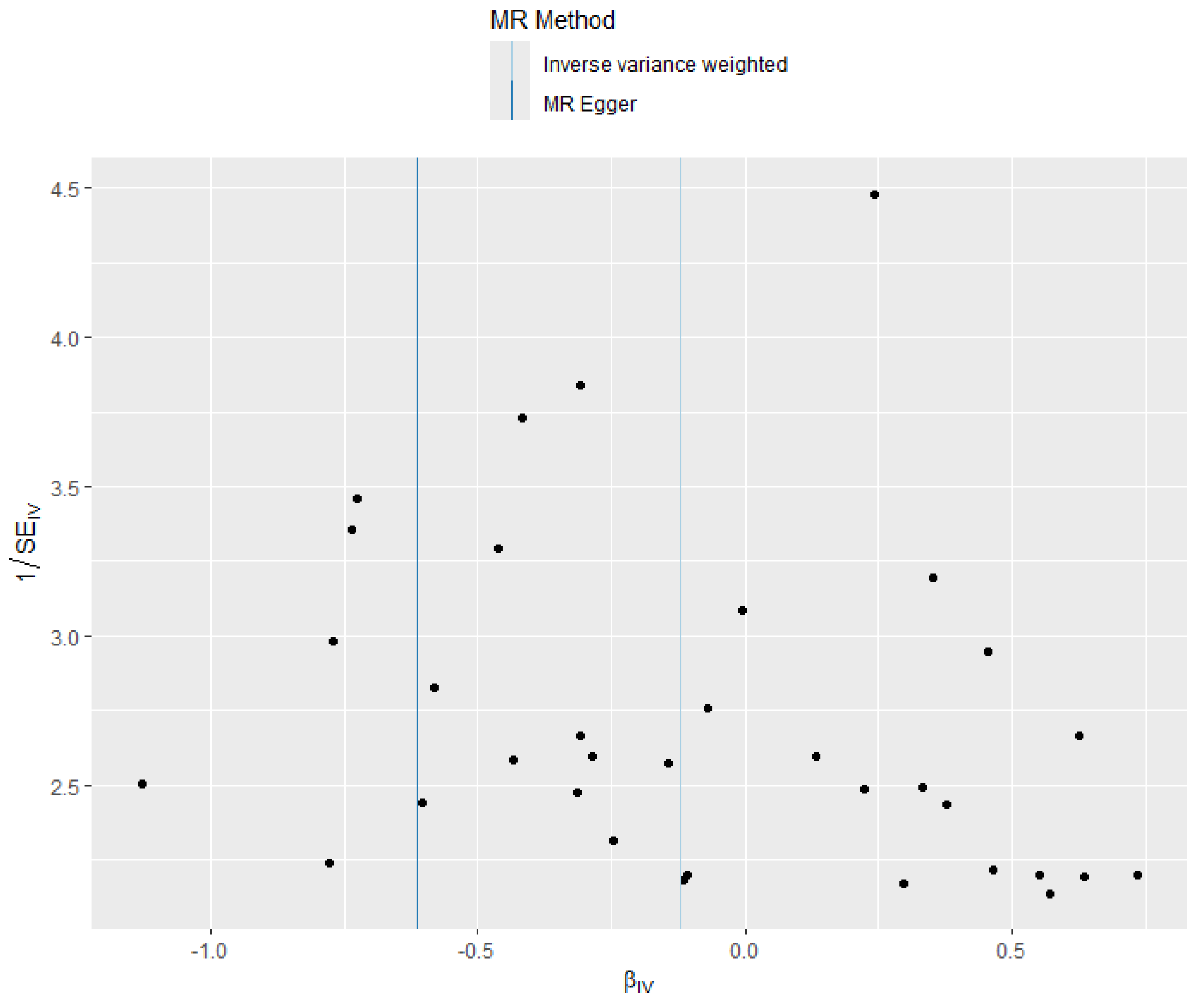


**Supplementary Figure 23 –** MR-funnel plot of spine BMFF-Osteoporosis.

Vertical lines show the causal estimates using all SNPs combined into a single instrument for IVW random effects and MR-Egger methods.

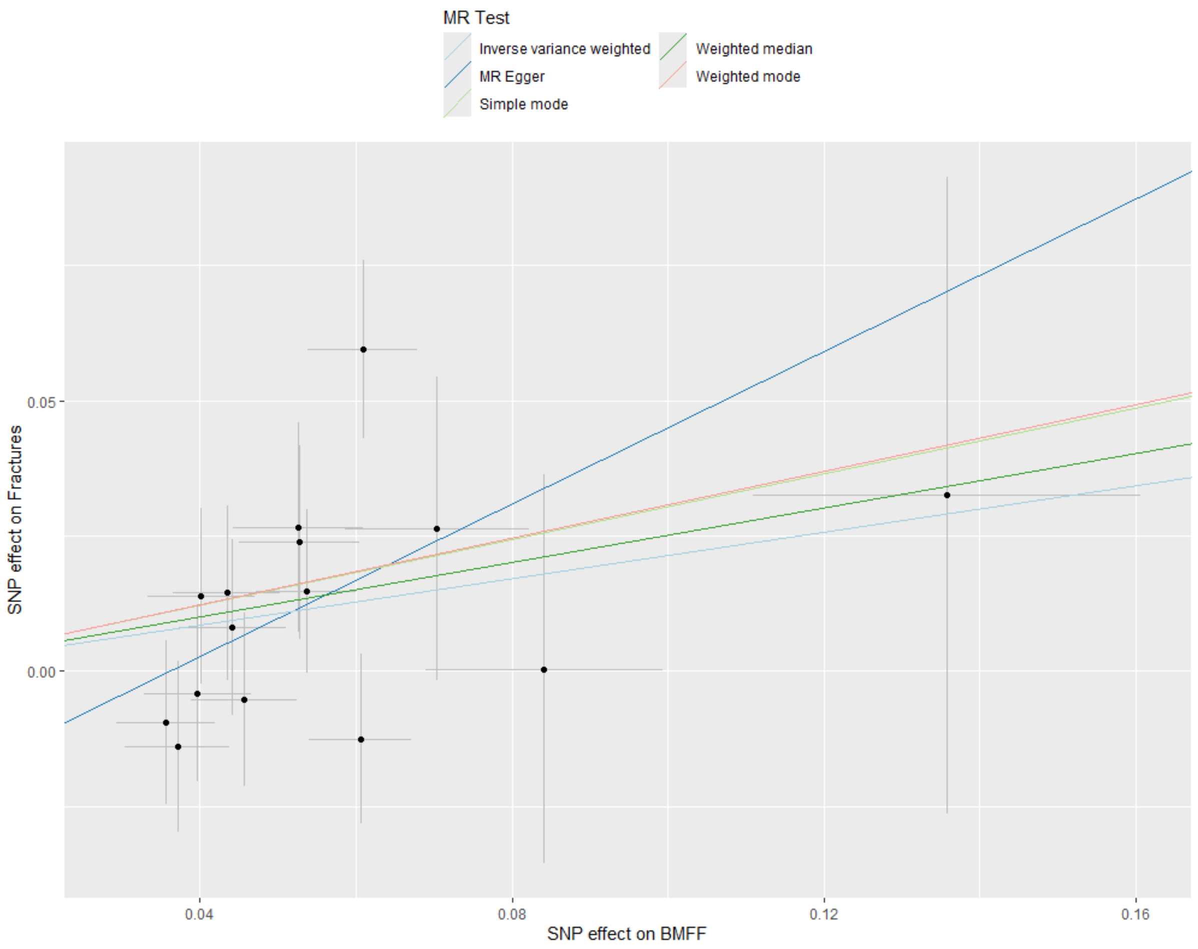


**Supplementary Figure 24 –** MR-scatter plot for femoral head BMFF-Fractures.

Each dot represents a SNP (clump r2<0.001), with the x-axis showing the SNP effect on BMFF and the y-axis showing the SNP effect on fractures. The fitted lines represent different MR estimation methods, including: inverse variance weighted (IVW; (light blue), MR Egger (dark blue), weighted mode (red), simple mode (light green), weighted median (dark green).

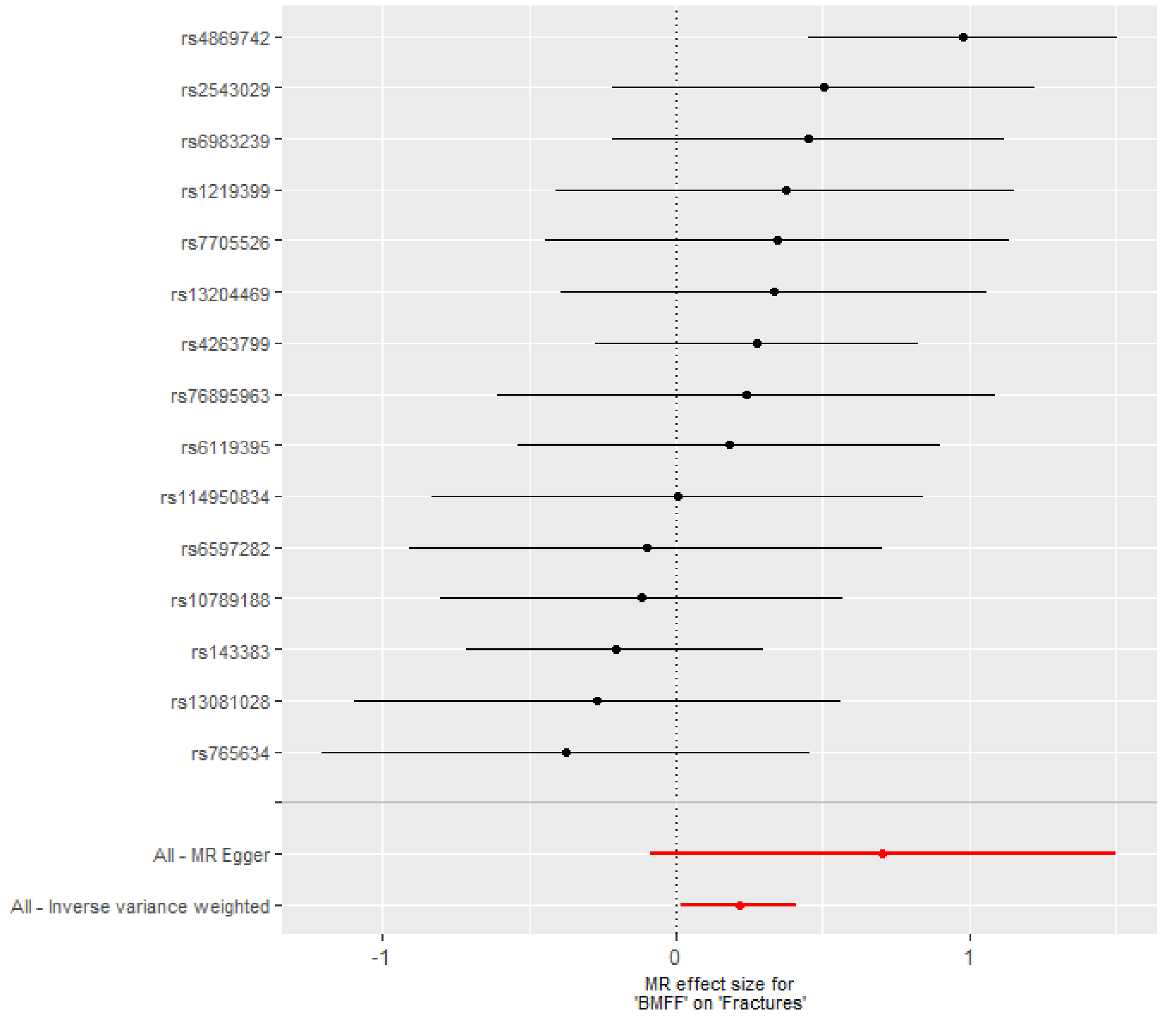


**Supplementary Figure 25 –** MR-forest plot for femoral head BMFF-Fractures.

The x-axis represents the effect size with the corresponding 95% CIs. Each black point represents the log OR for fractures per SD increase in BMFF, produced using each of the 'BMFF SNPs' (clump r2<0.001) as separate instruments, and red points showing the combined causal estimate using all SNPs together in a single instrument, using IVW random effects and MR-Egger. Horizontal lines denote 95% CI.

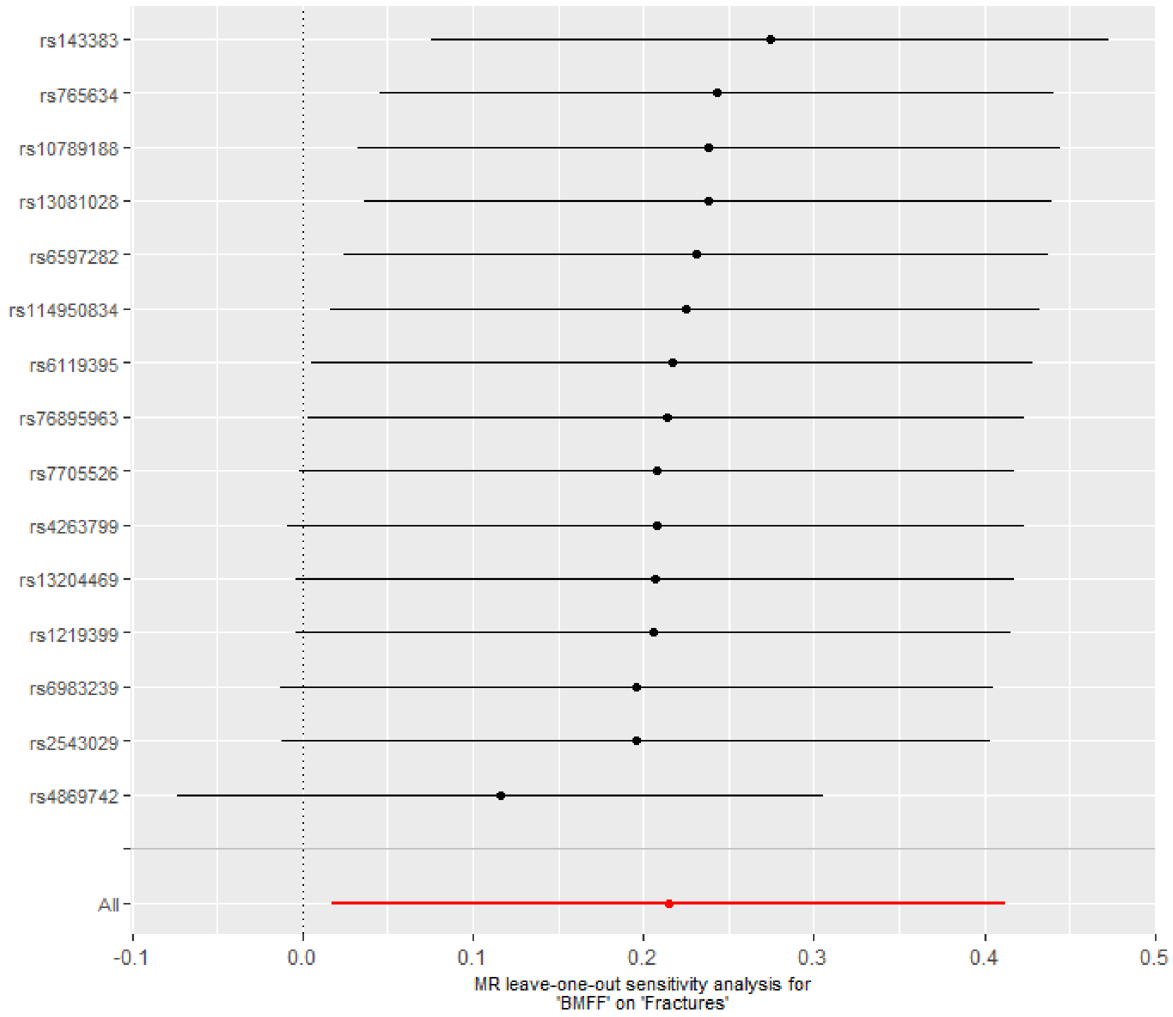


**Supplementary Figure 26 –** Forest plot of leave-one-out sensitivity results for femoral head BMFF-Fractures.

Each black point represents the IVW MR method applied to estimate the causal effect of BMFF on fractures excluding that particular variant from the analysis. The red point depicts the IVW estimate using all SNPs. There are no instances where the exclusion of one particular SNP leads to dramatic changes in the overall result.

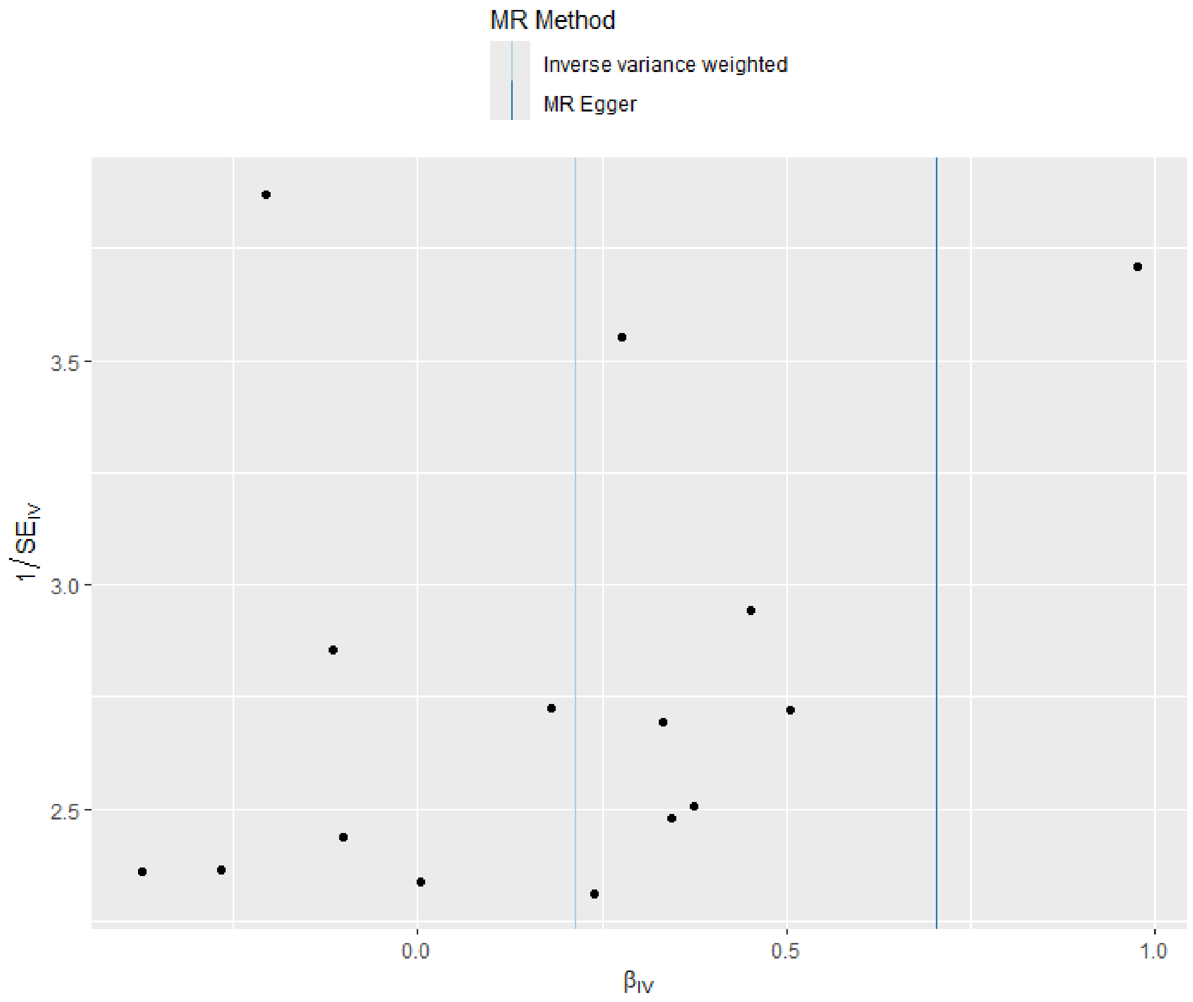


**Supplementary Figure 27 –** MR-funnel plot of femoral head BMFF-Fractures.

Vertical lines show the causal estimates using all SNPs combined into a single instrument for IVW random effects and MR-Egger methods.

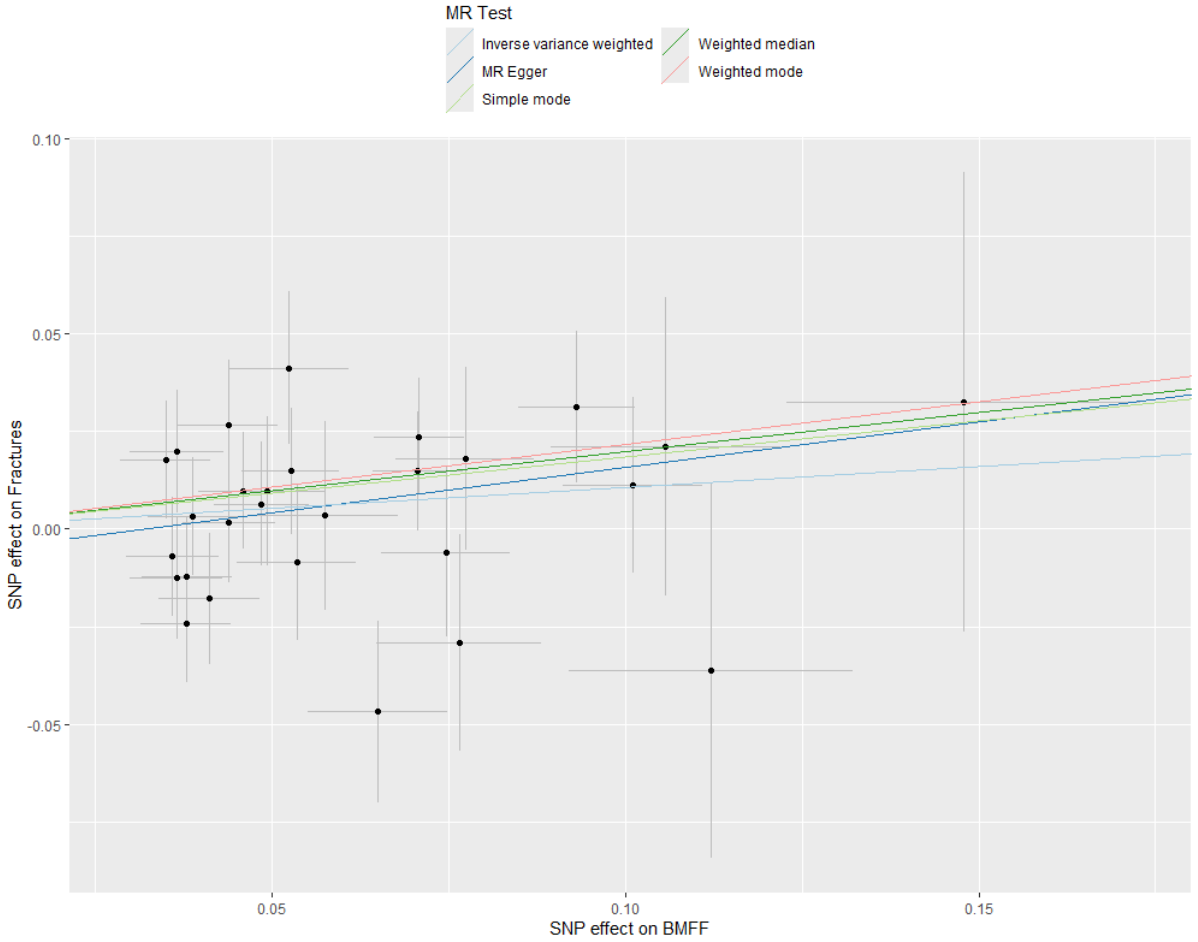


**Supplementary Figure 28 –** MR-scatter plot for total hip BMFF-Fractures.

Each dot represents a SNP (clump r2<0.001), with the x-axis showing the SNP effect on BMFF and the y-axis showing the SNP effect on fractures. The fitted lines represent different MR estimation methods, including: inverse variance weighted (IVW; (light blue), MR Egger (dark blue), weighted mode (red), simple mode (light green), weighted median (dark green).

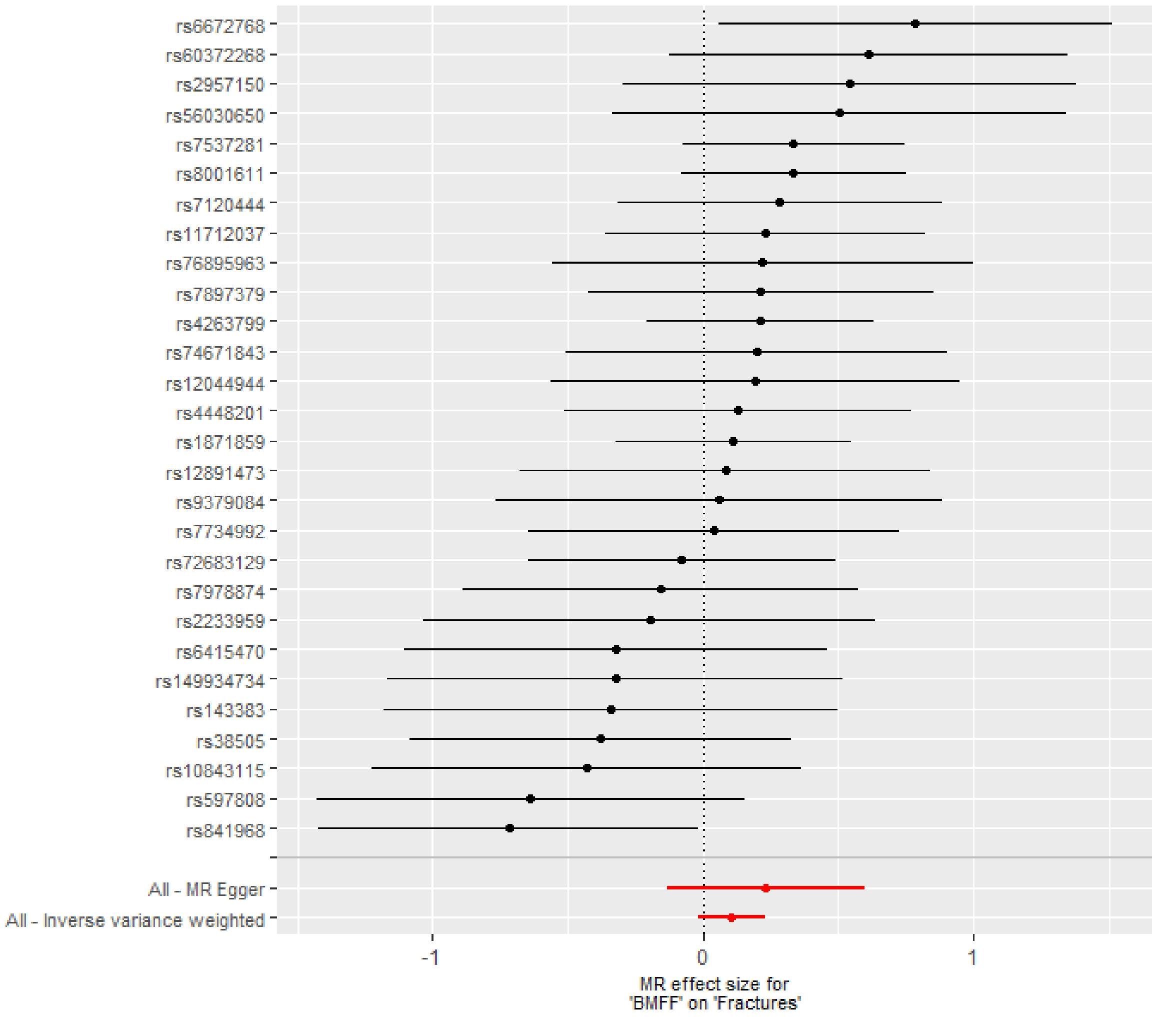


**Supplementary Figure 29 –** MR-forest plot for total hip BMFF-Fractures.

The x-axis represents the effect size with the corresponding 95% CIs. Each black point represents the log OR for fractures per SD increase in BMFF, produced using each of the 'BMFF SNPs' (clump r2<0.001) as separate instruments, and red points showing the combined causal estimate using all SNPs together in a single instrument, using IVW random effects and MR-Egger. Horizontal lines denote 95% CI.

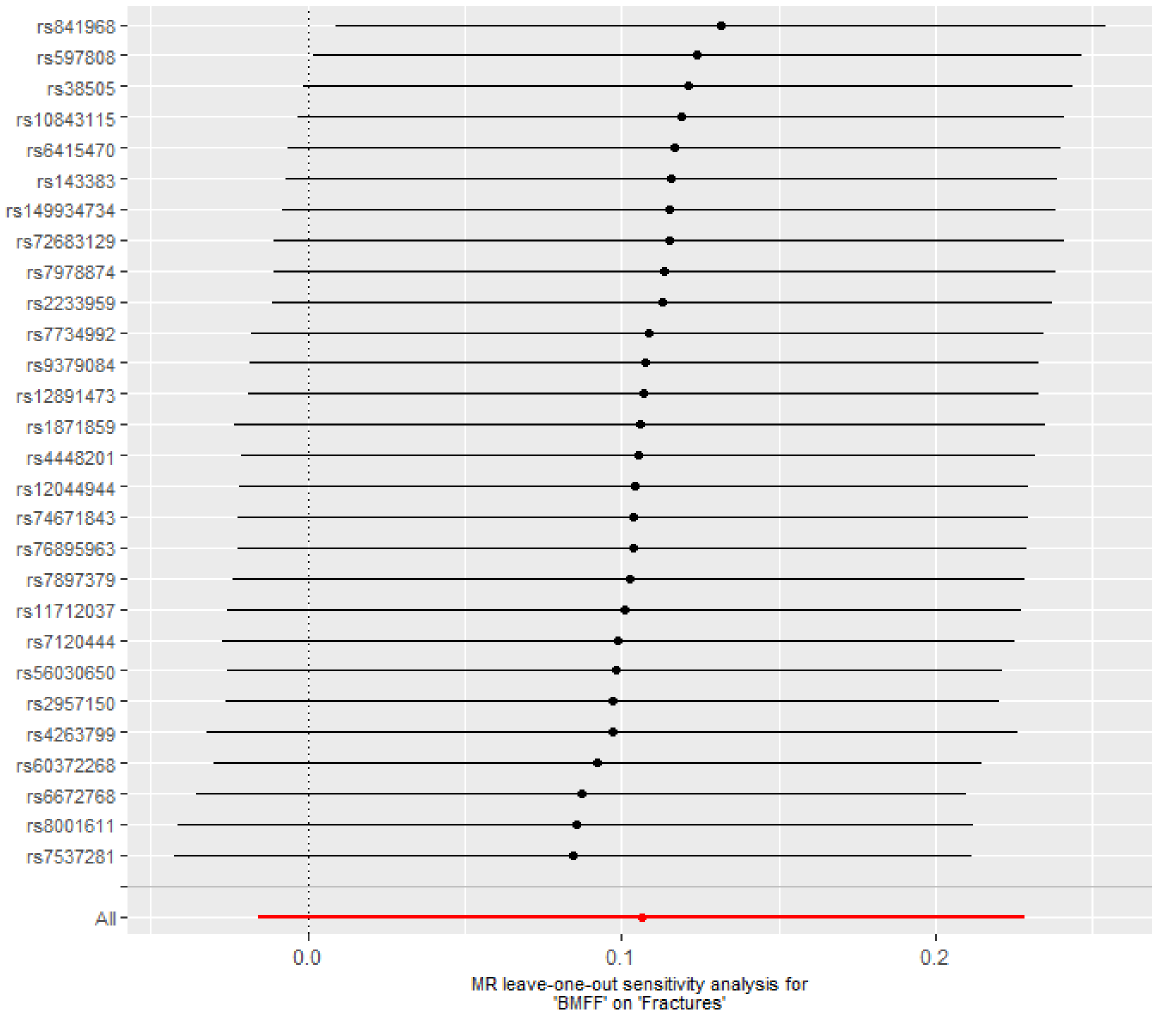


**Supplementary Figure 30 –** Forest plot of leave-one-out sensitivity results for total hip BMFF-Fractures.

Each black point represents the IVW MR method applied to estimate the causal effect of BMFF on fractures excluding that particular variant from the analysis. The red point depicts the IVW estimate using all SNPs. There are no instances where the exclusion of one particular SNP leads to dramatic changes in the overall result.

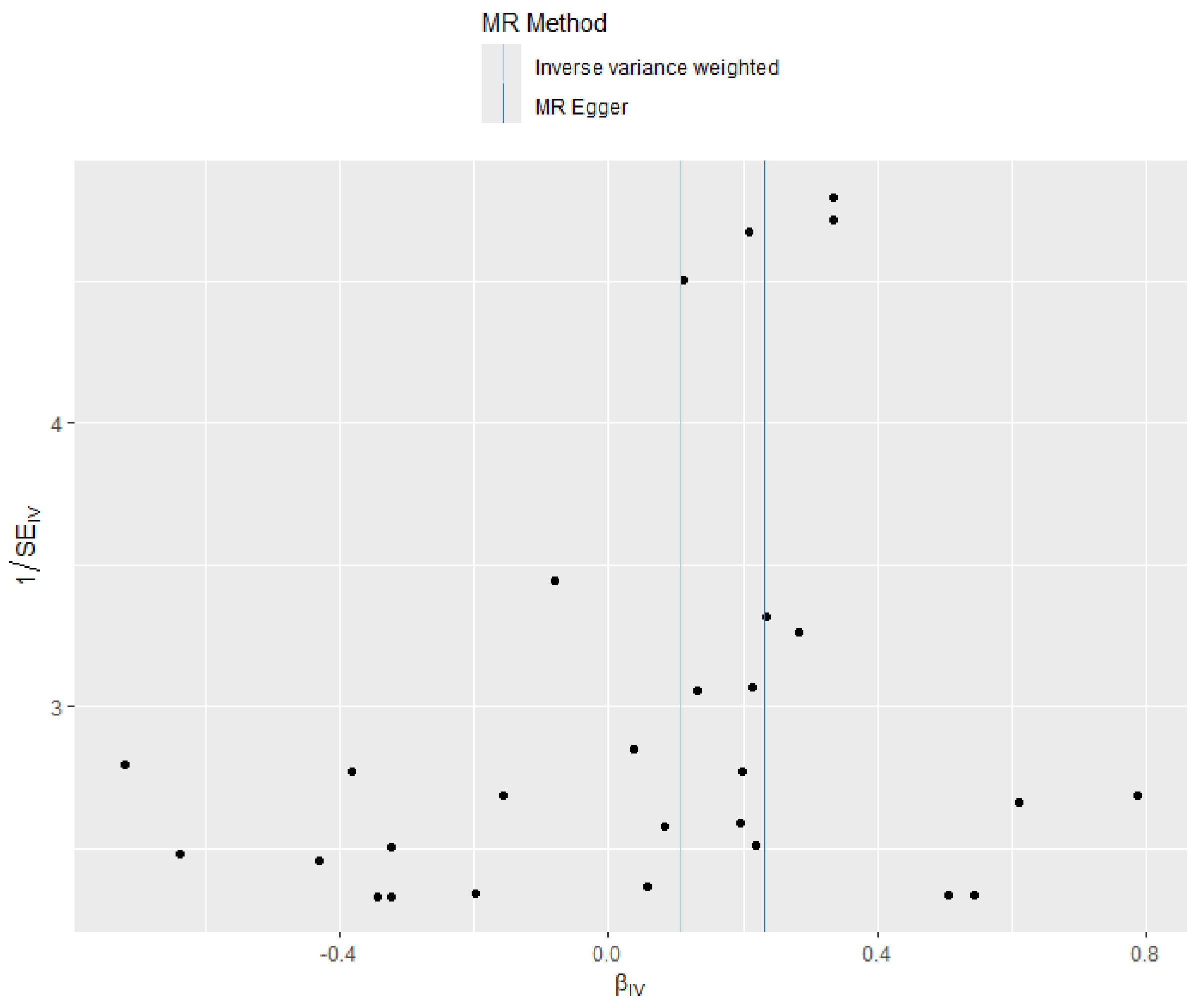


**Supplementary Figure 31 –** MR-funnel plot of total hip BMFF-Fractures.

Vertical lines show the causal estimates using all SNPs combined into a single instrument for IVW random effects and MR-Egger methods.

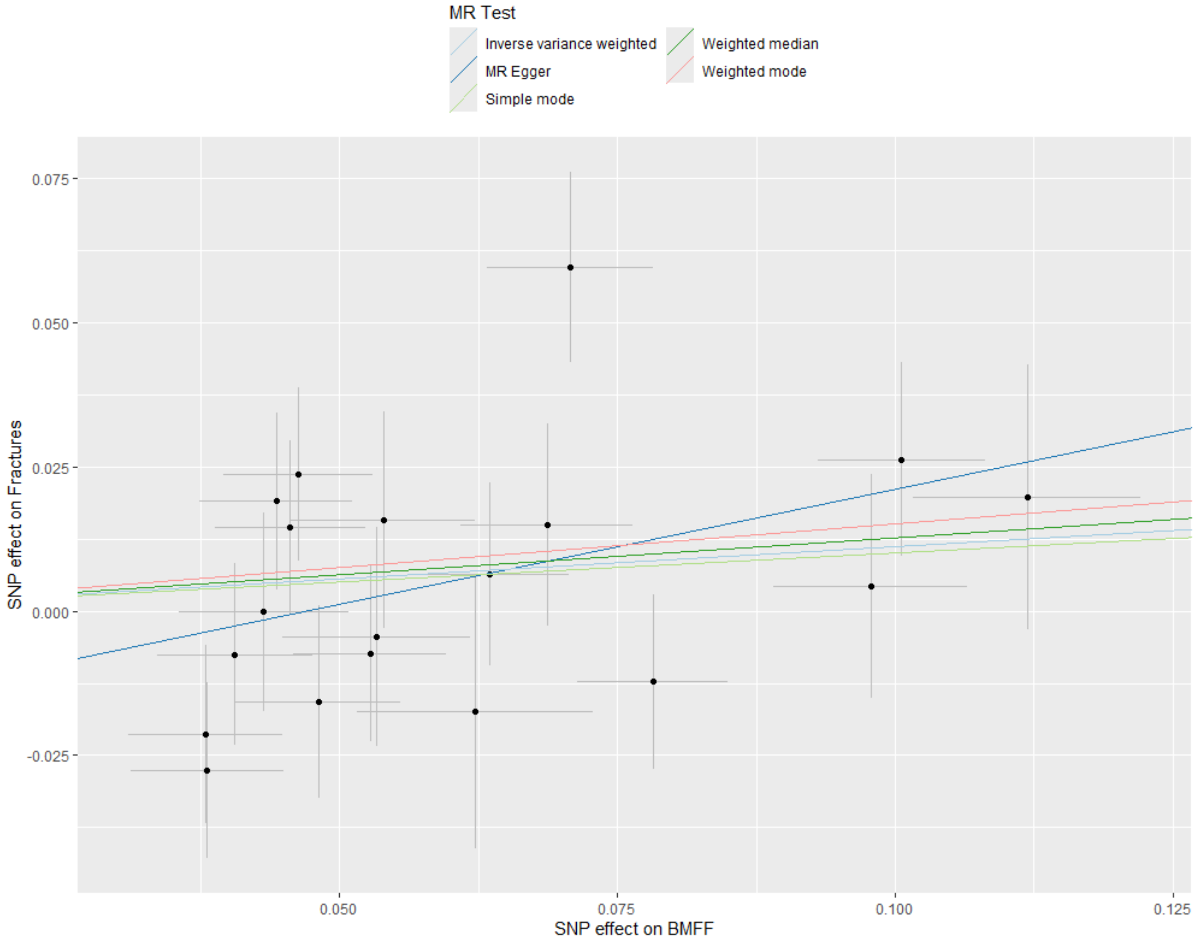


**Supplementary Figure 32 –** MR-scatter plot for diaphysis BMFF-Fractures.

Each dot represents a SNP (clump r2<0.001), with the x-axis showing the SNP effect on BMFF and the y-axis showing the SNP effect on fractures. The fitted lines represent different MR estimation methods, including: inverse variance weighted (IVW; (light blue), MR Egger (dark blue), weighted mode (red), simple mode (light green), weighted median (dark green).

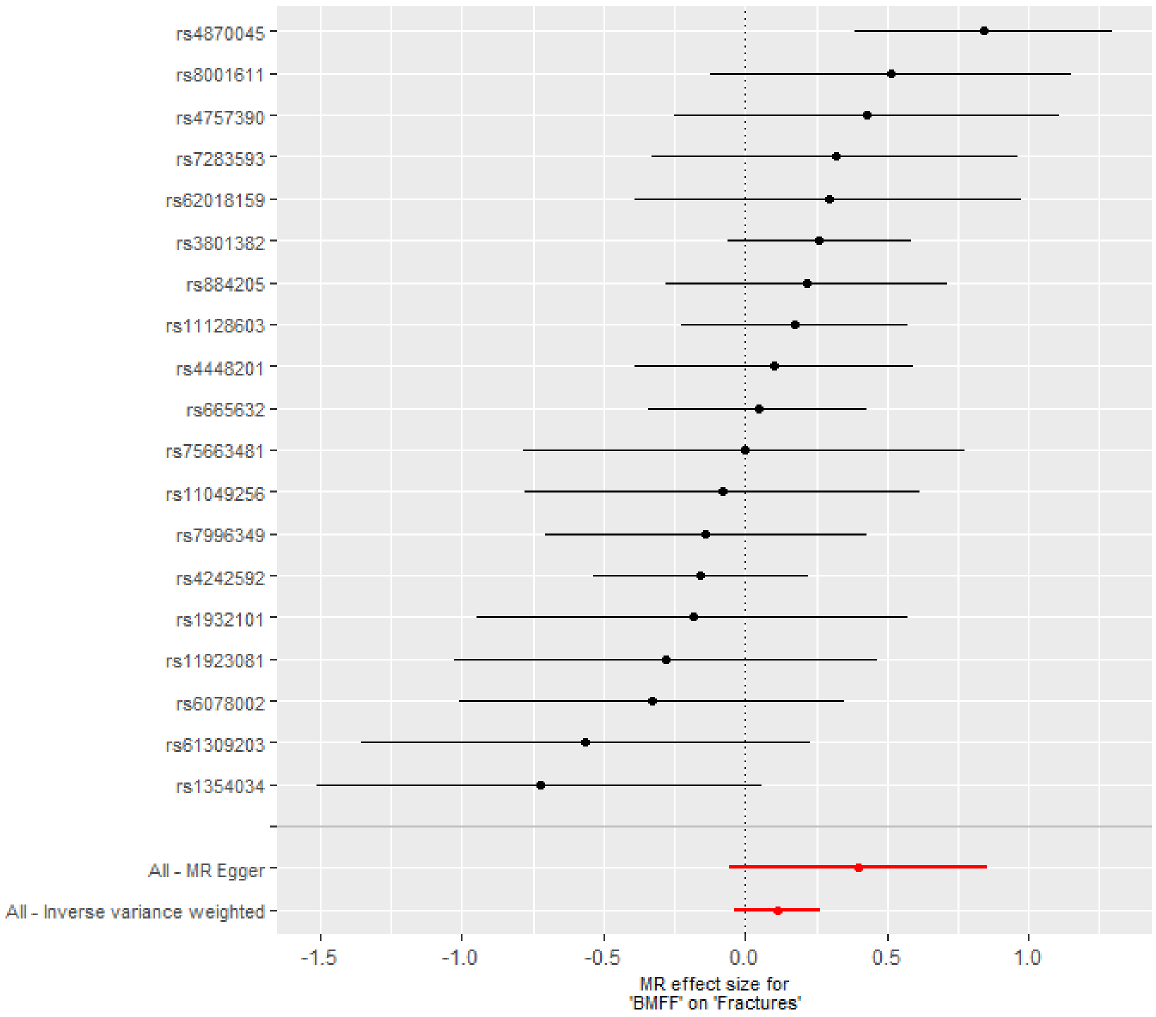


**Supplementary Figure 33 –** MR-forest plot for diaphysis BMFF-Fractures.

The x-axis represents the effect size with the corresponding 95% CIs. Each black point represents the log OR for fractures per SD increase in BMFF, produced using each of the 'BMFF SNPs' (clump r2<0.001) as separate instruments, and red points showing the combined causal estimate using all SNPs together in a single instrument, using IVW random effects and MR-Egger. Horizontal lines denote 95% CI.

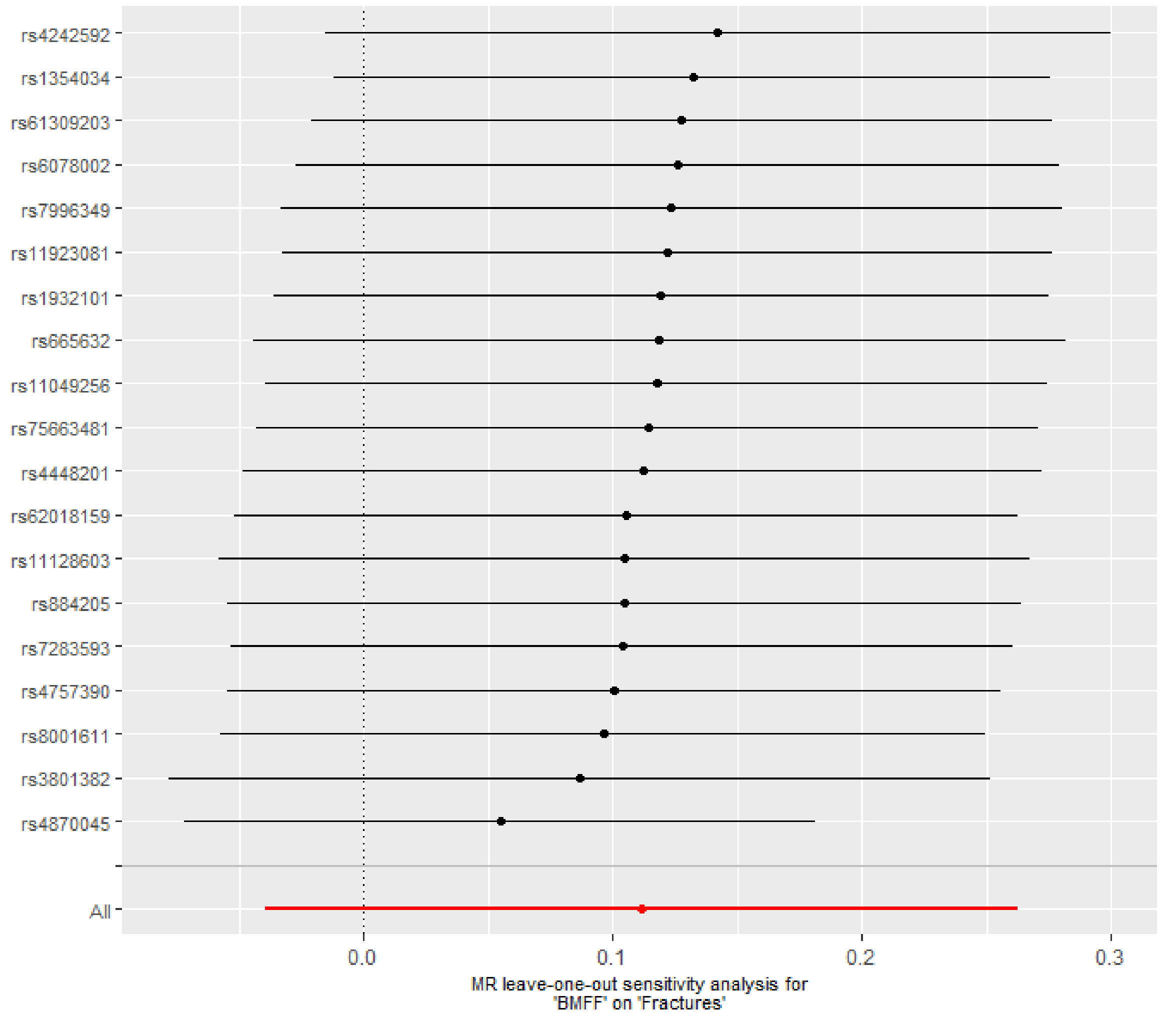


**Supplementary Figure 34 –** Forest plot of leave-one-out sensitivity results for diaphysis BMFF-Fractures.

Each black point represents the IVW MR method applied to estimate the causal effect of BMFF on fractures excluding that particular variant from the analysis. The red point depicts the IVW estimate using all SNPs. There are no instances where the exclusion of one particular SNP leads to dramatic changes in the overall result.

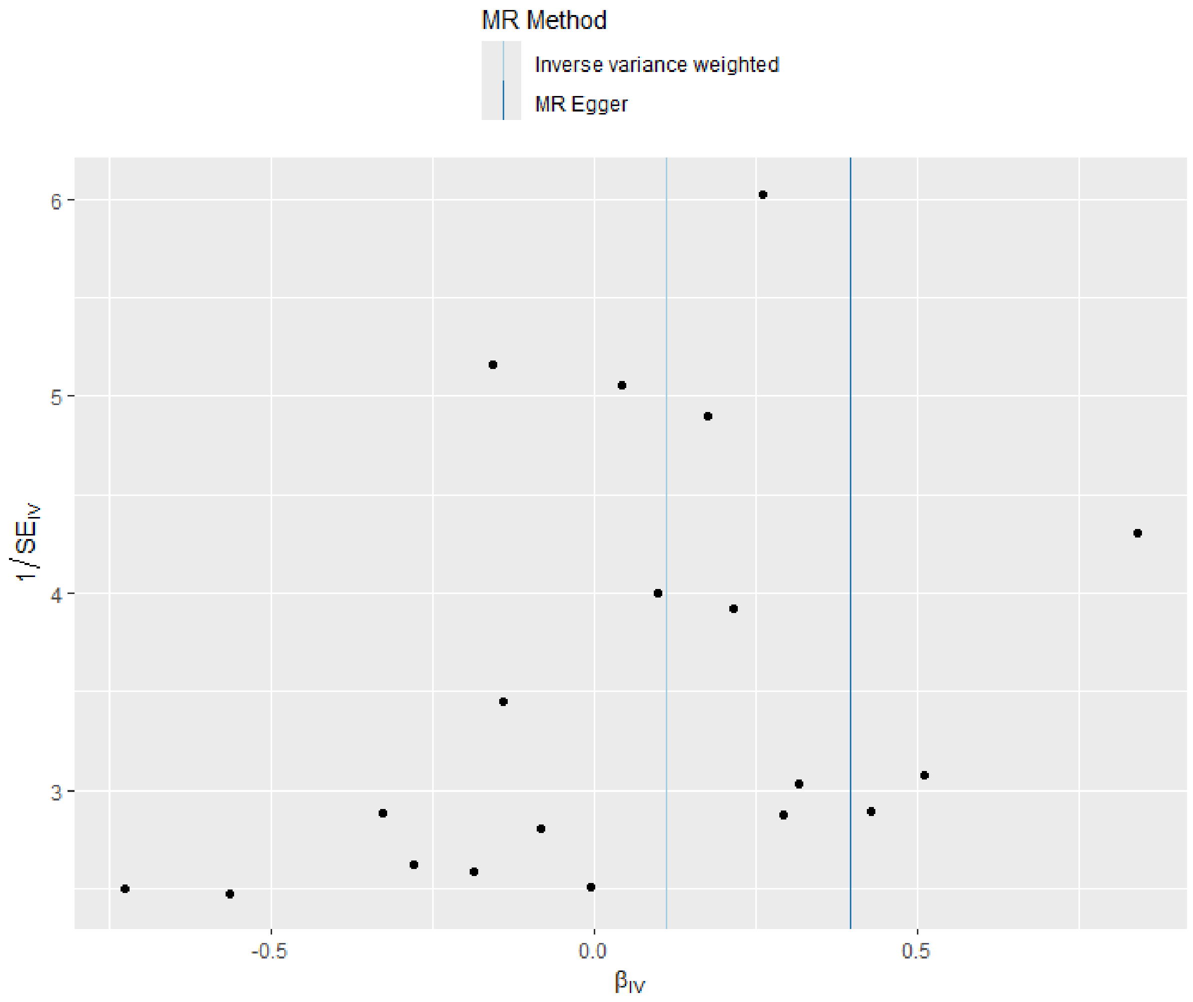


**Supplementary Figure 35 –** MR-funnel plot of diaphysis BMFF-Fractures.

Vertical lines show the causal estimates using all SNPs combined into a single instrument for IVW random effects and MR-Egger methods.

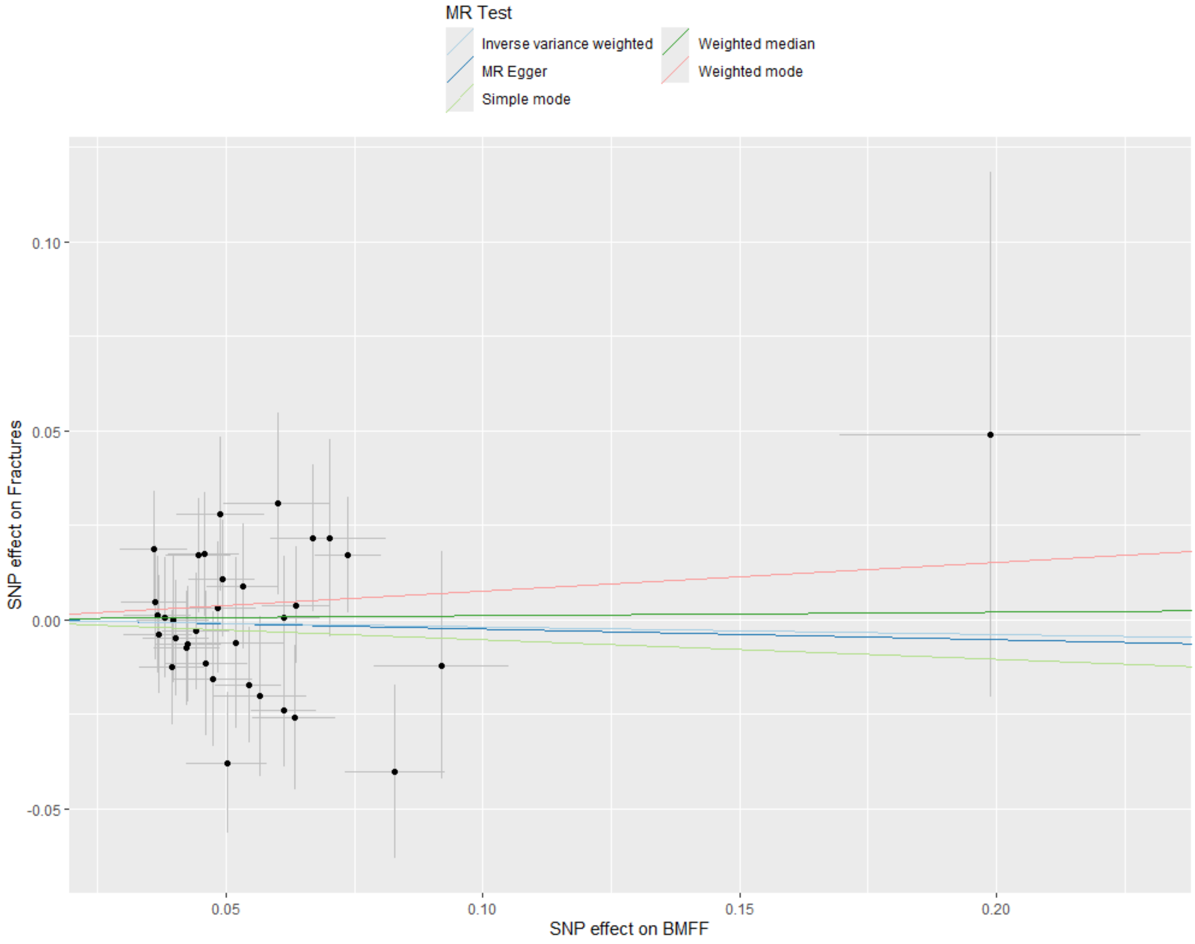


**Supplementary Figure 36 –** MR-scatter plot for spine BMFF-Fractures.

Each dot represents a SNP (clump r2<0.001), with the x-axis showing the SNP effect on BMFF and the y-axis showing the SNP effect on fractures. The fitted lines represent different MR estimation methods, including: inverse variance weighted (IVW; (light blue), MR Egger (dark blue), weighted mode (red), simple mode (light green), weighted median (dark green).

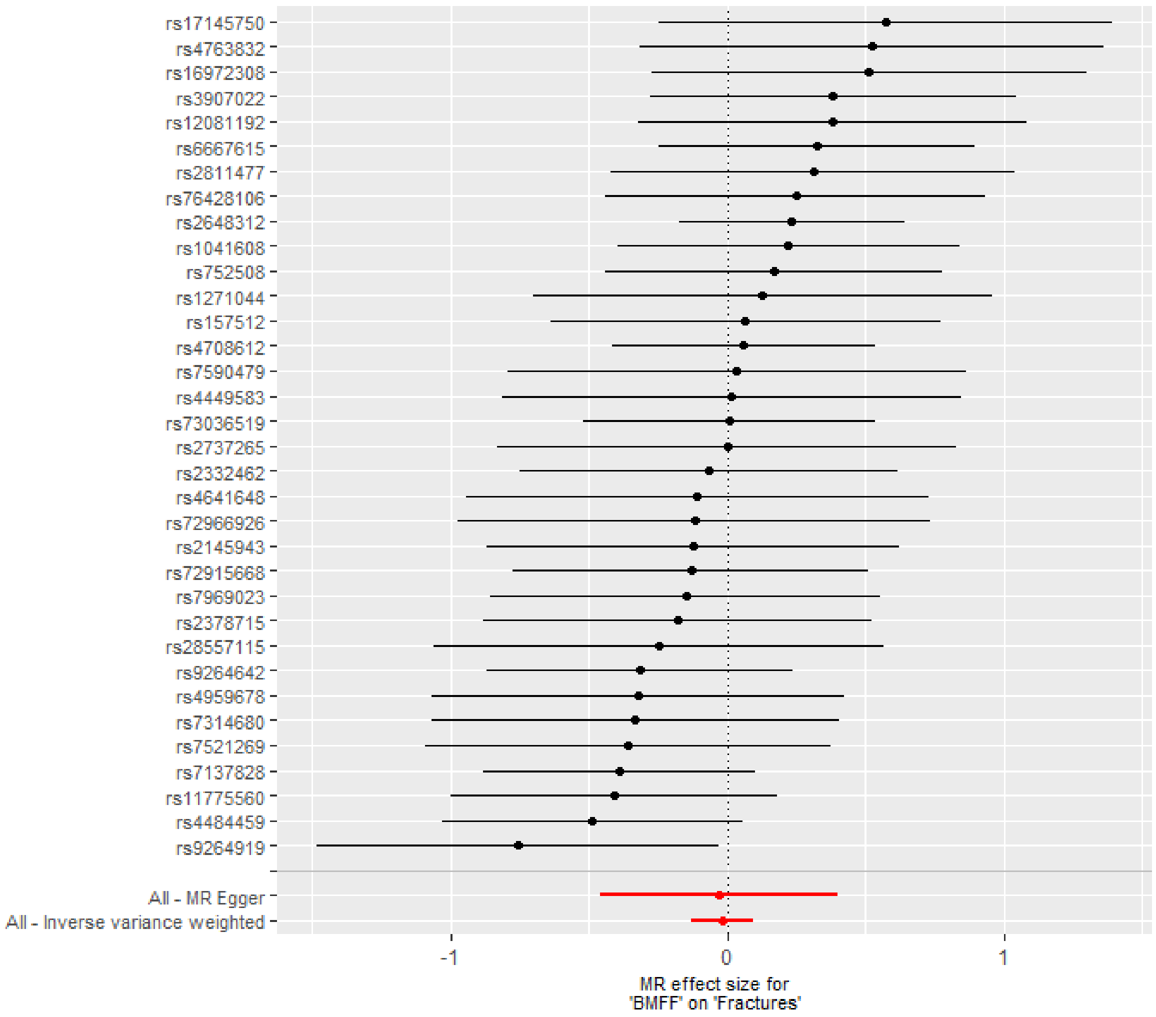


**Supplementary Figure 37 –** MR-forest plot for spine BMFF-Fractures.

The x-axis represents the effect size with the corresponding 95% CIs. Each black point represents the log OR for fractures per SD increase in BMFF, produced using each of the 'BMFF SNPs' (clump r2<0.001) as separate instruments, and red points showing the combined causal estimate using all SNPs together in a single instrument, using IVW random effects and MR-Egger. Horizontal lines denote 95% CI.

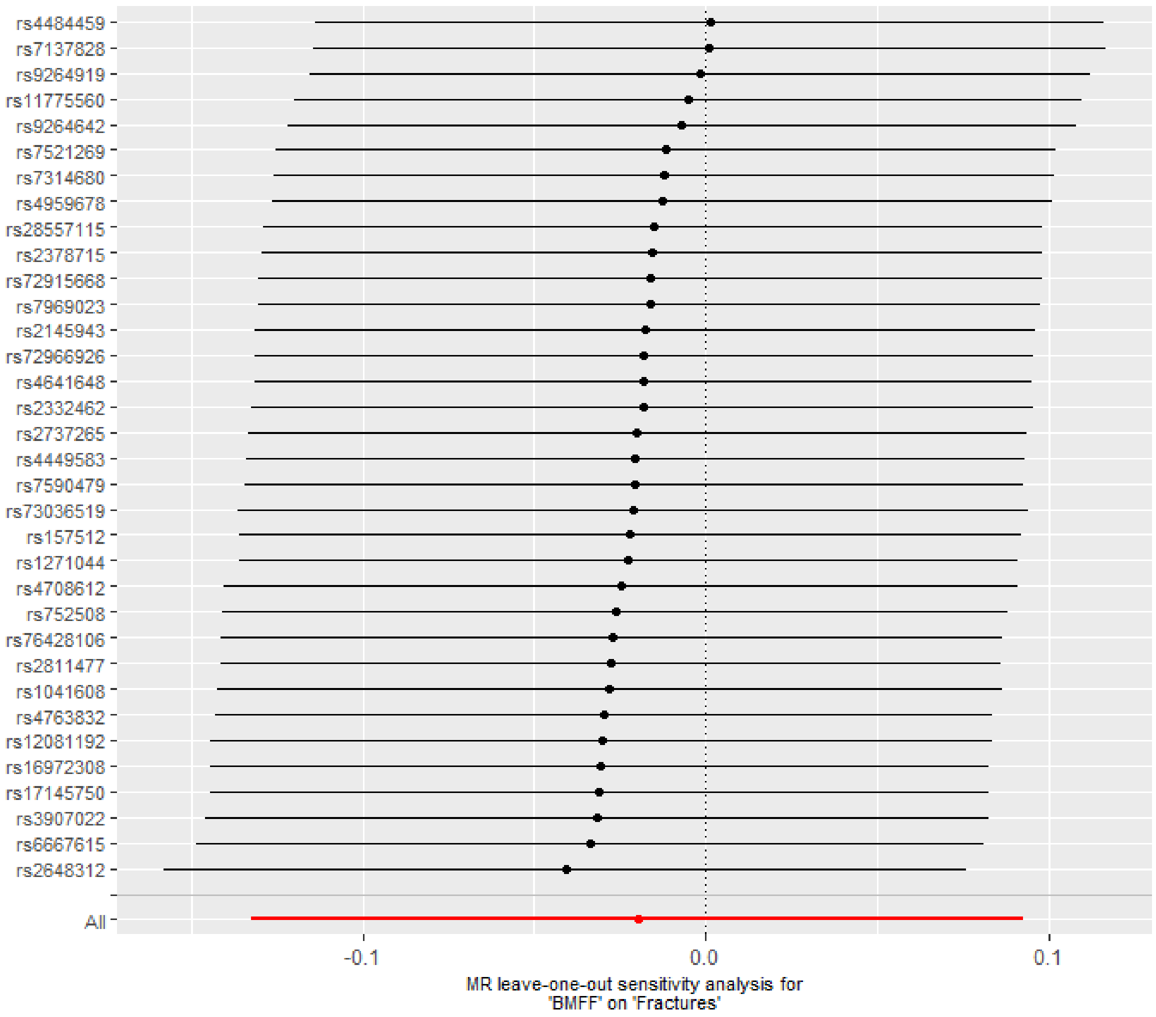


**Supplementary Figure 38 –** Forest plot of leave-one-out sensitivity results for spine BMFF-Fractures.

Each black point represents the IVW MR method applied to estimate the causal effect of BMFF on fractures excluding that particular variant from the analysis. The red point depicts the IVW estimate using all SNPs. There are no instances where the exclusion of one particular SNP leads to dramatic changes in the overall result.

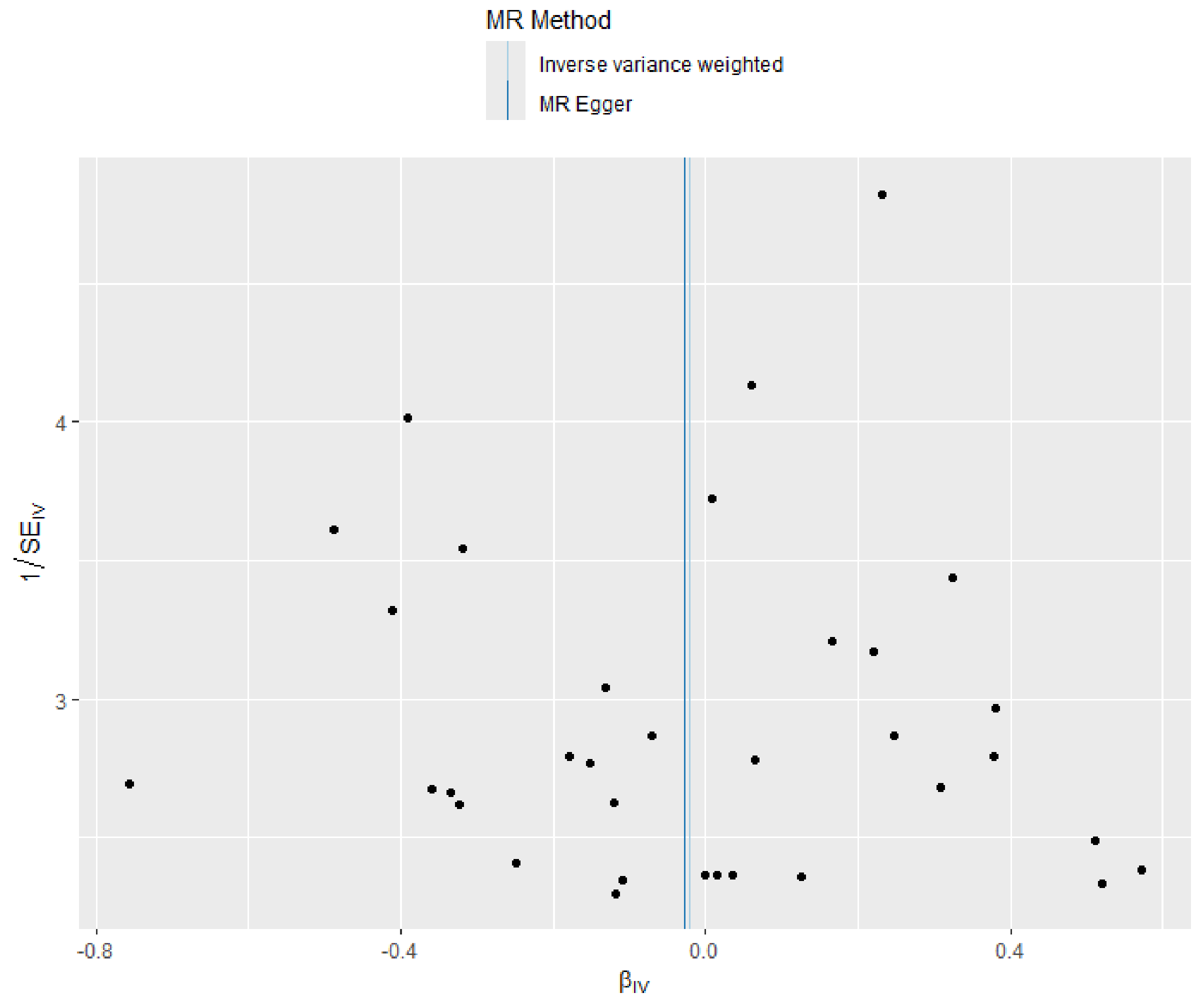


**Supplementary Figure 39 –** MR-funnel plot of spine BMFF-Fractures.

Vertical lines show the causal estimates using all SNPs combined into a single instrument for IVW random effects and MR-Egger methods.

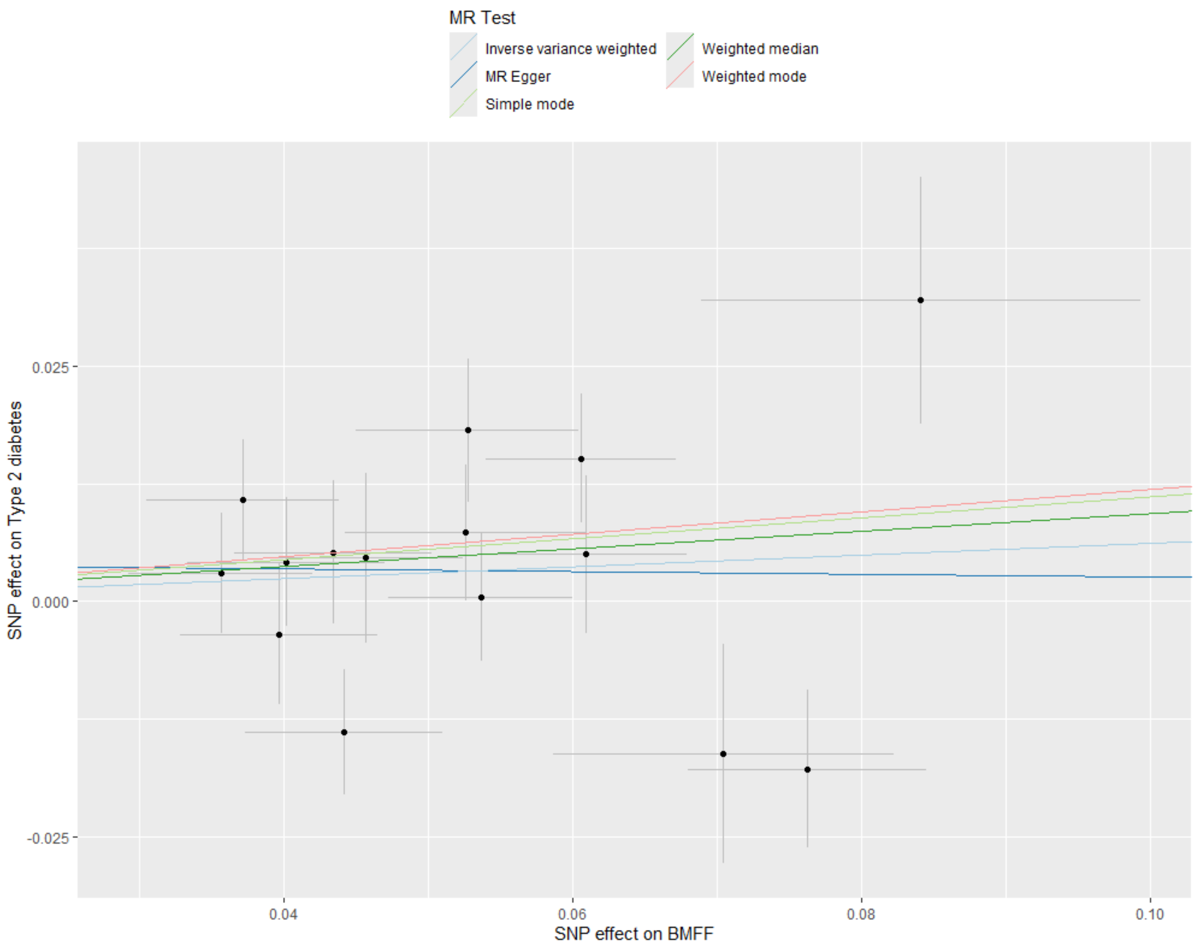


**Supplementary Figure 40 –** MR-scatter plot for femoral head BMFF-Type 2 diabetes.

Each dot represents a SNP (clump r2<0.001), with the x-axis showing the SNP effect on BMFF and the y-axis showing the SNP effect on type 2 diabetes. The fitted lines represent different MR estimation methods, including: inverse variance weighted (IVW; (light blue), MR Egger (dark blue), weighted mode (red), simple mode (light green), weighted median (dark green).

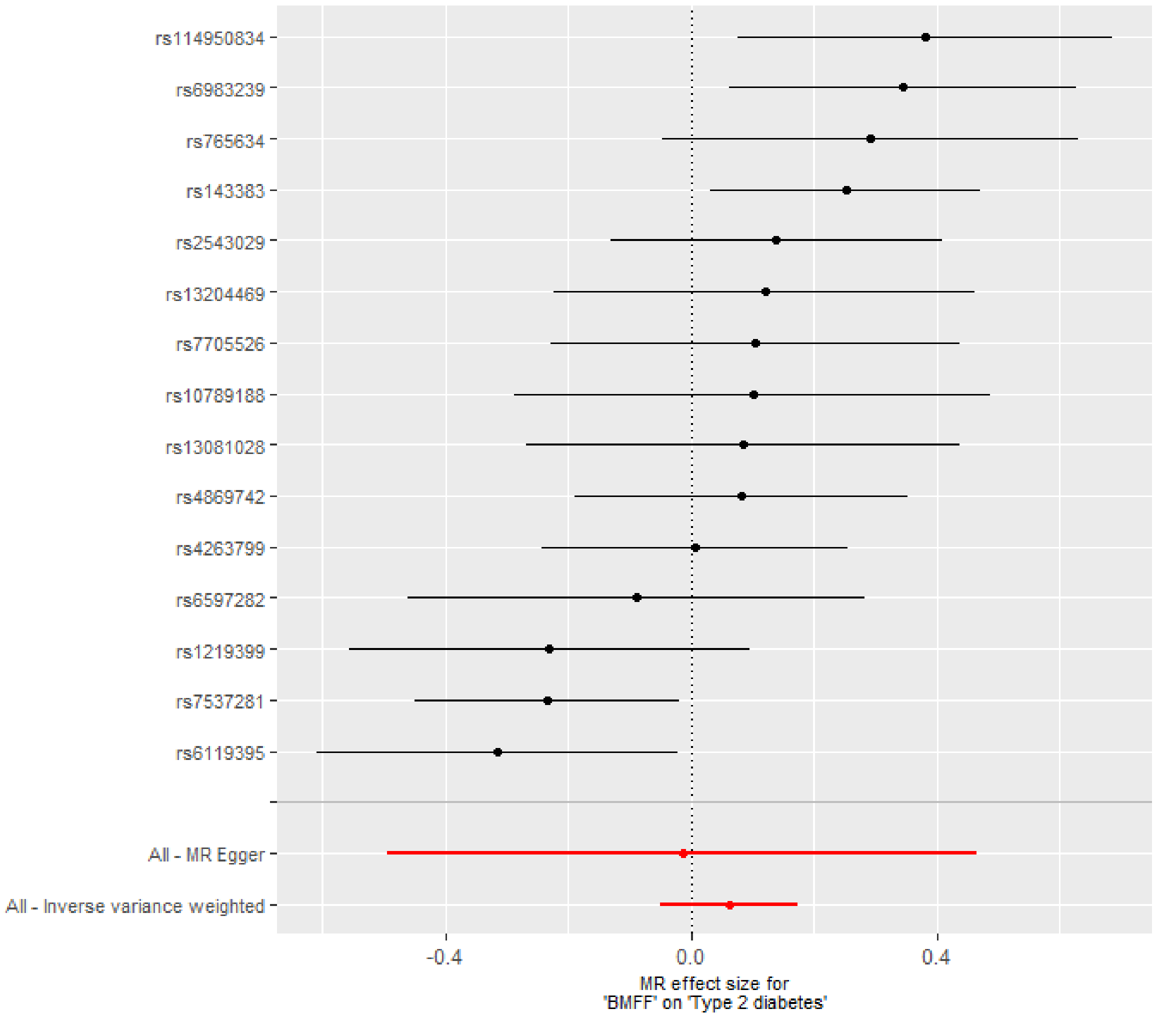


**Supplementary Figure 41 –** MR-forest plot for femoral head BMFF-Type 2 diabetes.

The x-axis represents the effect size with the corresponding 95% CIs. Each black point represents the log OR for type 2 diabetes per SD increase in BMFF, produced using each of the 'BMFF SNPs' (clump r2<0.001) as separate instruments, and red points showing the combined causal estimate using all SNPs together in a single instrument, using IVW random effects and MR-Egger. Horizontal lines denote 95% CI.

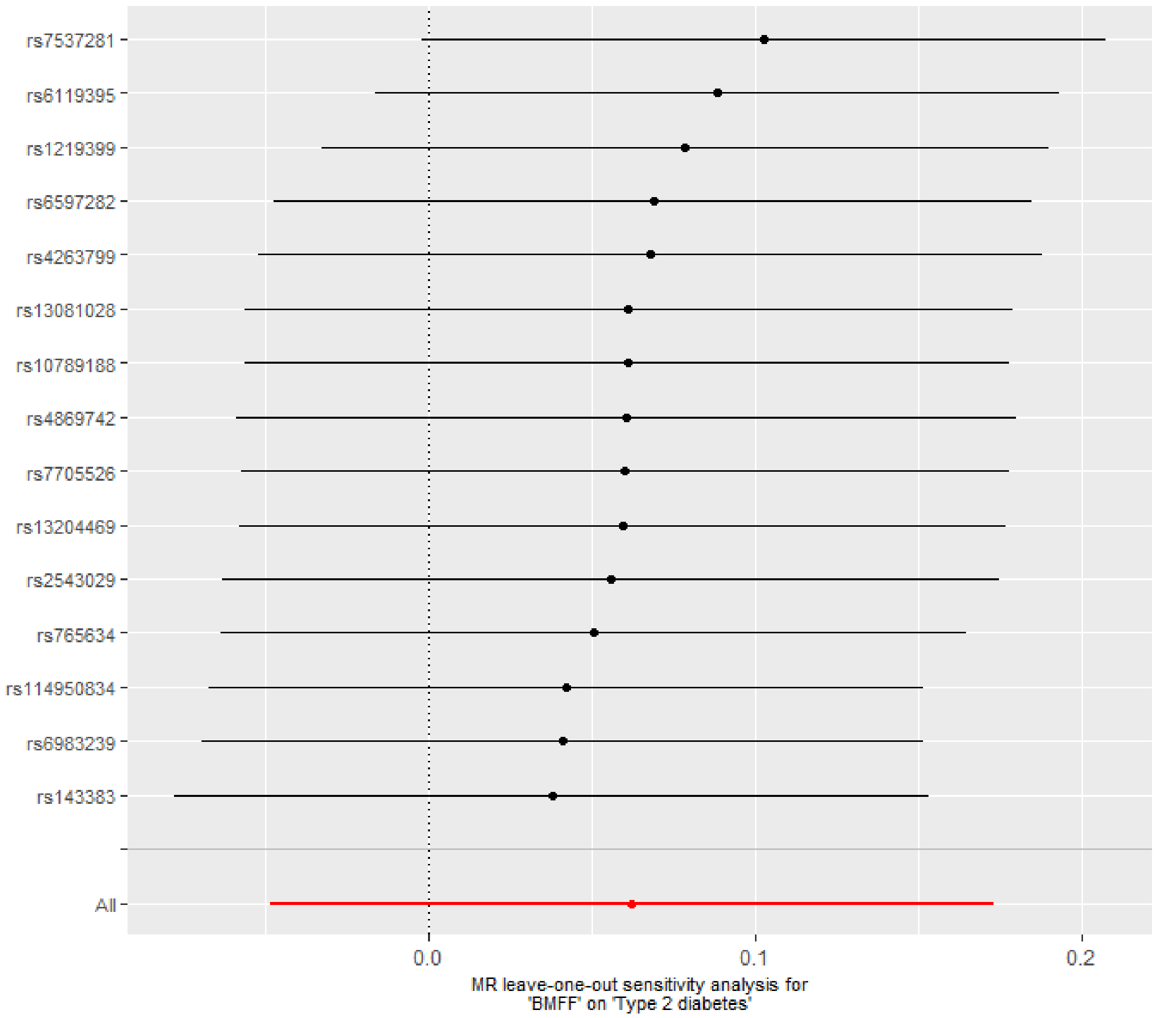


**Supplementary Figure 42 –** Forest plot of leave-one-out sensitivity results for femoral head BMFF-Type 2 diabetes.

Each black point represents the IVW MR method applied to estimate the causal effect of BMFF on type 2 diabetes excluding that particular variant from the analysis. The red point depicts the IVW estimate using all SNPs. There are no instances where the exclusion of one particular SNP leads to dramatic changes in the overall result.

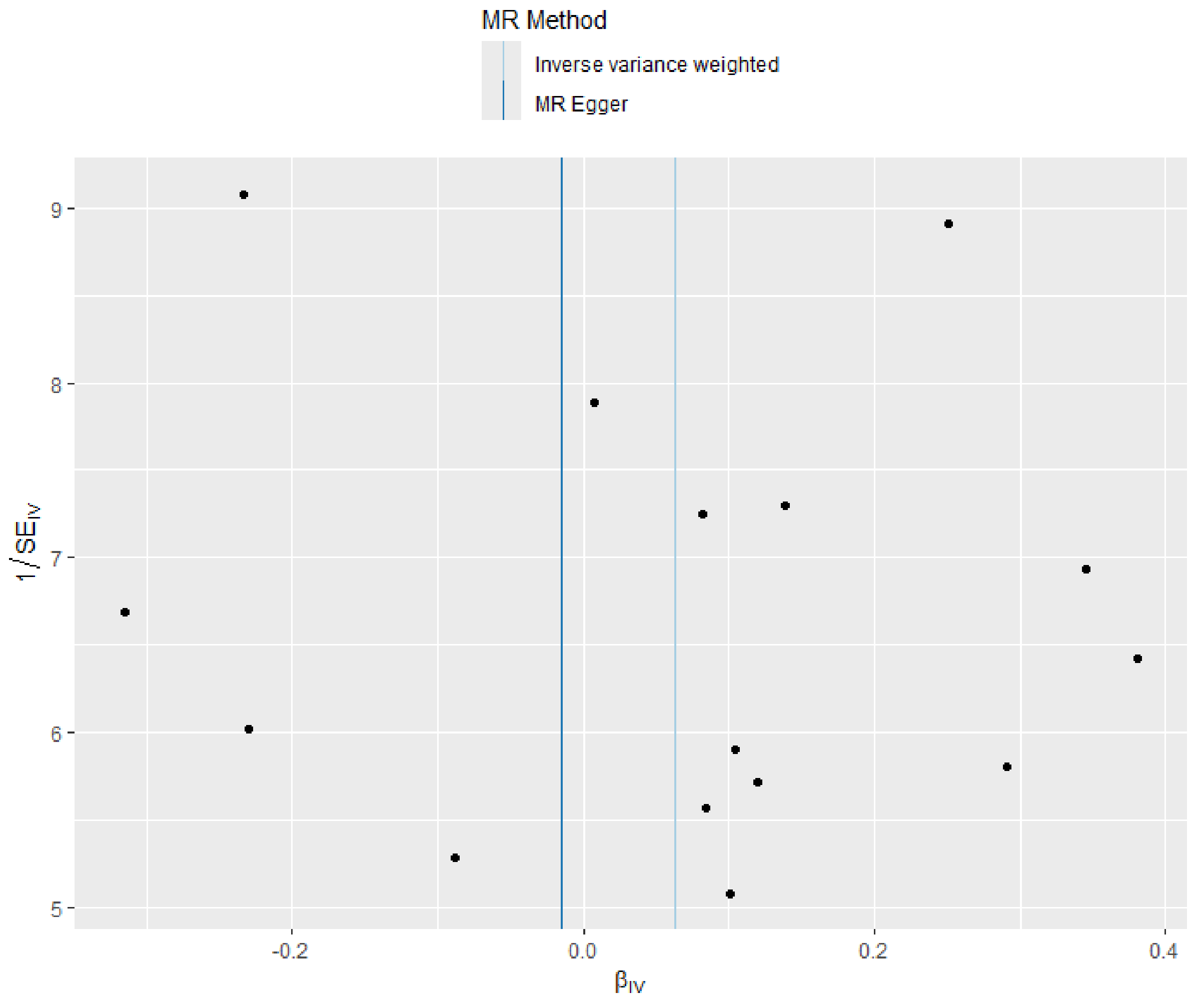


**Supplementary Figure 43 –** MR-funnel plot of femoral head BMFF-Type 2 diabetes.

Vertical lines show the causal estimates using all SNPs combined into a single instrument for IVW random effects and MR-Egger methods.

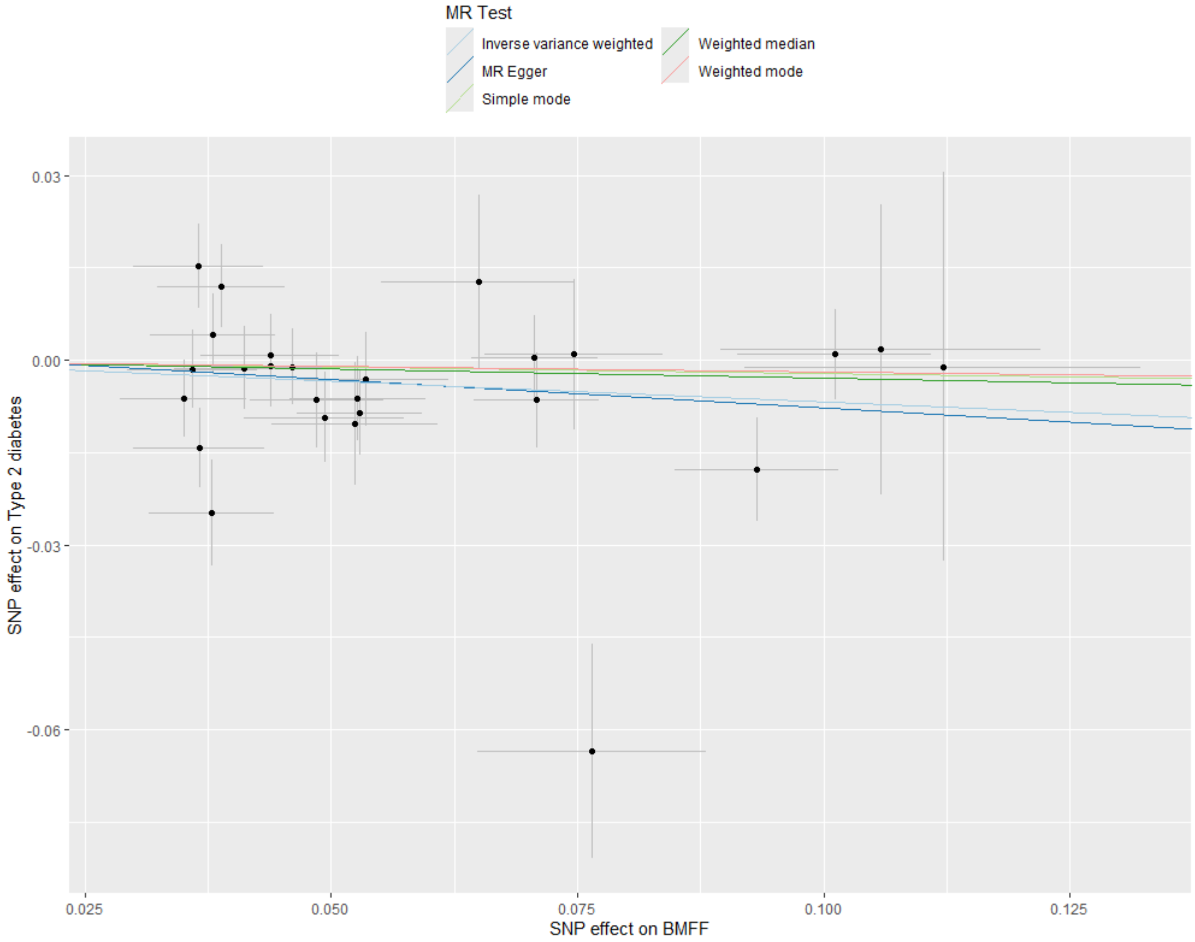


**Supplementary Figure 44 –** MR-scatter plot for total hip BMFF-Type 2 diabetes.

Each dot represents a SNP (clump r2<0.001), with the x-axis showing the SNP effect on BMFF and the y-axis showing the SNP effect on type 2 diabetes. The fitted lines represent different MR estimation methods, including: inverse variance weighted (IVW; (light blue), MR Egger (dark blue), weighted mode (red), simple mode (light green), weighted median (dark green).

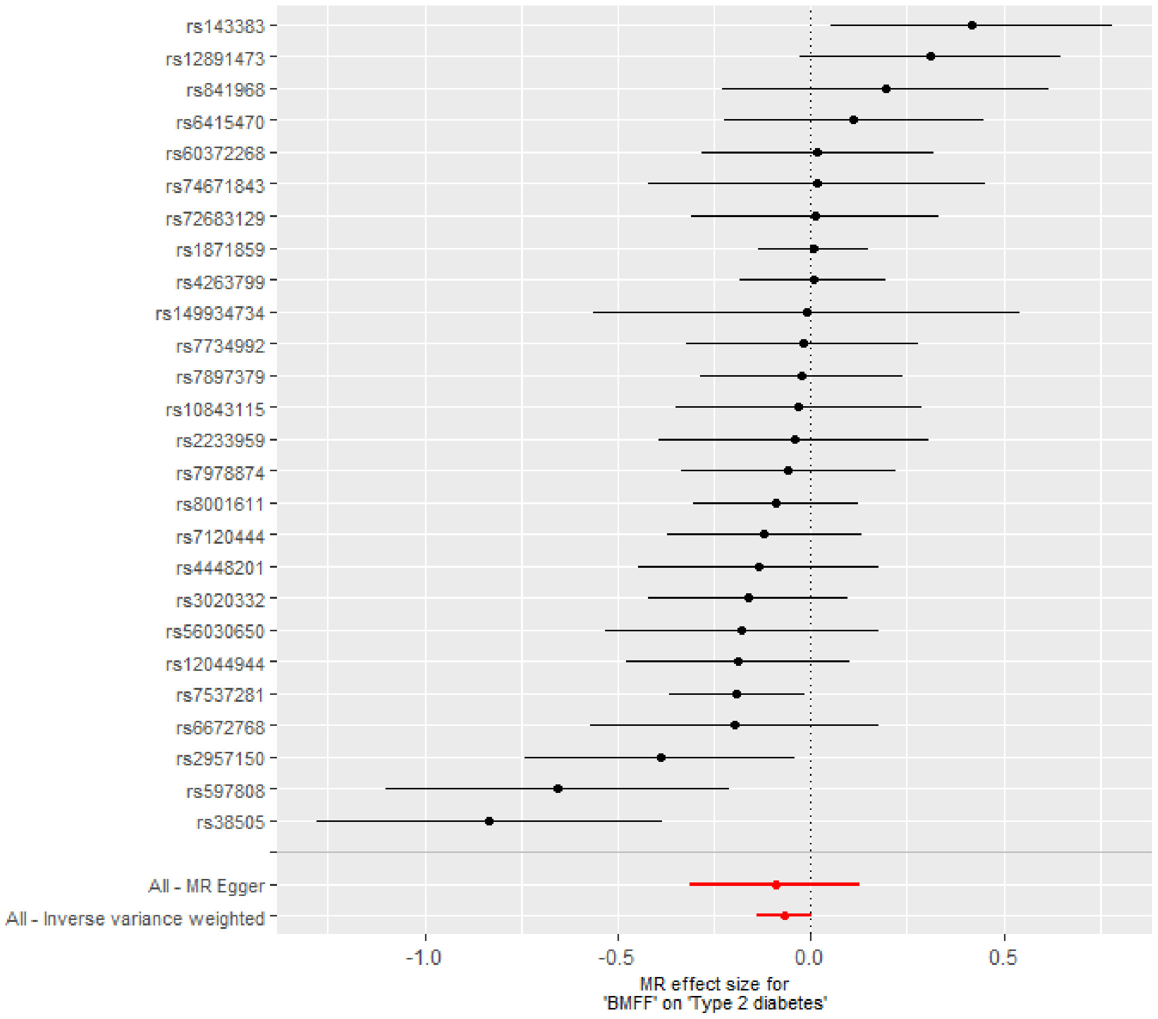


**Supplementary Figure 45 –** MR-forest plot for total hip BMFF-Type 2 diabetes.

The x-axis represents the effect size with the corresponding 95% CIs. Each black point represents the log OR for type 2 diabetes per SD increase in BMFF, produced using each of the ‘BMFF SNPs’ (clump r2<0.001) as separate instruments, and red points showing the combined causal estimate using all SNPs together in a single instrument, using IVW random effects and MR-Egger. Horizontal lines denote 95% CI.

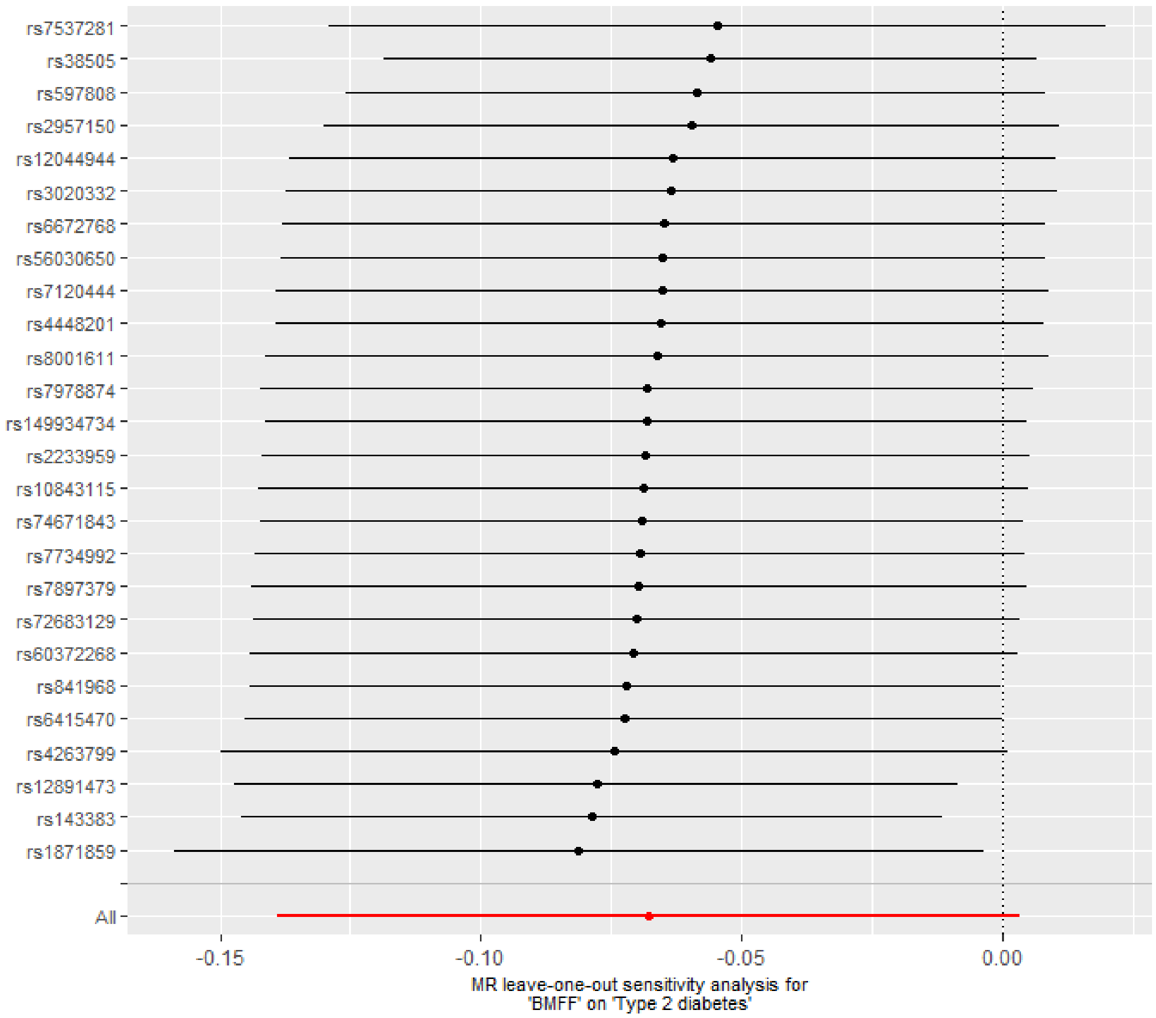


**Supplementary Figure 46 –** Forest plot of leave-one-out sensitivity results for total hip BMFF-Type 2 diabetes.

Each black point represents the IVW MR method applied to estimate the causal effect of BMFF on type 2 diabetes excluding that particular variant from the analysis. The red point depicts the IVW estimate using all SNPs. There are no instances where the exclusion of one particular SNP leads to dramatic changes in the overall result.

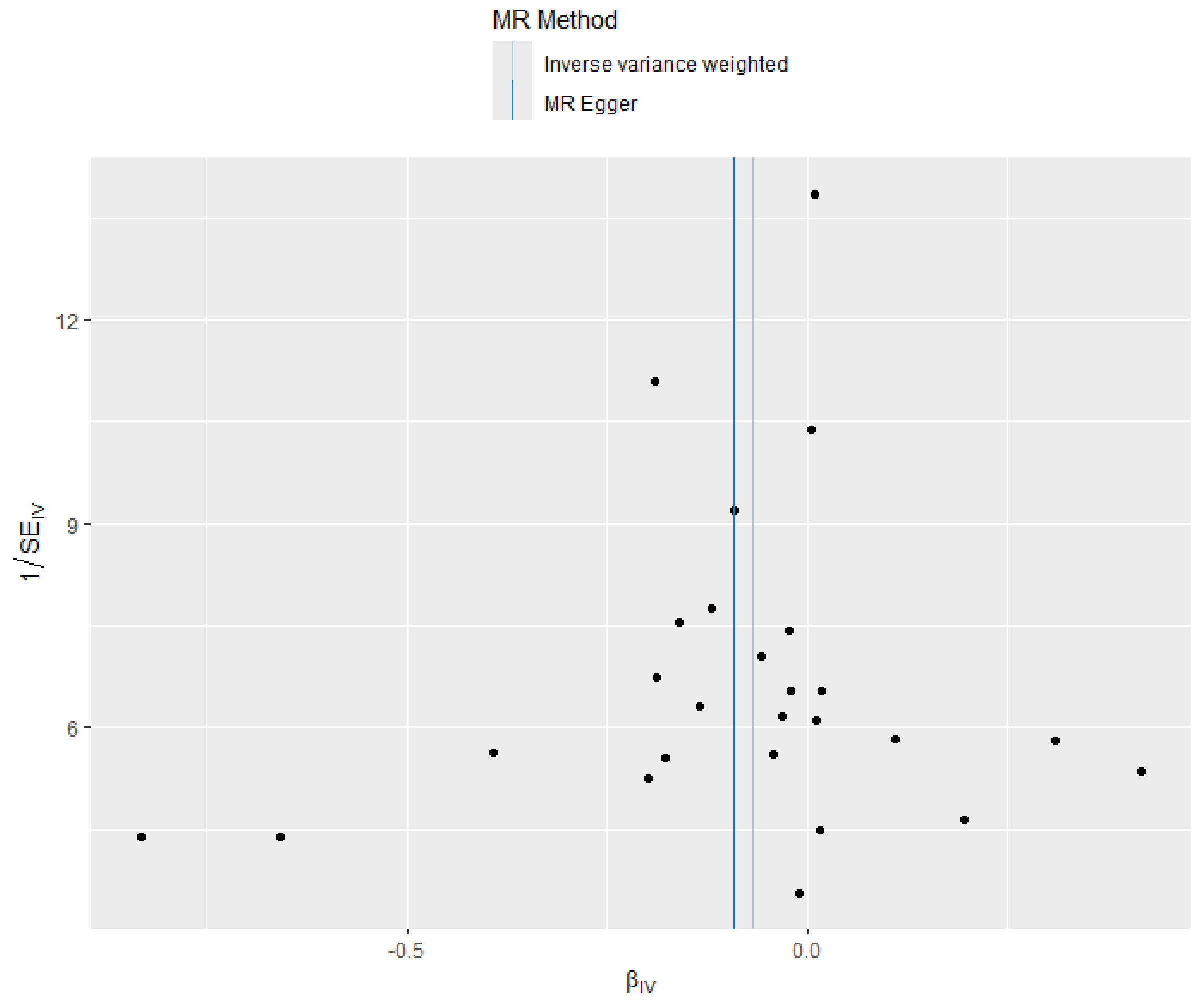


**Supplementary Figure 47 –** MR-funnel plot of total hip BMFF-Type 2 diabetes.

Vertical lines show the causal estimates using all SNPs combined into a single instrument for IVW random effects and MR-Egger methods.

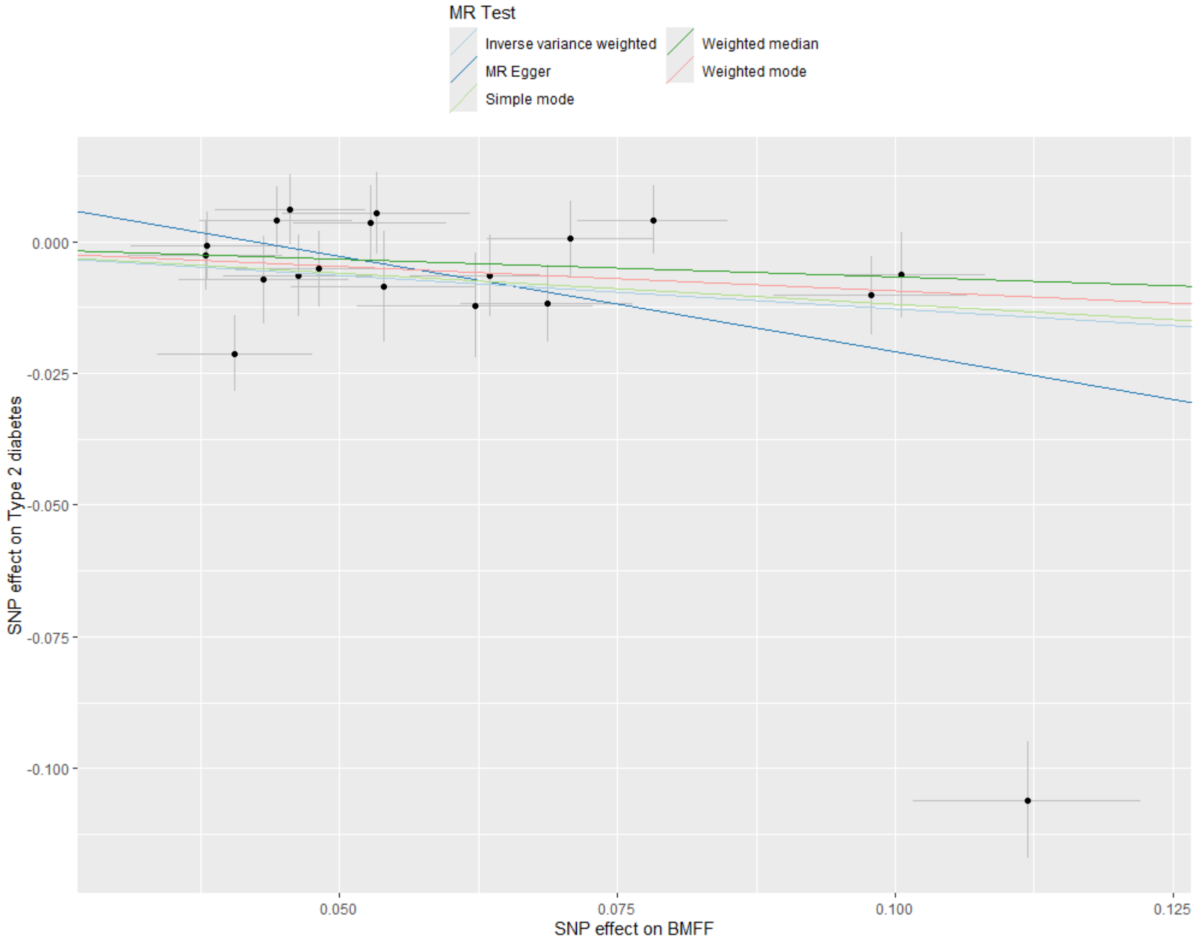


**Supplementary Figure 48 –** MR-scatter plot for diaphysis BMFF-Type 2 diabetes.

Each dot represents a SNP (clump r2<0.001), with the x-axis showing the SNP effect on BMFF and the y-axis showing the SNP effect on type 2 diabetes. The fitted lines represent different MR estimation methods, including: inverse variance weighted (IVW; (light blue), MR Egger (dark blue), weighted mode (red), simple mode (light green), weighted median (dark green).

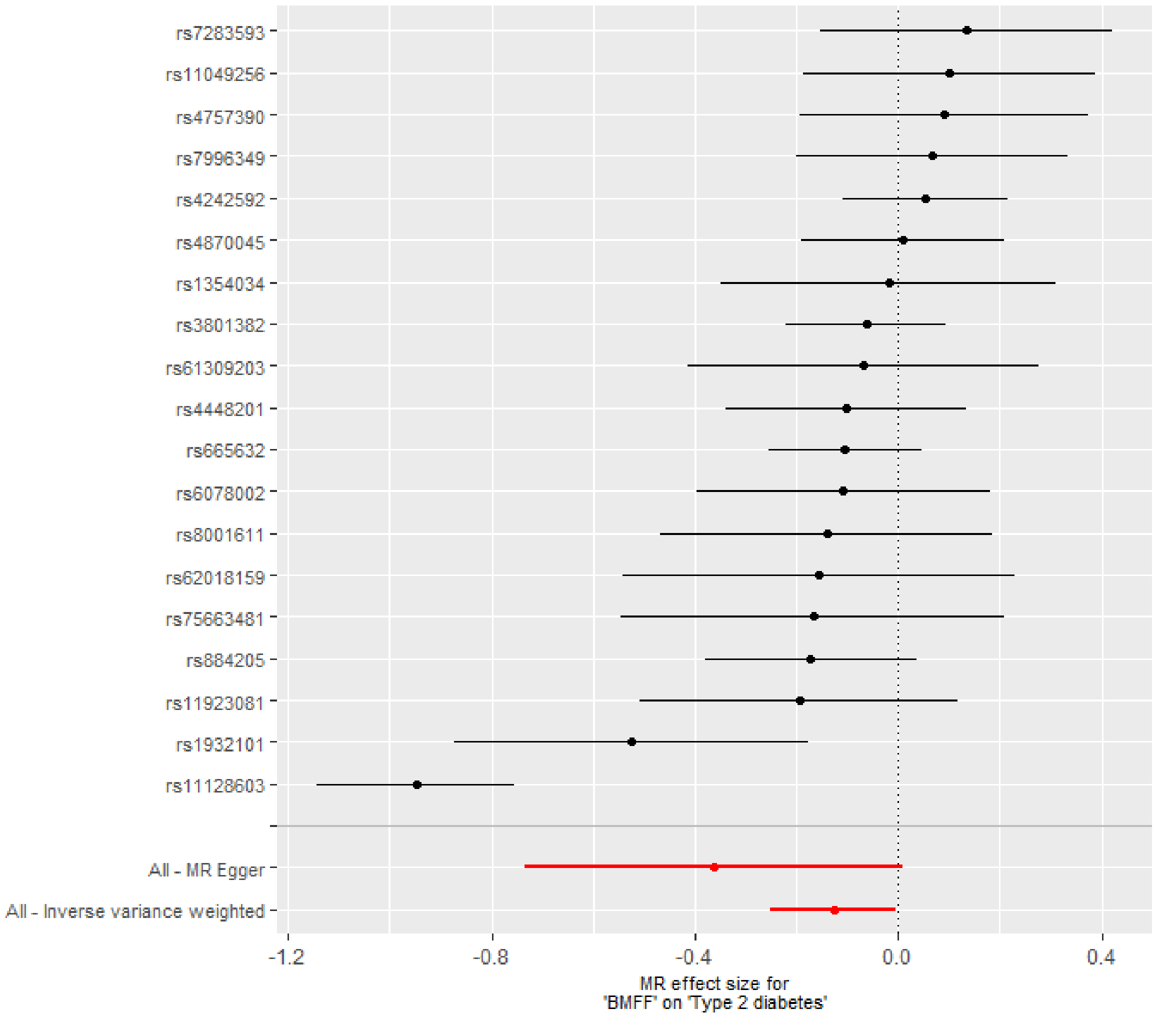


**Supplementary Figure 49 –** MR-forest plot for diaphysis BMFF-Type 2 diabetes.

The x-axis represents the effect size with the corresponding 95% CIs. Each black point represents the log OR for type 2 diabetes per SD increase in BMFF, produced using each of the 'BMFF SNPs' (clump r2<0.001) as separate instruments, and red points showing the combined causal estimate using all SNPs together in a single instrument, using IVW random effects and MR-Egger. Horizontal lines denote 95% CI.

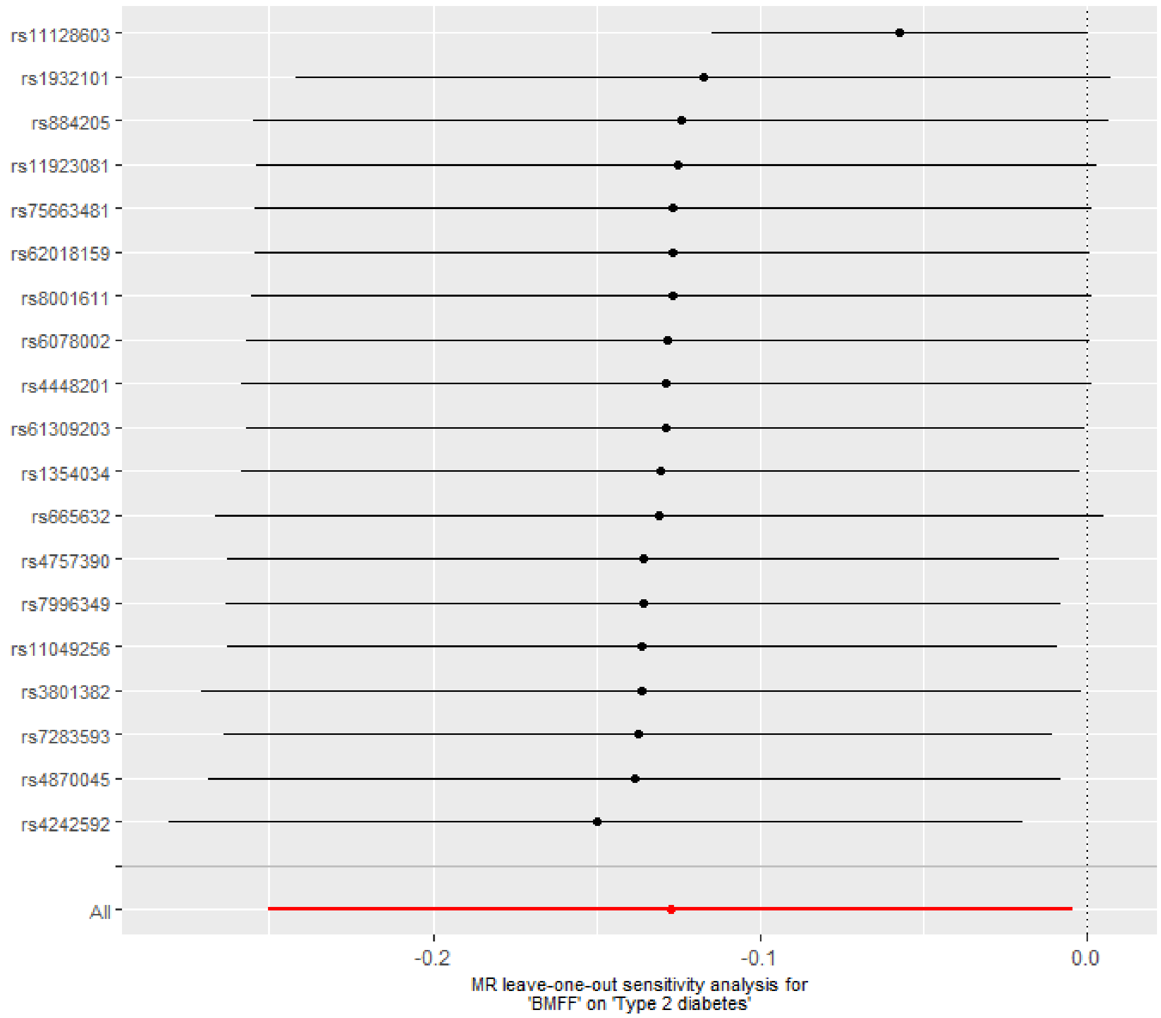


**Supplementary Figure 50 –** Forest plot of leave-one-out sensitivity results for diaphysis BMFF-Type 2 diabetes.

Each black point represents the IVW MR method applied to estimate the causal effect of BMFF on type 2 diabetes excluding that particular variant from the analysis. The red point depicts the IVW estimate using all SNPs. There are no instances where the exclusion of one particular SNP leads to dramatic changes in the overall result.

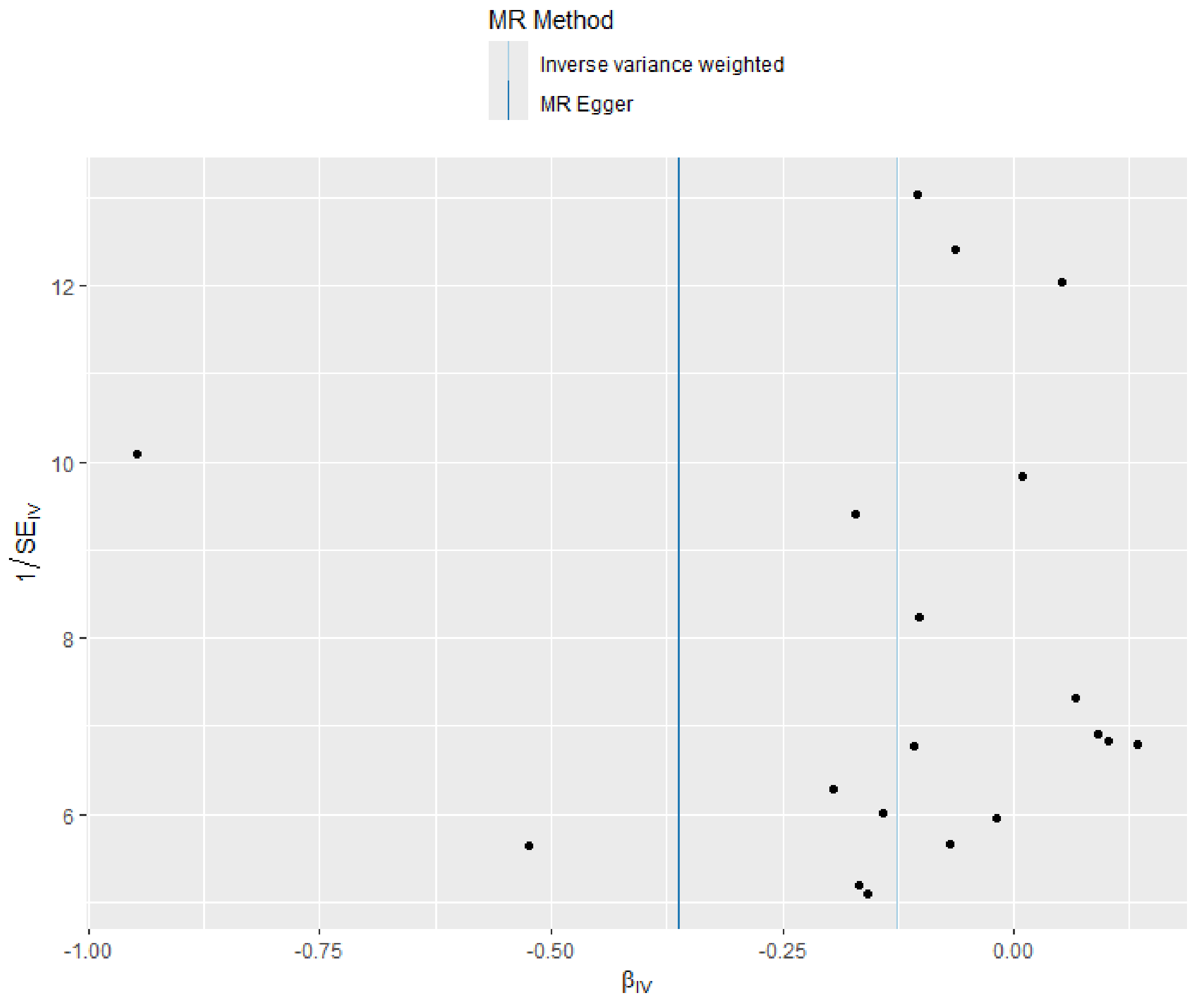


**Supplementary Figure 51 –** MR-funnel plot of diaphysis BMFF-Type 2 diabetes.

Vertical lines show the causal estimates using all SNPs combined into a single instrument for IVW random effects and MR-Egger methods.

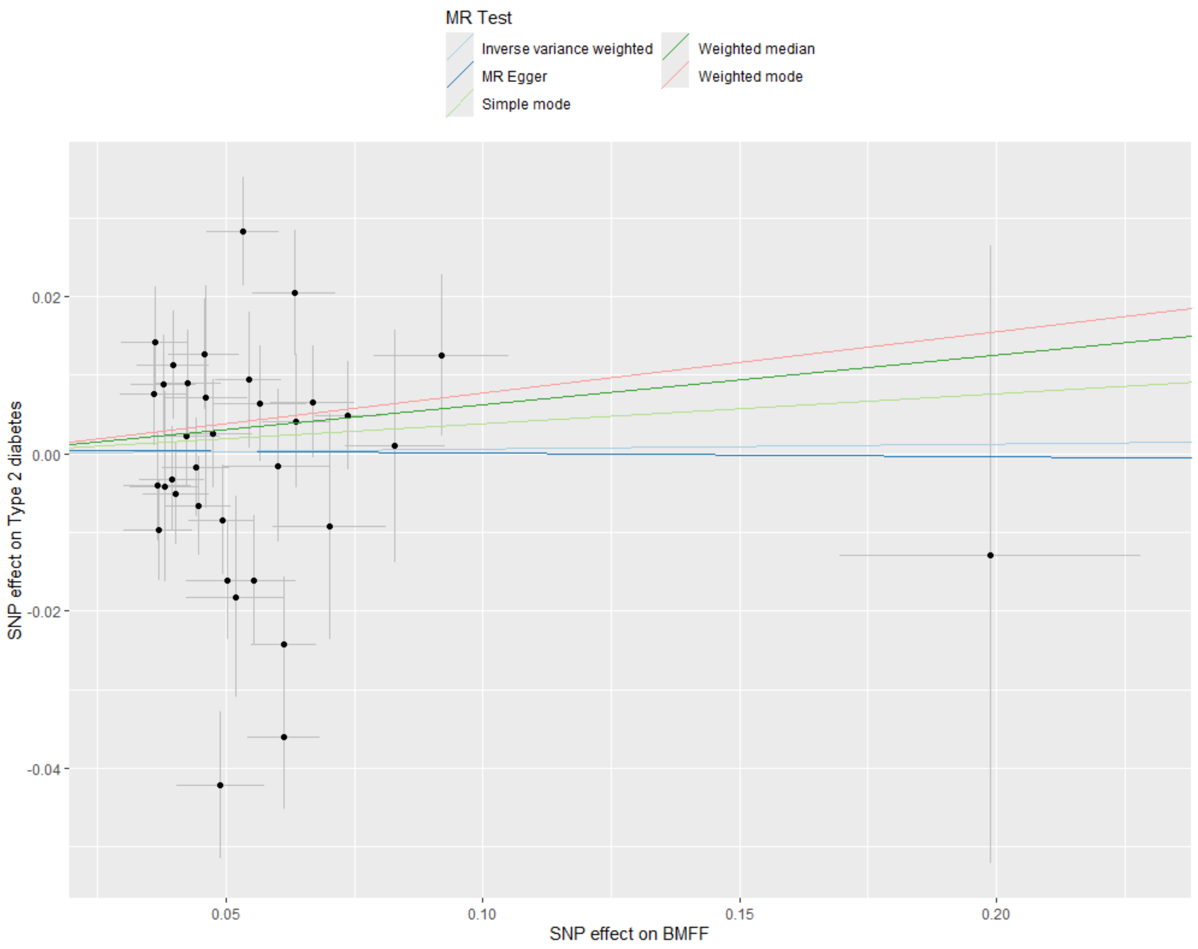


**Supplementary Figure 52 –** MR-scatter plot for spine BMFF-Type 2 diabetes.

Each dot represents a SNP (clump r2<0.001), with the x-axis showing the SNP effect on BMFF and the y-axis showing the SNP effect on type 2 diabetes. The fitted lines represent different MR estimation methods, including: inverse variance weighted (IVW; (light blue), MR Egger (dark blue), weighted mode (red), simple mode (light green), weighted median (dark green).

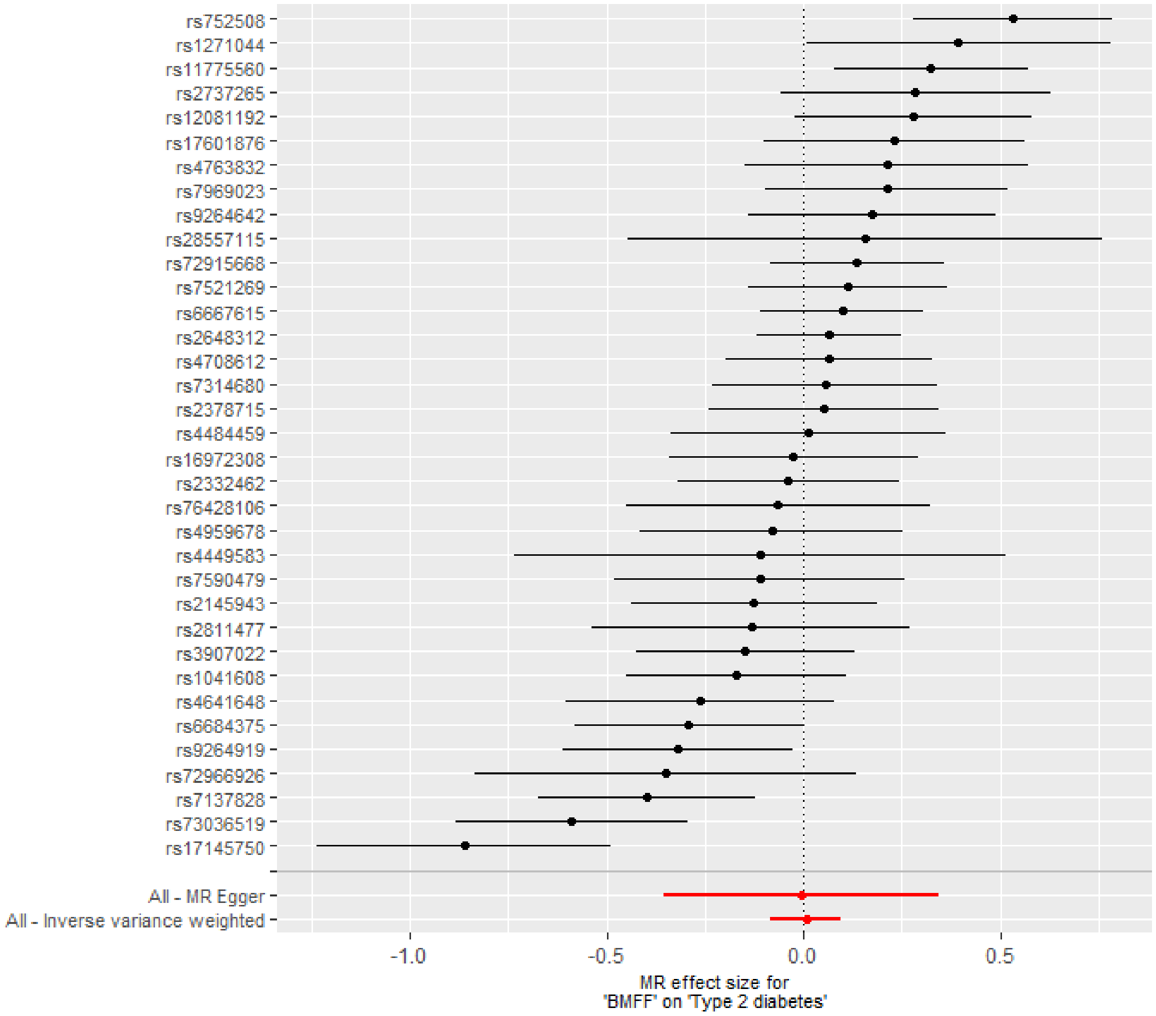


**Supplementary Figure 53 –** MR-forest plot for spine BMFF-Type 2 diabetes.

The x-axis represents the effect size with the corresponding 95% CIs. Each black point represents the log OR for type 2 diabetes per SD increase in BMFF, produced using each of the 'BMFF SNPs' (clump r2<0.001) as separate instruments, and red points showing the combined causal estimate using all SNPs together in a single instrument, using IVW random effects and MR-Egger. Horizontal lines denote 95% CI.

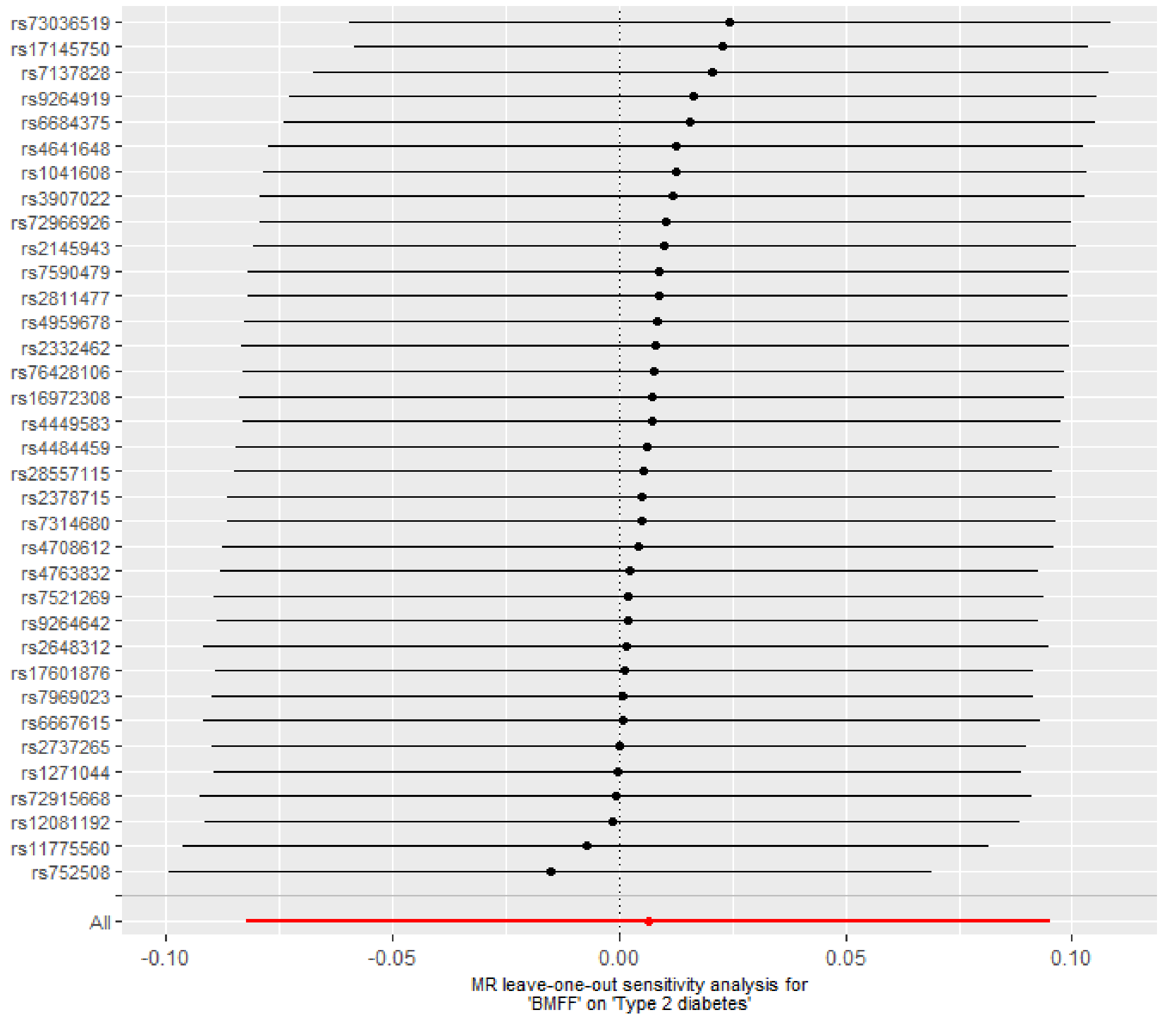


**Supplementary Figure 54 –** Forest plot of leave-one-out sensitivity results for spine BMFF-Type 2 diabetes.

Each black point represents the IVW MR method applied to estimate the causal effect of BMFF on type 2 diabetes excluding that particular variant from the analysis. The red point depicts the IVW estimate using all SNPs. There are no instances where the exclusion of one particular SNP leads to dramatic changes in the overall result.

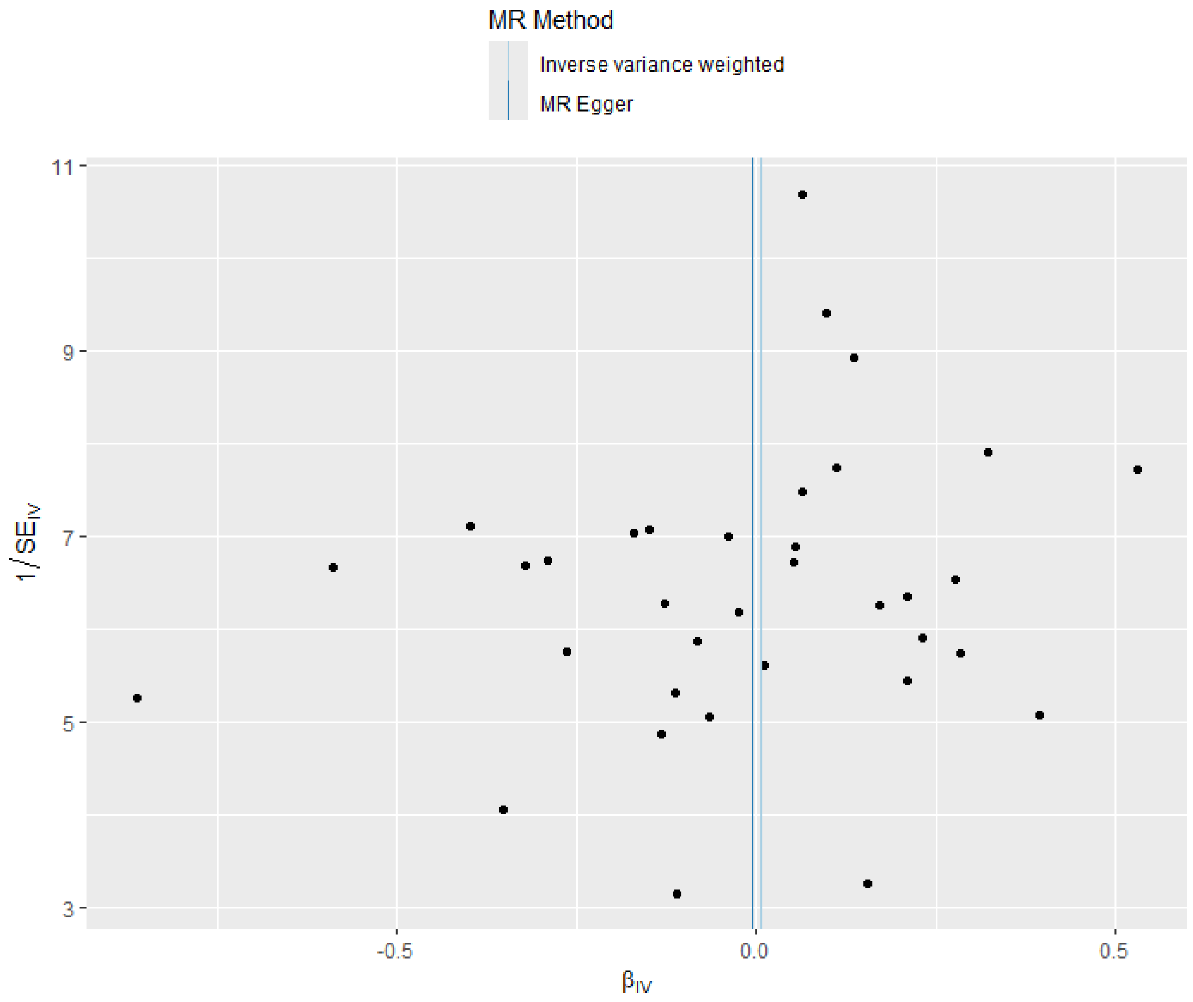


**Supplementary Figure 55 –** MR-funnel plot of spine BMFF-Type 2 diabetes.

Vertical lines show the causal estimates using all SNPs combined into a single instrument for IVW random effects and MR-Egger methods.

# Supplementary References


1. Denny, J.C., Ritchie, M.D., Basford, M.A., Pulley, J.M., Bastarache, L., Brown-Gentry, K., Wang, D., Masys, D.R., Roden, D.M. & Crawford, D.C. PheWAS: demonstrating the feasibility of a phenome-wide scan to discover gene-disease associations. *Bioinformatics* **26**, 1205-10 (2010).
2. Carroll, R.J., Bastarache, L. & Denny, J.C. R PheWAS: data analysis and plotting tools for phenome-wide association studies in the R environment. *Bioinformatics* **30**, 2375-6 (2014).
3. Li, X., Meng, X., Spiliopoulou, A., Timofeeva, M., Wei, W.Q., Gifford, A., Shen, X., He, Y., Varley, T., McKeigue, P., Tzoulaki, I., Wright, A.F., Joshi, P., Denny, J.C., Campbell, H. & Theodoratou, E. MR-PheWAS: exploring the causal effect of SUA level on multiple disease outcomes by using genetic instruments in UK Biobank. *Ann Rheum Dis* (2018).
4. Meng, X., Li, X., Timofeeva, M.N., He, Y., Spiliopoulou, A., Wei, W.Q., Gifford, A., Wu, H., Varley, T., Joshi, P., Denny, J.C., Farrington, S.M., Zgaga, L., Dunlop, M.G., McKeigue, P., Campbell, H. & Theodoratou, E. Phenome-wide Mendelian-randomization study of genetically determined vitamin D on multiple health outcomes using the UK Biobank study. *Int J Epidemiol* **48**, 1425-1434 (2019).
5. Zhang, X., Li, X., He, Y., Law, P.J., Farrington, S.M., Campbell, H., Tomlinson, I.P.M., Houlston, R.S., Dunlop, M.G., Timofeeva, M. & Theodoratou, E. Phenome-wide association study (PheWAS) of colorectal cancer risk SNP effects on health outcomes in UK Biobank. *Br J Cancer* **126**, 822-830 (2022).
6. Wang, L., Li, X., Montazeri, A., MacFarlane, A.J., Momoli, F., Duthie, S., Senekal, M., Eguiagaray, I.M., Munger, R., Bennett, D., Campbell, H., Rubini, M., McNulty, H., Little, J. & Theodoratou, E. Phenome-wide association study of genetically predicted B vitamins and homocysteine biomarkers with multiple health and disease outcomes: analysis of the UK Biobank. *Am J Clin Nutr* **117**, 564-575 (2023).
7. Yuan, S., Yu, L., Gou, W., Wang, L., Sun, J., Li, D., Lu, Y., Cai, X., Yu, H., Yuan, C., Zheng, J.S., Larsson, S.C., Theodoratou, E. & Li, X. Health effects of high serum calcium levels: Updated phenome-wide Mendelian randomisation investigation and review of Mendelian randomisation studies. *EBioMedicine* **76**, 103865 (2022).
8. Skrivankova, V.W., Richmond, R.C., Woolf, B.A.R., Yarmolinsky, J., Davies, N.M., Swanson, S.A., VanderWeele, T.J., Higgins, J.P.T., Timpson, N.J., Dimou, N., Langenberg, C., Golub, R.M., Loder, E.W., Gallo, V., Tybjaerg-Hansen, A., Davey Smith, G., Egger, M. & Richards, J.B. Strengthening the Reporting of Observational Studies in Epidemiology Using Mendelian Randomization: The STROBE-MR Statement. *Jama* **326**, 1614-1621 (2021).
9. Skrivankova, V.W., Richmond, R.C., Woolf, B.A.R., Davies, N.M., Swanson, S.A., VanderWeele, T.J., Timpson, N.J., Higgins, J.P.T., Dimou, N., Langenberg, C., Loder, E.W., Golub, R.M., Egger, M., Davey Smith, G. & Richards, J.B. Strengthening the reporting of observational studies in epidemiology using mendelian randomisation (STROBE-MR): explanation and elaboration. *Bmj* **375**, n2233 (2021).
