# Supplementary material for: Clinical implications of bone marrow adiposity identified by phenome-wide association and Mendelian randomization in the UK Biobank
Source: Nat Commun. 2025 Sep 23;16:8332. doi: 10.1038/s41467-025-63395-1 (PMC12457654; doi:10.1038/s41467-025-63395-1)
Supplement: Supplementary file 2 — Description of Additional Supplementary Files [file 41467_2025_63395_MOESM2_ESM.pdf]

Description of Additional Supplementary Files

File Name: Supplementary Data 1
Description: BMFF Obs-PheWAS sample QC for the four bone regions

File Name: Supplementary Data 2
Description: Summary of PheCODEs associated with BMFF in each disease group

File Name: Supplementary Data 3
Description: Overlap incident disease outcomes associated with BMFF in the Obs-PheWAS

File Name: Supplementary Data 4
Description: Associations between T2D and BMFF

File Name: Supplementary Data 5
Description: Disease outcomes associated with BMFF in the Obs-PheWAS (sensitivity analysis: incident + prevalent)

File Name: Supplementary Data 6
Description: Overlap disease outcomes associated with BMFF in the Obs-PheWAS (sensitivity analysis: incident + prevalent)

File Name: Supplementary Data 7
Description: Incident disease outcomes associated with BMFF in the Obs-PheWAS by sex group

File Name: Supplementary Data 8
Description: Overlap incident disease outcomes associated with BMFF in the Obs-PheWAS by sex group

File Name: Supplementary Data 9
Description: Disease outcomes associated with BMFF in the Obs-PheWAS (sensitivity analysis: incident + prevalent) by sex group

File Name: Supplementary Data 10
Description: Overlap disease outcomes associated with BMFF in the Obs-PheWAS (sensitivity analysis: incident + prevalent) by sex group

File Name: Supplementary Data 11
Description: BMFF PRS1 SNP QC for the four bone regions

File Name: Supplementary Data 12
Description: BMFF PRS2 SNP QC for the four bone regions

File Name: Supplementary Data 13
Description: BMFF PRS-PheWAS sample QC for the four bone regions

File Name: Supplementary Data 14
Description: SNPs used to generate BMFF PRS1 for the four bone regions

File Name: Supplementary Data 15
Description: SNPs used to generate BMFF PRS2 for the four bone regions

File Name: Supplementary Data 16
Description: Summary of PheCODEs associated with PRS in each disease group

File Name: Supplementary Data 17
Description: Overlap disease outcomes associated with BMFF in the PRS-PheWAS

File Name: Supplementary Data 18
Description: Disease outcomes associated with BMFF in the PRS-PheWAS by sex group

File Name: Supplementary Data 19
Description: Overlap disease outcomes associated with BMFF in the PRS-PheWAS by sex group

File Name: Supplementary Data 20
Description: Overlap disease outcomes associated with BMFF in the Obs-PheWAS (incident) and PRS-PheWAS

File Name: Supplementary Data 21
Description: PRS1 and PRS2 performance for the overlap disease outcomes associated with BMFF

File Name: Supplementary Data 22
Description: Leave-one-out results for exposure-BMFF and outcome-Osteoporosis (r^2<0.001)

File Name: Supplementary Data 23
Description: Leave-one-out results for exposure-BMFF and outcome-Fractures (r^2<0.001)

File Name: Supplementary Data 24
Description: Leave-one-out results for exposure-BMFF and outcome-Type 2 diabetes (r^2<0.001)

File Name: Supplementary Data 25
Description: Associations of genetically predicted BMFF levels with Osteoporosis in Mendelian randomisation (r^2<0.6)

File Name: Supplementary Data 26
Description: Leave-one-out results for exposure-BMFF and outcome-Osteoporosis ($r^2<0.6$)

File Name: Supplementary Data 27
Description: Associations of genetically predicted BMFF levels with outcomes in MR-PRESSO analysis

File Name: Supplementary Data 28
Description: Associations of genetically predicted BMFF levels with Fractures in Mendelian randomisation ($r^2<0.6$)

File Name: Supplementary Data 29
Description: Leave-one-out results for exposure-BMFF and outcome-Fractures ($r^2<0.6$)

File Name: Supplementary Data 30
Description: Associations of genetically predicted BMFF levels with Type 2 diabetes in Mendelian randomisation ($r^2<0.6$)

File Name: Supplementary Data 31
Description: Leave-one-out results for exposure-BMFF and outcome-Type 2 diabetes ($r^2<0.6$)

File Name: Supplementary Data 32
Description: Comparison of MR results for Osteoporosis in BMFF with/without BMI adjustment ($r^2<0.001$)

File Name: Supplementary Data 33
Description: Comparison of MR results for Fractures in BMFF with/without BMI adjustment ($r^2<0.001$)

File Name: Supplementary Data 34
Description: Comparison of MR results for T2D in BMFF with/without BMI adjustment ($r^2<0.001$)

File Name: Supplementary Data 35
Description: Incident cases of 'Fracture' PheCODE analyzed in main (incident) Obs-PheWAS

File Name: Supplementary Data 36
Description: Incident + prevalent cases of 'Fracture' PheCODE analyzed in sensitivity Obs-PheWAS

File Name: Supplementary Data 37
Description: Summary comparison of IV strength between two MR instrument sets

File Name: Supplementary Data 38
Description: Steiger filtering test

File Name: Supplementary Data 39
Description: BMFF-Osteoporosis: MR-steiger filtering test and MR-exclude BMD-associated SNPs cross-comparison (r^2<0.001)

File Name: Supplementary Data 40
Description: BMFF-Fractures: MR-steiger filtering test and MR-exclude BMD-associated SNPs cross-comparison (r^2<0.001)

File Name: Supplementary Data 41
Description: BMFF-T2D: MR-steiger filtering test and MR-exclude T2D-associated SNPs cross-comparison (r^2<0.001)

File Name: Supplementary Data 42
Description: Summary of published BMD GWASes with the full summary statistics in the largest sample size (https://www.ebi.ac.uk/gwas/)

File Name: Supplementary Data 43
Description: Summary of associations reported in the published BMD GWASes
