# Supplementary material for: Clinical implications of bone marrow adiposity identified by phenome-wide association and Mendelian randomization in the UK Biobank
Source: Nat Commun. 2025 Sep 23;16:8332. doi: 10.1038/s41467-025-63395-1 (PMC12457654; doi:10.1038/s41467-025-63395-1)
Supplement: Supplementary file 46 — Reporting Summary [file 41467_2025_63395_MOESM46_ESM.pdf]

nature portfolio

Corresponding author(s): William P Cawthorn and Evropi Theodoratou

Last updated by author(s): Jul 7, 2025

Reporting Summary

Nature Portfolio wishes to improve the reproducibility of the work that we publish. This form provides structure for consistency and transparency in reporting. For further information on Nature Portfolio policies, see our Editorial Policies and the Editorial Policy Checklist.

Statistics

For all statistical analyses, confirm that the following items are present in the figure legend, table legend, main text, or Methods section.

n/a	Confirmed	
☐	☒	The exact sample size (*n*) for each experimental group/condition, given as a discrete number and unit of measurement
☐	☒	A statement on whether measurements were taken from distinct samples or whether the same sample was measured repeatedly
☐	☒	The statistical test(s) used AND whether they are one- or two-sided<br>*Only common tests should be described solely by name; describe more complex techniques in the Methods section.*
☐	☒	A description of all covariates tested
☐	☒	A description of any assumptions or corrections, such as tests of normality and adjustment for multiple comparisons
☐	☒	A full description of the statistical parameters including central tendency (e.g. means) or other basic estimates (e.g. regression coefficient) AND variation (e.g. standard deviation) or associated estimates of uncertainty (e.g. confidence intervals)
☐	☒	For null hypothesis testing, the test statistic (e.g. *F*, *t*, *r*) with confidence intervals, effect sizes, degrees of freedom and *P* value noted<br>*Give P values as exact values whenever suitable.*
☒	☐	For Bayesian analysis, information on the choice of priors and Markov chain Monte Carlo settings
☒	☐	For hierarchical and complex designs, identification of the appropriate level for tests and full reporting of outcomes
☐	☒	Estimates of effect sizes (e.g. Cohen's *d*, Pearson's *r*), indicating how they were calculated

Our web collection on statistics for biologists contains articles on many of the points above.

Software and code

Policy information about availability of computer code

Data collection	Data collection was done separately by UK Biobank and not directly as part of this study. No software was used for data collection.
Data analysis	Our previous manuscript (https://doi.org/10.1016/j.csbj.2023.12.029) describes the methods for analysis of bone marrow fat fraction (BMFF) from UK Biobank MRI data. This includes:<br>- Deep learning for segmentation of bone marrow volumes in the spine, femoral head, total hip, and femoral diaphysis (code: Python). This code is available at DOI: 10.5281/zenodo.13959673.<br>- Code for sorting UK Biobank MRI data (prior to segmentation) and for fat fraction mapping (code: Matlab). This code is available at DOI: 10.5281/zenodo.13961316.<br><br>Availability of code used for PheWAS and MR analyses is described in the Methods section, including:<br>- Polygenic risk scores (PRS): LD proxies (r2 > 0.6) for significant SNPs passing quality control were generated using the LDproxy function in the LDlinkR package (https://github.com/CBIIT/LDlinkR (version 1.4.0) using 1000 Genomes Project Phase 3 as the LD reference panel.<br>- PheWAS analyses were performed using the 'PheWAS' package [https://github.com/PheWAS/PheWAS] (R version 4.4.1).<br>- Mendelian randomization (MR) was done using the 'TwoSampleMR', 'MR-PRESSO', and 'R2jags' packages [https://github.com/MRCIEU/TwoSampleMR] (R version 4.4.1).

For manuscripts utilizing custom algorithms or software that are central to the research but not yet described in published literature, software must be made available to editors and reviewers. We strongly encourage code deposition in a community repository (e.g. GitHub). See the Nature Portfolio guidelines for submitting code & software for further information.

Data

Policy information about availability of data

All manuscripts must include a data availability statement. This statement should provide the following information, where applicable:

- Accession codes, unique identifiers, or web links for publicly available datasets
- A description of any restrictions on data availability
- For clinical datasets or third party data, please ensure that the statement adheres to our policy

All data for BMFF and BM segmentation volumes have been uploaded to the UKBB (upload ID 5858), where they will be available under UKBB Category 105 (https://biobank.ndph.ox.ac.uk/ukb/label.cgi?id=105) to any individuals with an approved UKBB project. Researchers can apply for UKBB access via the UKBB Access Management System (https://ams.ukbiobank.ac.uk/ams/). PheCODEs were generated using data from the Phemap v1.2 (https://phewascatalog.org/phecodes_icd10). PRSs were constructed using data from our previous BMFF meta-GWAS (https://doi.org/10.1038/s41467-024-55422-4). To exclude any SNPs independently associated with bone mineral density or type 2 diabetes, we searched GWAS catalog (https://www.ebi.ac.uk/gwas/) to identify the most recently published GWAS with the largest sample size for BMD or type 2 diabetes; these studies are described in the manuscript. The remaining data are reported in the Supplementary Data files.

Research involving human participants, their data, or biological material

Policy information about studies with human participants or human data. See also policy information about sex, gender (identity/presentation), and sexual orientation and race, ethnicity and racism.

Reporting on sex and gender	We used biological sex in the study. It was determined based on the participants' genotypes. We conducted sex-stratified PheWAS for BMFF for the four bone regions.
Reporting on race, ethnicity, or other socially relevant groupings	In sample quality control, we sub-grouped participants based on their ancestry as 'White' using the UKBB data-field 22006 ("Genetic ethnic grouping"). PRS-PheWAS was done only in participants of white European ancestry, because the GWAS data used to generate the PRSs are from this ethnic grouping.
Population characteristics	Population characteristics are described in Table 1, Table 3, Supplementary Data 1, and Supplementary Data 13
Recruitment	Recruitment of participants for the UK Biobank is described by Sudlow et al 2015 (doi: 10.1371/journal.pmed.1001779) and Littlejohns et al 2020 (doi: 10.1038/s41467-020-15948-9), which we cite in the main manuscript.
Ethics oversight	UKBB has approval from the North West Multi-Centre Research Ethics Committee (MREC) as a Research Tissue Bank (RTB) approval (Ref: 11/NW/0382). Data for this work were obtained under the approved UKBB project application (ID 48697).

Note that full information on the approval of the study protocol must also be provided in the manuscript.

Field-specific reporting

Please select the one below that is the best fit for your research. If you are not sure, read the appropriate sections before making your selection.

☒ Life sciences ☐ Behavioural & social sciences ☐ Ecological, evolutionary & environmental sciences

For a reference copy of the document with all sections, see nature.com/documents/nr-reporting-summary-flat.pdf

Life sciences study design

All studies must disclose on these points even when the disclosure is negative.

Sample size	Sample sizes were not predetermined but instead resulted from the availability of UK Biobank MRI data and the generation of reliable segmentation outputs from deep learning.
Data exclusions	We describe data exclusions in the Methods, within the subsections named 'BMFF polygenic risk score', 'Phenome-wide association analyses'. and 'Mendelian randomization analyses'. These exclusions relate to qualtiy control and/or sensitivity analyses for each of these methods.
Replication	Each analysis (e.g. Obs-PheWAS, PRS-PheWAS, MR) was not independently replicated because they relied on analyses within the UK Biobank cohort. However, these three analyses tested our hypothesis in different ways, providing independent results from which to draw overall conclusions.
Randomization	PRSs and Mendelian randomization are methods that use alleles, which are randomly allocated at conception, as instrumental variables to estimate the causal effect of an exposure on an outcome. Therefore, these are less prone to bias of confounding and reverse causation associated with observational studies.
Blinding	This was an observational design: no blinding was used.

Reporting for specific materials, systems and methods

We require information from authors about some types of materials, experimental systems and methods used in many studies. Here, indicate whether each material, system or method listed is relevant to your study. If you are not sure if a list item applies to your research, read the appropriate section before selecting a response.

Materials & experimental systems

n/a	Involved in the study
☒	☐ Antibodies
☒	☐ Eukaryotic cell lines
☒	☐ Palaeontology and archaeology
☒	☐ Animals and other organisms
☒	☐ Clinical data
☒	☐ Dual use research of concern
☒	☐ Plants

Methods

n/a	Involved in the study
☒	☐ ChIP-seq
☒	☐ Flow cytometry
☒	☐ MRI-based neuroimaging

Plants

Seed stocks	N/A
Novel plant genotypes	N/A
Authentication	N/A
